# Deciphering potential pharmacological mechanism of Sha-Shen-Mai-Dong decoction on primary Sjogren’s syndrome

**DOI:** 10.1186/s12906-021-03257-7

**Published:** 2021-03-01

**Authors:** Yuepeng Jiang, Xiaoxuan Zhao, Jie Yu, Qiao Wang, Chengping Wen, Lin Huang

**Affiliations:** 1 College of Basic Medical Sciences, Zhejiang Chinese Medical University, Hangzhou, 310058 China; 2 Heilongjiang University of Chinese Medicine, Harbin, 150040 China

**Keywords:** Sha-Shen-Mai-Dong decoction, pSS, Network pharmacology, Target prediction, Immunologic mechanism

## Abstract

**Background:**

Sha-Shen-Mai-Dong decoction (SSMD) is a classical prescription widely used in primary Sjogren’s Syndrome (pSS) therapy. This study aims to explore the potential pharmacological mechanism of SSMD on pSS.

**Methods:**

Active components of SSMD were obtained from Traditional Chinese Medicine Integrative Database and Traditional Chinese Medicine Systems Pharmacology databases and targets of SSMD were predicted by Pharmmapper and STITCH database. Gene Ontology (GO), Kyoto Encyclopedia of Genes and Genomes (KEGG) analysis were carried out to explore the function characteristics of SSMD. The expression matrix of microarray of pSS was obtained from Gene Expression Omnibus and we obtained 162 differentially expressed genes (DEGs). Protein-protein interaction (PPI) networks were constructed to identify the hub targets. Principal component analysis (PCA) and molecular docking were conducted to further elucidate the possibility of SSMD for pSS.

**Results:**

SSMD contained a total of 1056 active components, corresponding to 88 targets, among which peripheral myelin protein 2(PMP2), androgen receptor (AR) and glutamic acid decarboxylase 1(GAD1) are associated with multiple active components in SSMD and may be the core targets. Moreover, these targets were closely related to tissue pathological injury in SS, such as lacrimal gland, salivary gland and nervous system injury. GO and KEGG analysis showed that 88 targets enriched in REDOX process, transcriptional regulation and negative regulation of apoptosis process. Besides, SSMD may influence the cell proliferation, gene transcription through regulating Ras and cAMP-related signaling pathways. In addition, SSMD may show effects on immune regulation, such as macrophage differentiation, Toll-like receptor 4 signaling pathway and T-helper 1 in SS. Moreover, PPI network suggested that FN1, MMP-9 may be the hub targets in SSMD. Result of PCA and molecular docking analysis further determined the feasibility of SSMD in treating pSS.

**Conclusion:**

SSMD can regulate multiple biological processes by virtue of its multiple active components, thus showing prominent advantage in the treatment of pSS. The discovery of active ingredients and targets in SSMD provides valuable resources for drug research and development for pSS.

**Supplementary Information:**

The online version contains supplementary material available at 10.1186/s12906-021-03257-7.

## Background

Sjogren syndrome (SS) is a systemic autoimmune disease characterized by chronic inflammation in exocrine glands, especially in salivary glands and lacrimal glands [1, 2], which impacts glandular secretion and leads to dryness of mouth, eyes and other mucosal surfaces [3]. SS can be divided into two categories, i.e. primary SS (pSS) and secondary SS, based on whether patients suffer from other connective tissue disease. The incidence of SS ranges from 0.05 to 0.5% according to environment and race, and women are more susceptible than men [4, 5]. At present, the mechanism of pSS is not yet clear [6]. Most scholars believe that genetic susceptibility, environmental exposure and disorder of autoimmune regulation cooperatively trigger a series of autoimmune reactions, affecting the function of columnar epithelial cells in exocrine glands and leading to lesions in salivary and lacrimal glands [4, 7]. In addition to dryness, pSS can also cause damage to the skin, kidney, respiratory system and other systems [8, 9].

Until now, pathogenesis-targeted solution for pSS is still lacking. Clinical approaches include local alternative and systemic therapies with artificial tears, antimicrobial mouthwashes, immunosuppressants, glucocorticoid, nonsteroidal anti-inflammatory drugs and biological agents, etc. [10, 11] The recommended therapeutic schedule could alleviate symptom and delay the progression of pSS to some extent, however, high recurrence rate and severe side effects still raise eager concern worldwide [12]. Therefore, exploring effective therapeutic regimens with little side effects are imperative.

Sha-Shen-Mai-Dong (SSMD) decoction was proposed by Wu Jutong, a famous doctor in the Qing Dynasty. It contains 7 kinds of herbs, and the recommended recipe is as follows: *Glehnia littoralis*(9 g), *Polygonatum odoratum* (6 g), *Glycyrrhiza uralensis* (3 g), *Morus alba* (4.5 g), *Ophiopogon japonicus* (9 g), *Lablab niger* (4.5 g), *Trichosanthes kirilowii* (4.5 g). The full scientific species (Latin binomial nomenclature) names of all ingredients of SSMD obtained from the Traditional Chinese Medicines Integrated Database (TCMID) were shown in Table 1. An observational study by Lim RJ et al. [13] found that SSMD decoction can improve xerostomia in head and neck cancer patients. Presently, SSMD is widely used in pSS in China with remarkable efficacy and little side-effects. Wang Y et al. [14] found that *Ophiopogon japonicus* polysaccharides, as the active component of SSMD, could significantly improve the flow rate of saliva and reduce the level of inflammatory factors in the autoallergic mouse model for SS when compared with hydroxychloroquine. However, it is difficult to be fully understood the pharmacological mechanism through traditional methods. With the rapid development of bioinformatics, network pharmacology is considered to be a promising method to predict the underlying mechanism from a systems perspective and at the molecular level so as to provide clues and directions for the follow-up research. It superiors to the previous simplified drug development model of “one drug, one target, one disease”. This paper aims to clarify the potential mechanism of SSMD in the treatment of pSS through in-depth analysis of its active components, potential key targets, and biological pathways. This article contributes to our understanding for the intangible biological processes of SSMD on SS and provides new therapeutic options for the treatment of pSS.

**Table 1:** The full scientific species (Latin binomial nomenclature) names of all ingredients of Sha-Shen-Mai-Dong Decoction

Chinese name	English name	Latin binomial nomenclature name
Bei Sha Shen	Coastal Glehnia	*Glehnia littoralis*
Mai Dong	Liriope Equivalent plant: *Liriope spicata* var. prolifera	*Ophiopogon japonicus*
Yu Zhu	Fragrant Solomonseal Equivalent plant: Polygonatum prattii	*Polygonatum odoratum*
Sang Ye	White Mulberry Leaf Equivalent plant: Morus mongolica, Morus australis, Morus cathayana	*Morus alba*
Bian Dou	Niger Bean	*Lablab niger*
Tian Hua Fen	Mongolian Snakegourd Root	*Trichosanthes kirilowii*
Gan Cao	Ural Licorice Equivalent plant: Glycyrrhiza inflata, *Glycyrrhiza glabra*, Glycyrrhiza kansuensis, Glycyrrhiza aspera, Glycyrrhiza yunnanensis, Glycyrrhiza squamulosa	*Glycyrrhiza uralensis*

## Methods

### Collection and screening of active ingredients of SSMD

The herbs included in SSMD were used as keywords to inquire and screen active components in TCMSP database (http://tcmspw.com/tcmsp.php) [15] and TCMID database (http://www.megabionet.org/tcmid/) [16], including *Glehnia littoralis*, *Polygonatum odoratum*, *Glycyrrhiza uralensis*, *Morus alba*, *Ophiopogon japonicus*, *Lablab niger*, *Trichosanthes kirilowii*. Oral bioavailability (OB) is a key parameter to evaluate whether a drug exerts development value, as well as an effective indicator to appraise the clinical efficacy of traditional Chinese medicine (TCM). And drug likeness (DL) is currently widely used to assess the possible likeness of a leading compound in herb as a independent drug. Thus, we screened the active compounds of SSMD under the conditions of OB > 30% and DL > 0.18.

### Prediction and screening of targets of SSMD active components

After screening the active components of SSMD through TCMSP and TCMID databases, the active components were uploaded to PubChem (https://www.ncbi.nlm.nih.gov/) [17], and the 2D structures of the active components were found and then we uploaded them into the pharmmapper website (http://lilab-ecust.cn/pharmmapper/submitfile. html) [18] to predict the targets. Besides, Uniprot (https://www.uniprot. org/uploadlists/) [19] was used to standardize the predicted targets and expressed them in the form of gene ID. In order to increase the credibility of the targets, STITCH database (http://stitch.embl.de/), which is a frequently-used database for retrieving the interaction between predicted compounds and target proteins, was also utilized to predict the targets. Finally, the Venn diagram was made and the intersection of the two databases is regarded as the final targets of SSMD.

### Screening of DEGs in pSS microarray

Gene Expression Omnibus (GEO)(http://www.ncbi.nlm.nih.gov/geo) is a public functional genomic database that stores high-throughput gene expression data, chips and microarrays. CEL file of pSS was downloaded from GEO (GSE97614). Quality control was applied by affy and simpleaffy package in R. Microarrays of pSS were obtained, and the DEGs were analyzed and annotated by GEO2R (http://www.ncbi.nlm.nih.gov/ GEO/GEO2R). Absolute value of logFC> 1 and *p*-value< 0.05 were taken as the screening conditions.

### Construction and visualization of protein-protein interaction

PPIs were collected from HPRD (http://www.hprd.org/) and Biogrid (https://thebiogrid.org/) to construct the association network. The diagrams were visualized by Cytoscape 3.7.1 software.

### Bioinformation enrichment analysis of target genes

**Fig. 1:**
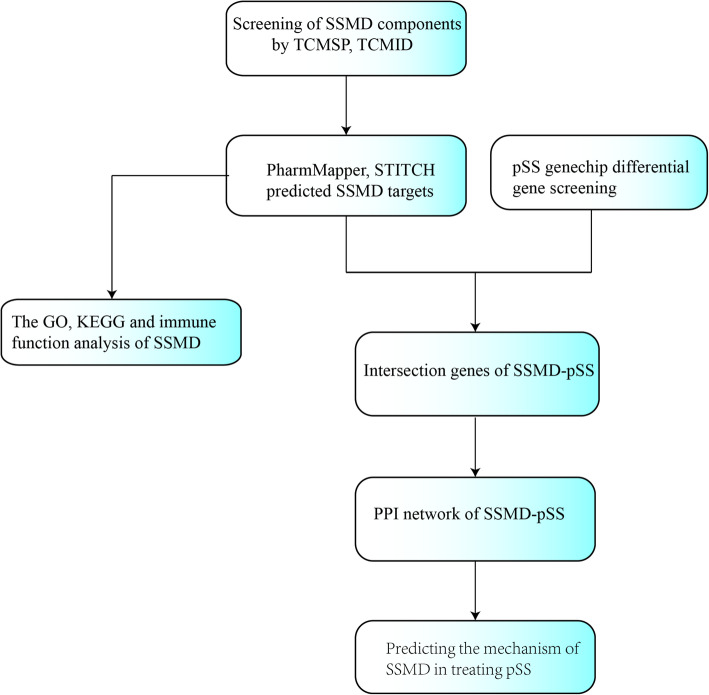
The network pharmacologic analysis flow chart of SSMD

DAVID database (https://david.ncifcrf.gov/) was used to carry out GO and KEGG pathway enrichment analysis, of which the species was limited as “*Homo sapiens*”. GO and KEGG analyses can not only annotate the function of genes, but also reflect the signaling pathways in which genes are involved, thus fully revealing the nature of biological events in the organism. The network pharmacologic analysis flow chart of SSMD was shown in Fig. 1.

### Principal component analysis (PCA) of SSMD active ingredients and FDA recommended drugs for pSS

To further determine the feasibility of SSMD in the treatment of pSS, we performed PCA on SSMD active ingredients and FDA recommended drugs for pSS. The Drugbank (www.drugbank.ca/drugs/) database was utilized to screen FDA-recommended drugs. The physicochemical parameters of these drugs as well as active ingredients in SSMD was obtained from the Pubchem database, including Molecular Weight, Hydrogen Bond Donor Count, Hydrogen Bond Acceptor Count, Rotatable Bond Count, Exact Mass, Topological Polar Surface Area, etc. Simca 14.1 was used for PCA according to the above parameters.

### Molecular docking analysis

The 2D structures of SSMD active components and their key targets were respectively downloaded from the Pubchem database and the PDB database (http://www.rcsb.org/) and these molecular were dehydrated and hydrogenated. Then, autoDOCK 1.5.7 software was used to complete molecular docking analysis. And the binding energy of the medicinal ingredients and the targets was compared with that of the original ligand. The binding energy (affinity) less than − 7.0 kcal·mol^− 1^ indicated good binding activity [20], and the lower the binding energy was, the better the docking effect was.

## Results

### Formulation, active ingredients and targets of SSMD

SSMD consists of seven herbs, i.e. *Glehnia littoralis*, *Polygonatum odoratum*, *Glycyrrhiza uralensis*, *Morus alba*, *Ophiopogon japonicus*, *Lablab niger*, *Trichosanthes kirilowii*. In order to reveal the potential therapeutic mechanism of this decoction, seven herbs of SSMD were input into TCMSP and TCMID databases, and 675 and 497 active ingredients were respectively obtained from the two databases. We combined the results after eliminating duplicates and obtained a total of 1060 effective ingredients (Supplement Table S1). Specifically, *Glehnia littoralis* has 104 components including adenosine, Imperatorin, and Xanthotoxin, among of which adenosine can induce vasodilation, inhibit the release of norepinephrine in sympathetic nerve endings and exhibit anti-inflammatory effects under stress [21]. Adenosine has been shown to inhibit the progression of RA by regulating the activation of all cell types involved and inhibiting inflammatory responses in fibrosis and scar formation [22]. Imperatorin (IMP), a natural form of coumarin, can exert antioxidant and anti-inflammatory effects [23] and inhibit translocation of NF- κB in organ damage [24]. *Lablab niger* contains 28 different ingredients, including β-carotene, nicotinic acid, asparaginase, etc. Among them, β-carotene exists widely in fruits and vegetables, and shows a significant antioxidant effect [25]. Researches have showed that the oxidative stress markers in patients with pSS are significantly higher than those in the healthy control group [26]. Nicotinic acid is an essential vitamin and can be converted into niacinamide (NAm) in the body which is well-known to express cell-protective and anti-inflammatory substances. Studies have shown that NAm inhibits the responses of primary B lymphocytes to multiple ligands at pharmacological concentrations in mice, indicating its potential role in regulating antibody-mediated autoimmune diseases [27]. *Polygonatum odoratum* contains 73 active ingredients, including yamogenin and convallamarin, and raw licorice contains 400 active ingredients, including ferulic acid, glycyrol, β-sitosterol, etc. Ferulic acid is a recognized compound with anti-inflammatory activity [28], and it has been proved to be effective in a variety of diseases. Studies have found that it can significantly improve lipid profile, oxidative stress indicators (malondialdehyde), and inflammatory factors (HS-CRP and TNF-α) in a randomized, double-blind, placebo-controlled trial [29]. *Morus alba* contains 318 different components, including astragalin, chlorogenic acid, morin, etc. Modern pharmacological studies have shown that *Morus alba* can inhibit the elevation of endoplasmic reticulum stress markers and significantly reduce myocardial fibrosis in myocarditis model rats [30]. And its active components astragalin significantly reduced the level of inflammatory cytokines in LPS-induced macrophages RAW 264.7.1, and inhibited the activation of NF-κB. Thus, we can conclude that *Morus alba* plays a regulatory role in autoimmune diseases. *Ophiopogon japonicus* consists 56 active ingredients, including ophiopogonina, uridine, etc. *Trichosanthes kirilowii* consists of 81 active constituents, including citrulline, ethyl hexadecanoate, etc. Experiment displayed that macrophages treated with ethyl hexadecanoate showed higher anti-inflammatory activity. To sum up, the active compounds contained in SSMD exhibit various pharmacological properties, such as anti-oxidative stress, anti-fibrosis, and regulation of immune activity, which are consistent with the pathological mechanism of autoimmune diseases, especially SS.

Among these different components, 76 were found in more than one herb, and the common constituents tend to be key components for the biological function of SSMD. We further identified shared ingredients in herb pairs. Among the seven herbs, *Polygonatum odoratum* and *Morus alba* share 15 common ingredients, such as chlorogenic acid, Isoquercitrin, Scopoletin, etc. *Trichosanthes kirilowii* and *Morus alba* share 6 components, such as Stigmasterol, Arachic acid and OLEIC acid, etc. The above common components are also associated with pSS-related pathological mechanisms. For instance, these components can inhibit the production of prostaglandin E2 (PGE2), Chlorogenic acid [31] in inflammation response and regulate the immunomodulatory activities [32–34]. The interaction of these herbs in SSMD can systematically and synergistically promote different biological reactions in the human body and shows potential therapeutic effect on SS with complex pathological mechanism.

**Fig. 2:**
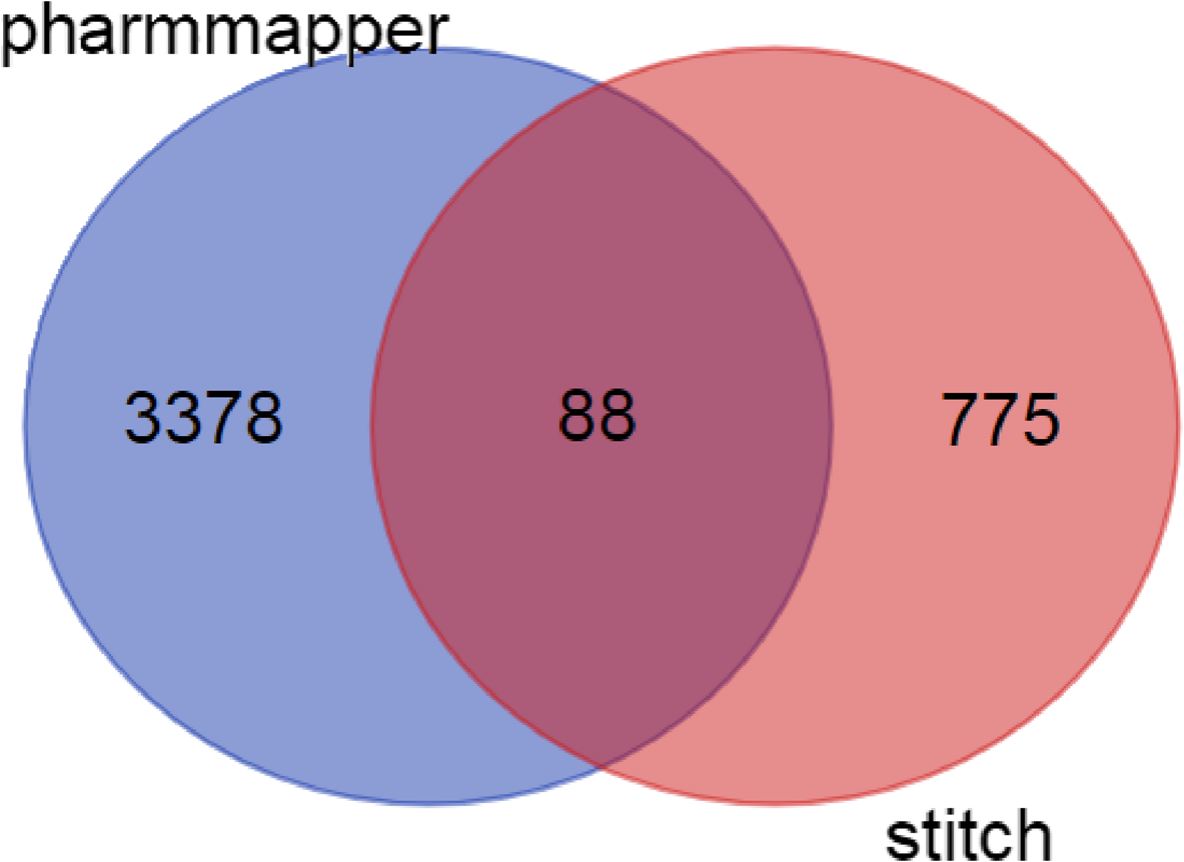
Target prediction of pharmaceutical components by pharmmapper and STITCH

Next, we used pharmmapper and STITCH to predict the targets of SSMD and obtained 3466 predicted targets from pharmmapper, and 863 from STITCH respectively with high correlation (threshold> 700). In order to increase the credibility of target acquisition, we intersected the targets. Finally, a total of 88 targets were obtained as the target of SSMD (Fig. 2.). The relationship between the effective compounds of SSMD and the corresponding targets were shown in Supplement Table S2. 88 targets were associated with 292 active components. Among them, homosapiens peripheral myelin protein 2(PMP2, also known as P2) was related to 159 different compounds. P2 belongs to the family of cytoplasmic fatty acid binding proteins (FABPs), which is an important part of the myelin sheath of peripheral nervous system (PNS). P2 accounts for 15% of the total myelin sheath protein [35] and participates in myelin sheath assembly and turnover, and myelin membrane stablizaion [36]. It has been shown that P2 mediates the occurrence and development of chronic inflammatory demyelinating multiple radicular neuropathy [37] which is related to peripheral neuropathy damage in SS [38]. This result shows that SSMD may be effective in the prevention of peripheral neuropathy in patients with SS. Androgen receptor (AR), a part of the super gene receptor family, is associated with 121 different compounds. Many studies have confirmed that the deficiency of sex steroid receptor is related to the incidence, progress, severity and sex related morbidity of various autoimmune diseases. The mechnism is probably related with glandular remodeling [39, 40]. The findings of these studies provide evidence for us to treat pSS by intervening in the action pathway of steroids. Also, AR is exactly the target of active components of SSMD, considering Stephen M et al. found that the mRNA sequence of SS mice was consistent with that of the standard mice, and there was no functional polymorphism [41], we speculated that SSMD might affect the modification of androgen receptor gene after translation, thus affecting the activity of binding with androgen, and then affecting its target control on gland cells. Another target, glutamic acid decarboxylase 1 (GAD1), can be associated with 93 different compounds. GAD1 is the key speed limiting enzyme for the synthesis of GABA which is an important inhibitory neurotransmitter. The existing research shows that GAD is closely related to the occurrence of autoimmune diseases, neurological diseases and chronic pain. Ikeda et al. [42] reported that there are autoantibodies against GAD in the serum of SS patients. In addition, the decreased activity of GAD will damage the inhibition of GABA synapse, leading to persistent inflammation and symptoms of neuropathic pain [43]. GAD may be used as a potential drug target to intervene SS myalgia. We found 93 different compounds in SSMD that can target GAD, which contains huge pharmaceutical resources, waiting for us to develop. Through the above analysis, we can clearly show that multiple active ingredients in the decoction interact with multiple targets with specific functions to treat the disease.

### The GO and KEGG analysis of SSMD

We further conducted GO and KEGG enrichment analysis for the 88 targets through bioinformation annotation database (DAVID). The specific results of GO functional enrichment consisted of analysis on biological process (BP), cellular component (CC), and molecular function (MF). GO analysis showed that 88 targets were widely distributed, mainly located in exosome, cytoplasm, mitochondria, endoplasmic reticulum (ER), and the perinuclear region of the cytoplasm (Table 2). Moreover, the 88 targets show a variety of molecular functions such as combining with zinc, iron, heme iron ion and enzyme, and are capable of influencing a variety of biological processes, such as REDOX process, transcription regulation, apoptosis, drug reaction as well as signal transduction and so on. The GO analysis shows us the versatility of the decoction. For instance, studies have illustrated that zinc ions can combine with the matrix metalloproteinases (MMPs), a zinc ions dependence of proteolytic enzyme, which promotes the degradation of extracellular matrix (ECM), and then leads to structural and functional abnormalities of exocrine glands. This is closely related to the pathology of SS [44]. In combination with the literature analysis, we speculated that SSMD may affect the biological binding of Zn to MMP, thus blocking the destruction of exocrine glands. In addition, pSS is reported to be manifested by excessive oxidative stress, and ROS and RNS play mediating and regulating roles in ER stress. Moustaka et al. found that in SS patients, ER cisternas in salivary gland epithelial cells are significantly magnified [45], which could be coupled with the signal transduction pathway of intracellular inflammatory response and the signal of apoptosis [46], thus promoting the pathological progression of pSS. GO analysis showed that the targets of SSMD were enriched in the ER, REDOX process and apoptosis regulation. This finding was consistent with the pathological mechanism of SS studied in the literatures [4, 6, 7].

**Table 2:** Results of Go analysis

Category	Term	Count	PValue
GOTERM_CC_DIRECT	GO:0070062 ~ extracellular exosome	39	1.93E-10
GOTERM_CC_DIRECT	GO:0005829 ~ cytosol	37	2.78E-07
GOTERM_CC_DIRECT	GO:0005576 ~ extracellular region	22	1.37E-05
GOTERM_CC_DIRECT	GO:0005615 ~ extracellular space	21	3.37E-06
GOTERM_BP_DIRECT	GO:0055114 ~ oxidation-reduction process	20	1.13E-10
GOTERM_MF_DIRECT	GO:0008270 ~ zinc ion binding	15	0.002237722
GOTERM_CC_DIRECT	GO:0005739 ~ mitochondrion	14	0.009333458
GOTERM_BP_DIRECT	GO:0045944 ~ positive regulation of transcription from RNA polymerase II promoter	13	0.004150299
GOTERM_BP_DIRECT	GO:0007165 ~ signal transduction	13	0.01496277
GOTERM_MF_DIRECT	GO:0042803 ~ protein homodimerization activity	11	0.003921296
GOTERM_MF_DIRECT	GO:0042802 ~ identical protein binding	11	0.004694948
GOTERM_CC_DIRECT	GO:0005789 ~ endoplasmic reticulum membrane	11	0.007295149
GOTERM_MF_DIRECT	GO:0020037 ~ heme binding	10	3.18E-08
GOTERM_BP_DIRECT	GO:0042493 ~ response to drug	10	2.52E-05
GOTERM_MF_DIRECT	GO:0019899 ~ enzyme binding	10	4.92E-05
GOTERM_BP_DIRECT	GO:0043066 ~ negative regulation of apoptotic process	10	5.28E-04
GOTERM_CC_DIRECT	GO:0048471 ~ perinuclear region of cytoplasm	10	0.002603752
GOTERM_MF_DIRECT	GO:0005506 ~ iron ion binding	9	1.11E-06
GOTERM_MF_DIRECT	GO:0016491 ~ oxidoreductase activity	9	8.12E-06
GOTERM_MF_DIRECT	GO:0005102 ~ receptor binding	9	4.34E-04

The 88 SSMD targets also enriched in 47 KEGG pathways. We classified those pathways into four categories, namely human diseases, process of environmental information, metabolic pathways (including amino acid and fat metabolism, steroid hormone metabolism and drug metabolism) and signal transduction (RAS signaling pathway and cAMP signaling pathway). The top 20 pathways are shown in Table 3. One of them is arachidonicacid (AA) pathway. AA is an essential unsaturated fatty acid in human body. Cytochrome P450 (CYP) catalysed AA to produce epoxyeicosatrienoic acids (EETs), ROS and Hyeroxyeicosatet- raenoicacids (HETEs) [47]. By inhibiting the nuclear translocation or phosphorylation of NF-κB, EETs can decrease the adhesion of inflammatory cells to endothelial cells and decrease the chronic soakage of inflammatory cells [48]. Besides, many evidences show that pSS manifests as NF-κB signal transduction and chronic inflammation [49]. According to the above analysis, we speculate that SSMD may block NF-κB signaling pathway by correcting the imbalance of AA- CYP enzyme metabolism.

**Table 3:** Results of KEGG analysis

Category	Term	Count	PValue
KEGG_PATHWAY	hsa01100:Metabolic pathways	37	1.43E-09
KEGG_PATHWAY	hsa05200:Pathways in cancer	13	0.001005911
KEGG_PATHWAY	hsa05204:Chemical carcinogenesis	8	2.32E-05
KEGG_PATHWAY	hsa04726:Serotonergic synapse	8	1.89E-04
KEGG_PATHWAY	hsa00590:Arachidonic acid metabolism	7	4.74E-05
KEGG_PATHWAY	hsa00982:Drug metabolism - cytochrome P450	7	8.79E-05
KEGG_PATHWAY	hsa05205:Proteoglycans in cancer	7	0.021410917
KEGG_PATHWAY	hsa04510:Focal adhesion	7	0.024352026
KEGG_PATHWAY	hsa04014:Ras signaling pathway	7	0.036086511
KEGG_PATHWAY	hsa00980:Metabolism of xenobiotics by cytochrome P450	6	0.00120795
KEGG_PATHWAY	hsa05161:Hepatitis B	6	0.02059476
KEGG_PATHWAY	hsa05152:Tuberculosis	6	0.043346677
KEGG_PATHWAY	hsa04024:cAMP signaling pathway	6	0.064244497
KEGG_PATHWAY	hsa01130:Biosynthesis of antibiotics	6	0.080859796
KEGG_PATHWAY	hsa00591:Linoleic acid metabolism	5	2.52E-04
KEGG_PATHWAY	hsa00410:beta-Alanine metabolism	5	3.28E-04
KEGG_PATHWAY	hsa00350:Tyrosine metabolism	5	5.28E-04
KEGG_PATHWAY	hsa05219:Bladder cancer	5	9.73E-04
KEGG_PATHWAY	hsa00330:Arginine and proline metabolism	5	0.002054354
KEGG_PATHWAY	hsa00140:Steroid hormone biosynthesis	5	0.003544481

In terms of signal transduction, the KEGG analysis reveals that SSMD can act on the 3 ‘5’ - monophosphate (cAMP) signal pathway, which plays a wide range of regulatory roles in cellular activities [50]. It has been shown that phosphorylated cAMP response element binding protein (p-CREB) can bind to cAMP response element (CRES) in DNA and give rise to the upregulation of aquaporin 5(AQP5) which distributes in the lip, sublingual, submaxillary and parotid glands [51, 52], and acts as a water channel to increase the salivary flow rate [53]. Studies also find that AQP5 are decreased significantly in NOD mice, a mature disease model of SS [54]. We speculate that one of the mechanisms for SSMD to treat SS is to promote cAMP phosphaorylation and upregulate the expression of AQP5. Another enriched signaling pathway is Ras signaling pathway, which is a monomeric GTP binding protein. It can transfer mitogen signal from serous membrane to nucleus through Raf/ MEK/ ERK kinase cascade, promoting cell proliferation and survival. Mariette et al. [55] believed that the loss of Ras overexpression in acinar cells might be one of the key mechanisms of cell apoptosis and acinar destruction in SS. At present, many studies have proved that there is a crosstalk between cAMP signal pathway and Ras [56, 57], and SSMD may contribute to restore a new balance between them.

Moreover, we find that the results of GO and KEGG are complementary and interrelated. For example, in SS patients, both antigen and signal molecules can mediate cell communication in the form of exosomes [58]. Cortes-Troncoso et al. [59] found that mir-142-3p from T-cell exon was a key immunopathology factor in SS. The activated T cells secrete exosomes containing mir-142-3p and transfer them to glandular cells. Mir-142-3p can suppress the production of cAMP and other proteins in salivary gland cells through regulation of intracellular Ca2 ^+^ and other key elements. And the targets of SSMD predicated by GO and KEGG are enriched in exosomes and act on cAMP, which is consistent with the pathological mechanism of SS. In addition, GO and KEGG both revealed the regulation of SSMD on apoptosis [60]. To sum up, SSMD can act on multiple targets and participate in various biological processes, which can intervene SS from different pathological mechanisms.

### Immnue function of SSMD

**Fig. 3:**
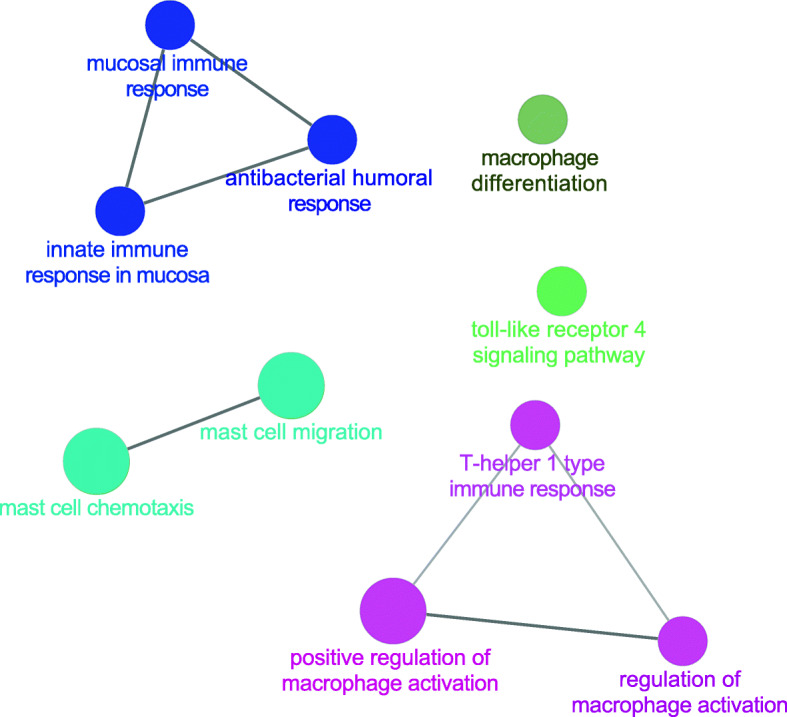
Immunomodulatory function of SSMD

We used the ClueGO plug-in of Cytoscape to study immune-related processes that SSMD involved in. As shown in Fig. 3., the results indicated that SSMD could regulate macrophage differentiation, toll-like receptor 4 (TLR4) signaling pathway, T-helper 1 (Th1) immune response, etc. Macrophages are derived from medullary monocytes or from resident cells in vitelline sac or fetal liver, which represent important components of the innate immune system and act as phagocytes to engulf foreign substances and play an important role in autoimmune diseases. Macrophages are reported to be one of the earliest infiltrates of salivary glands in NOD mice, arriving before dendritic cells, B and T lymphocytes [61]. In addition, the high expression of macrophage-derived molecules such as chitinase-3 protein 1 and chitinase-1 are associated with the increased severity of pSS lesions [62]. Aya Ushio et al. [63] found that the number of CD11blow macrophages were significantly increased in pSS mice, and the expression of scavenger receptors (CD36 and CD204) on CD11blow macrophages were significantly enhanced, and their phagocytic activity was up-regulated. Thus, macrophages may contribute to the onset of the disease. These studies all revealed the core role of macrophages in the pathogenesis of pSS, and our study speculated that one of the mechanisms by which SSMD treated SS was to regulate macrophage differentiation. However, the specific effect of SSMD on macrophage differentiation remains to be further explored.

TLRs belong to the pattern recognition receptor (PRRs) family, which are relatively conservative in evolution. At present, 10 different TLRs genotypes have been found in human body, which activate downstream by binding with different adapters and mediate innate immunity and autoimmune diseases. Current research has paid particular attention to TLRs located on cell surface, especially TLR4 and TLR2, which are over-expressed in salivary epithelial cells (SGEC), acinus cells and the invasive monocytes of the salivary glands in SS patients [64, 65]. TLR agonists stimulate CD54 expression and IL-6 production through phosphorylation of MAPKs in HSG cells [66], leading to chronic inflammation. Therefore, we hypothesized that SSMD could protect salivary gland tissue cells by interfering with the TLR4 signaling pathway.

T cells can be classified as cytotoxic T cells, helper T cells, regulatory/inhibitory T cells, etc. Th1 cells produce cytokines including IFN-γ and TNF-α. and activate macrophages, natural killer cells, and CD8^+^ T cells so as to regulate cell-mediated immune response. Various studies have reported that high level of Th1-releasing cytokines drive the progress of pSS [67]. For instance [68], IFN-γ has been proven to facilitate the entrance of inflammatory cells to glands by inducing the secretion of glandular adhesion molecules [69, 70]. IFN- γ knockout NOD mice showed delayed initiation and reduced severity of exostal inflammation [71]. TNF-α, another important cytokine secreted by Th1 cells, significantly increased in peripheral blood and salivary gland tissues of pSS [72]. Zhou et al. [73] found that neutralizing anti- TNF- α antibody at the initial stage of disease in female NOD mice could significantly improve secretion function of salivary gland and reduce the number of T and B cells in exocrine gland. The above studies revealed the role of Th1-cell-mediated pathology in pSS [74]. Moreover, our study predicted that SSMD may participate in the Th 1 immune response and help to alleviate the inflammatory damage of pSS immune system.

**Fig. 4:**
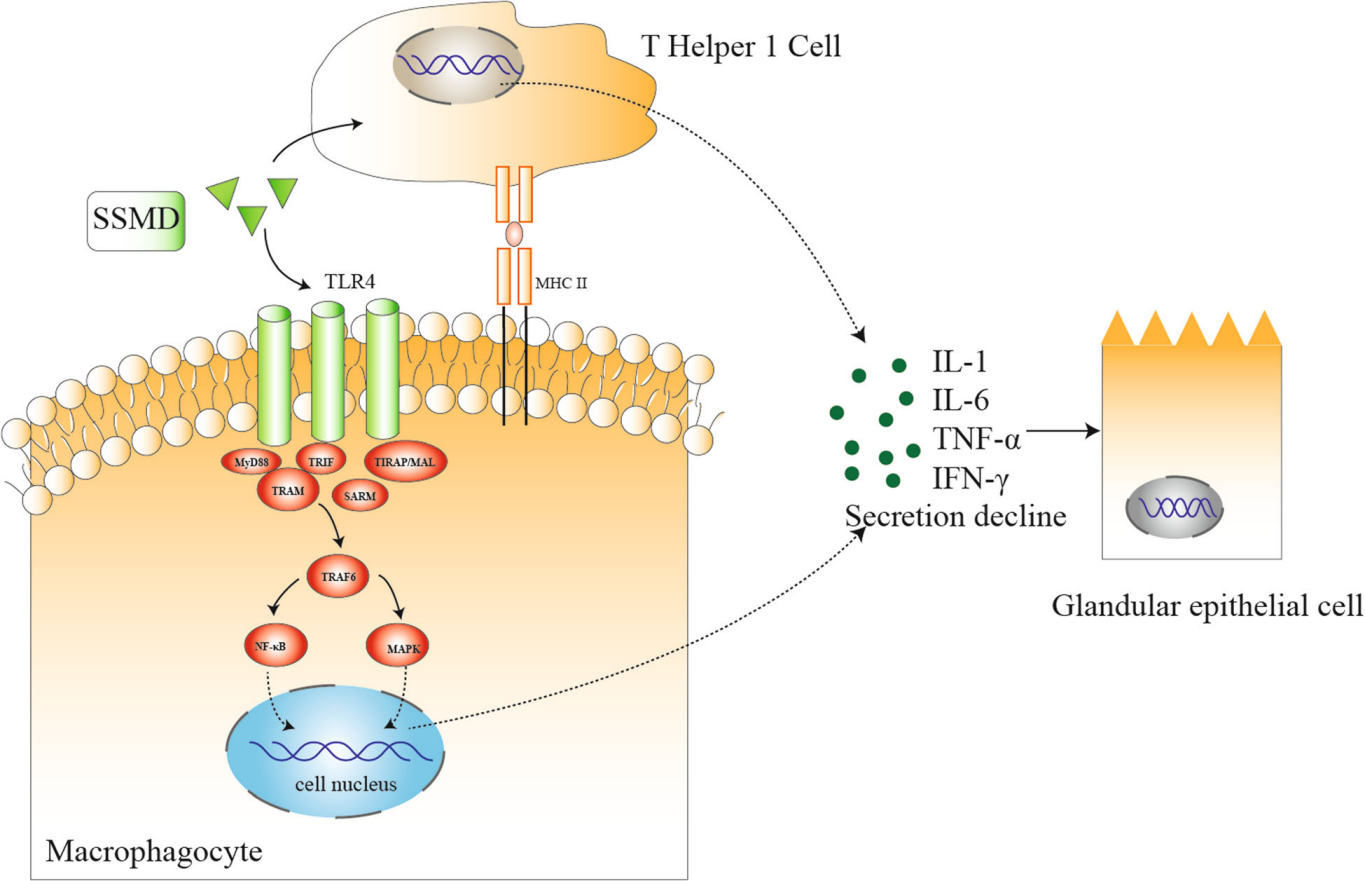
The immune mechanism of SSMD

In summary, macrophage differentiation, TLR4 signaling pathway and Th1 immune response, together with other immune elements constitute a complex network and play a non-negligible role in the pathogenesis of pSS (Fig. 4). We hypothesize that SSMD acts on the above immune processes, and is expected to break the pathological immune network in pSS, which provide a new strategy for the immunoregulatory treatment of pSS.

### The intersection targets of SSMD and pSS

**Fig. 5:**
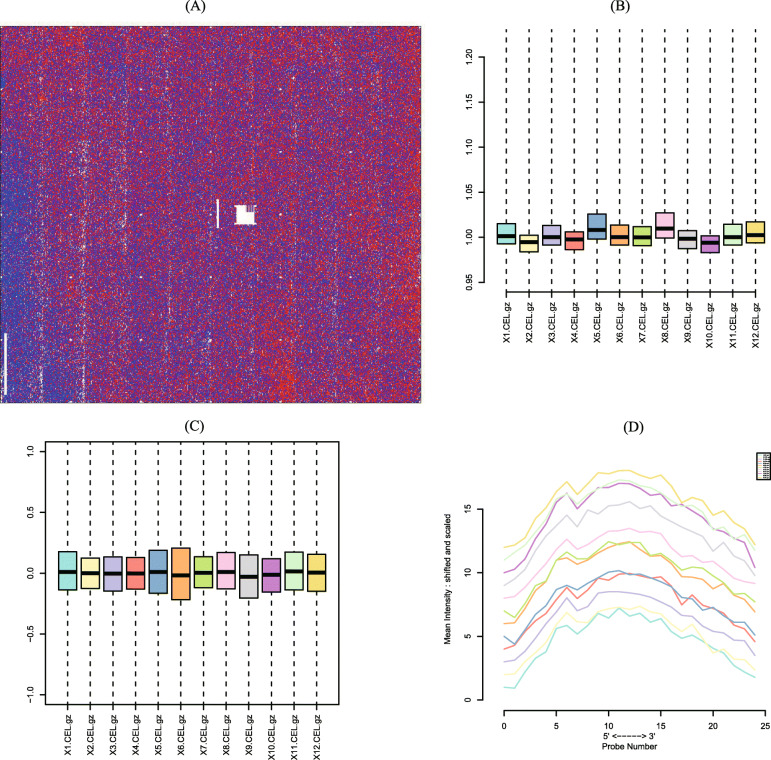
The quality control results of GSE97614. (A) Residual plot of the CEL files in GSE97614; (B) Nuse plost reflects the consistency of parallel experiments; (C) RLE plot reflects the consistency of parallel experiments; (D) RNA degradation plot

We obtained a microarray from the GEO database (GSE97614) [49, 75]. Quality control analysis proved the reliability of GSE97614 (Fig. 5). The Affymetrix microarray was used for transcriptome analysis of total RNA from non-tumor salivary gland epithelial cells (SGEC) strains in 3 non-SS-SICCA controls and 9 pSS patients. Microarray analysis was performed with the R statistical environment version 2.13 with Bioconductor package. Through GEO2R and conditional screening, 162 DEGs were finally obtained.

### Construction of protein PPI network

**Fig. 6:**
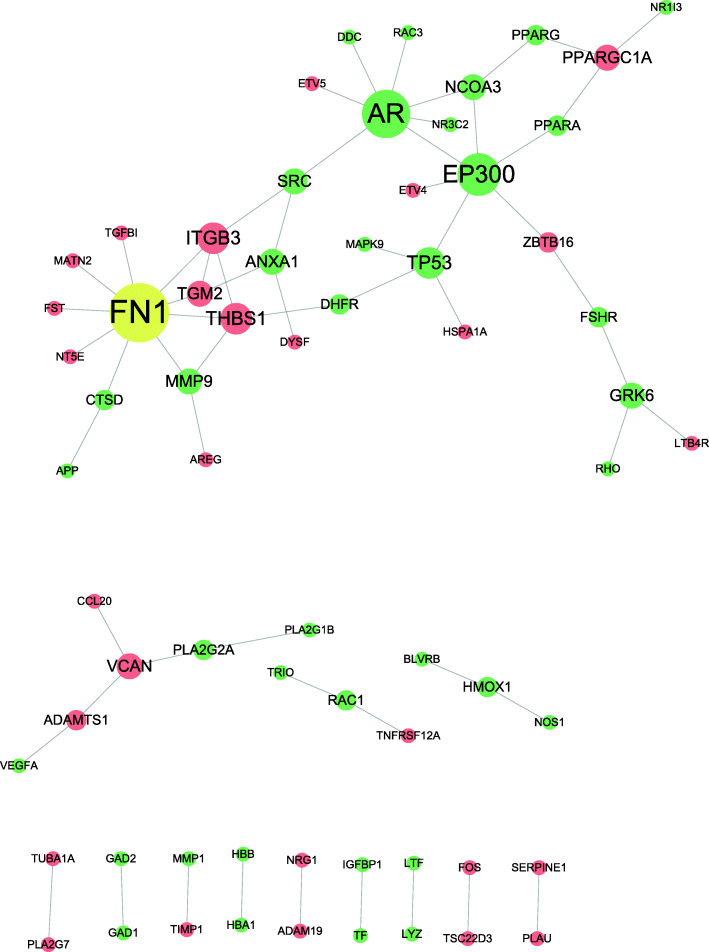
PPI network diagram. Green represents SSMD active protein, red represents pSS protein and yellow represents SSMD and pSS interaction protein

We made a PPI network for the 88 SSMD targets and 162 DEGs, as shown in Fig. 6. In the network diagram, we could find that there were one-to-one correspondence between 17 targets of SSMD and 19 DEGs, among which MMP9 and ANXA were respectively linked to 2 DEGs. In the figure, the targets of SSMD and disease targets were interconnected to form a complex network, indicating that SSMD could directly or indirectly act on multiple targets of pSS, thus playing an effective therapeutic role. Among them, fibronectin 1(FN1) was the co-acting targets of SSMD and pSS, and was interlinked with 9 disease proteins, which was located at the core position in the PPI network. Thus, SSMD could regulate multiple biological processes and play a core and extensive therapeutic role on pSS by acting on FN1. Studies have found that FN1 is widely involved in the process of cell migration, adhesion, proliferation and tissue repair. M. L. Anfimova [76] suggested that FN1 can be a clinical biomarker for pSS. Moreover, we found that FN1 interacted with transforming growth factor β1(TGF-β1) which acted as a major inhibitor of the immune system. TGF-β1 has been proved to stimulate the synthesis of extracellular matrix (ECM) such as FN1, collagen and proteoglycan, inhibit the production of stromal proteases, and promote the expression of various cell-junction protein receptors and the binding of ECM components to these receptors [77]. Furthermore, when TGF-β1^−^/^−^ mice were treated with synthetic fibronectin peptide, it could prevent leukocyte infiltration and reverse acinus and duct dysfunction [78]. Our study showed FN1 and TGF-β1 could be the hub targets of SSMD.

In addition, Matrix metalloproteinase − 9 (MMP-9) also interacts with FN1 in PPI network, which belongs to a family of zinc requiring neutropeptidase and is involved in the remodeling of ECM. Masumi Asatsuma et al. proved that the expression of MMP-9 was increased in the salivary glands of patients with severely active pSS [79], and the increased expression and activity of MMP-9 led to the destruction of basement membrane and salivary acinus structure of pSS [80, 81]. In addition, MMP-9 can stimulate the immune inflammatory response. Keiko Aota et al. believed that MMP-9 participated in the pathogenesis of pSS by promoting the production of CXCL10 in lesions induced by IFN-γ [82]. Therefore, the connection between MMP-9 and FN1 reflects that MMP-9 leads to pathological damage in pSS by breaking down FN, and aggravates the degree of disease through an independent pro-inflammatory mechanism. In a word, SSMD plays a protective role on tissues and organs of pSS by acting on FN1 and regulating its interaction with MMP9, thus affecting their various biological functions.

### Results of PCA

**Fig. 7:**
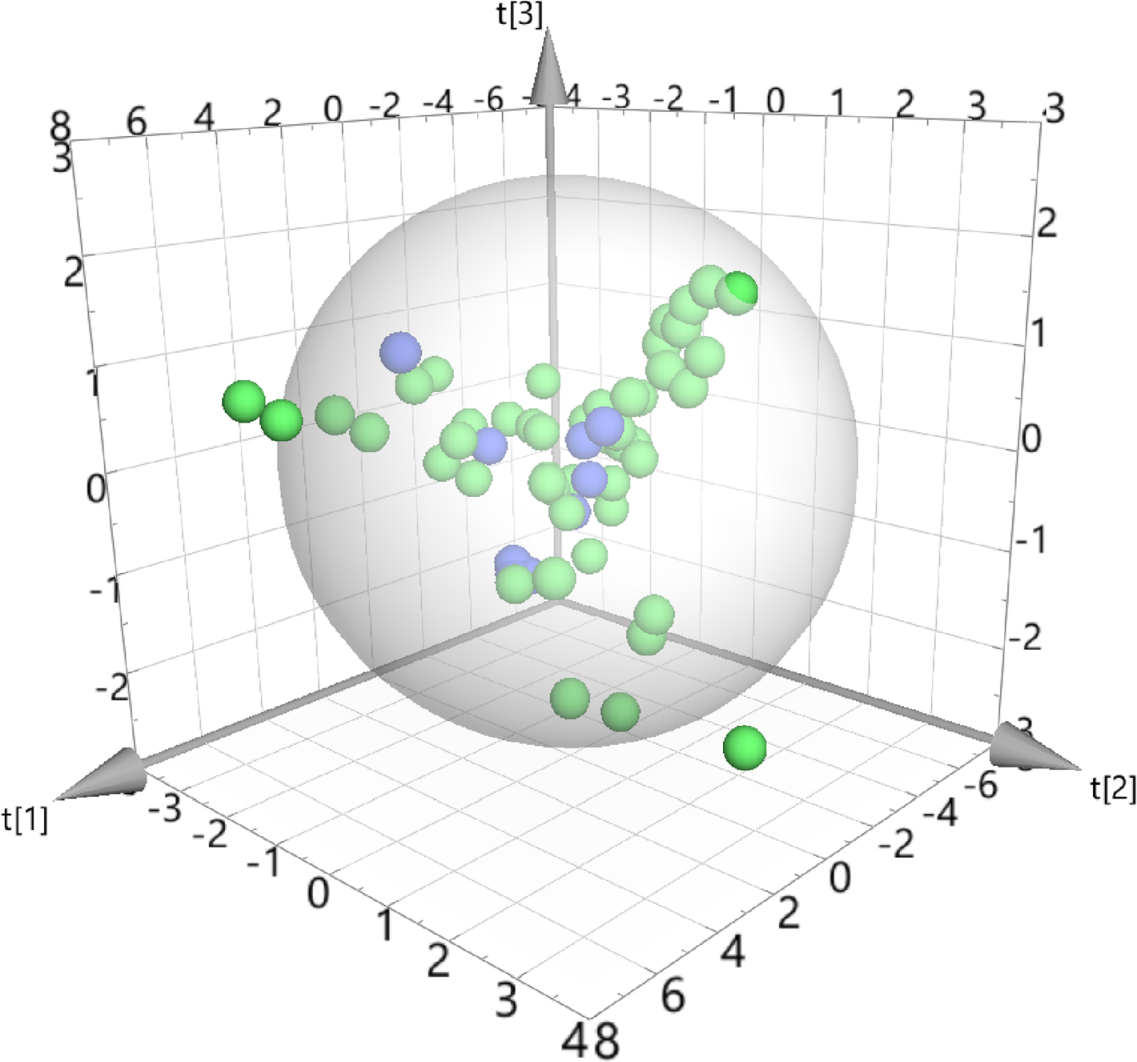
PCA diagram of SSMD active components and anti pSS drugs. Green represented the SSMD active ingredients and blue represented the FDA standard drugs

To further determine the feasible efficacy of SSMD in pSS, 10 standard drugs recommended by FDA for pSS derived from Drugbank (Table 4) as well as the SSMD active ingredients were collected to conduct PCA. The result was shown in Fig. 7., in which green represented the SSMD active ingredients and blue represented the FDA standard drugs. The green and blue spheres overlapped in spatial distribution to a great extent. Thus it can be preliminarily judged that there was little difference between the SSMD active ingredients and FDA-recommended drug for pSS. SSMD showed great potential in treating pSS.

**Table 4:** FDA standard drugs for pSS derived from Drugbank

NO.	Drug
1	Pilocarpine
2	Cevimeline
3	Hydroxychloroquine
4	Methotrexate
5	Azathioprine
6	Cyclosporin A
7	Cyclophosphamide
8	Methylprednisolone
9	Prednisolone
10	Cortisone

### Molecular docking analysis

**Fig. 8:**
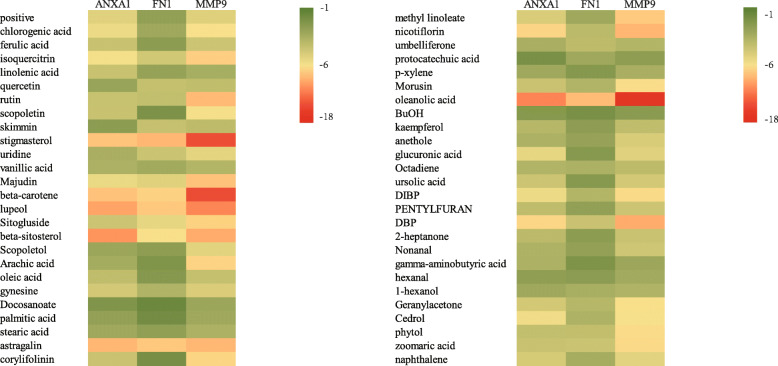
Molecular docking results of key pharmacodynamic components and targets of SSMD-pSS: heat map. In the figure, the ordinate is the effective component, and the positive represents the endogenous ligand in the corresponding protein complex crystal. The data in the small square in the figure is the binding energy value of the component and the target

**Fig. 9:**
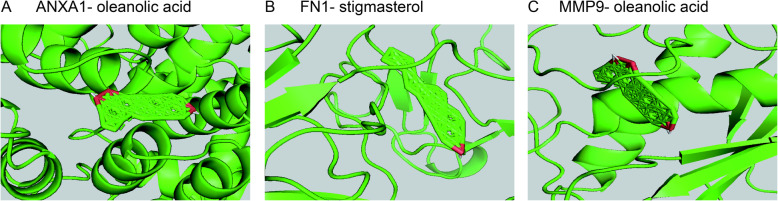
Molecular docking patterns of key components and targets of SSMD-pSS

To analyze the feasibility of SSMD in treating pSS, we also carried out molecular docking analysis on the active components of SSMD and the key targets of pSS. 2D structures of 51 components and the crystal structures and ligands of 3 targets were obtained. The PDB of ANXA1 crystal structure was 1HM6, and the ligand was SO4.The PDB of the crystal structure of FN1 was 3M7P, and the ligand was NAG. The PDB of MMP9 crystal structure is 1GKD, and the ligand was STN. We performed molecular docking for all pharmacodynamic components and targets in sequence and presented the results in a heat map (Fig. 8). The results showed that the binding energies of various active components in SSMD with the three target sites were all lower than their original ligands, including chloroquercitrin, Stigmasterol, Majudin, beta-carotene, Lupeol, beta-sitosterol, Astragalin, Nicotiflorin, Oleanolic acid, DIBP, DBP, Cedrol, etc. It indicated that the above active ingredients exerted good affinity with disease targets and played a central role in treatment. We showed the top 3 molecular docking diagrams with low binding energies in Fig. 9. These findings provide valuable information for the development of pSS drugs.

## Discussion

In this study, we systematically explored the potential molecular mechanism of SSMD by analyzing the active components, targets of SSMD through GO, KEGG enrichment and PPI network. Moreover, we verified the efficacy of SSMD for pSS by intersecting the target of SSMD and the DEGs of pSS. In order to reveal the potential therapeutic mechanism of this decoction, we collected the components of SSMD using TCMSP and TCMID databases and totally obtained 1060 active ingredients. Among them, 76 components were found in more than one herb, and these components shared by different herbs may be key components of the biological function of SSMD. Through literature research, we found that these drugs showed a variety of pharmacological characteristics, such as antioxygenation, anti-oxidative stress, anti-fibrosis, and exerted a regulation effccts on immune activity, which were consistent with the pathological mechanism of autoimmune diseases, especially pSS [21]. When analyzing the targets of SSMD, the database of pharmmapper and STITCH were utilized, and 88 targets were obtained, which were closely associated with 292 effective compounds in SSMD. Among them, PMP2, AR and GAD1 were closely related with the active components of SSMD, and were also closely associated with the pathological injury mechanisms of lacrimal gland, salivary gland, nervous system injury, etc. in pSS.

In order to understand the specific regulation mechanism of SSMD systematically, 88 targets of SSMD were put into the DAVID database for GO and KEGG analysis, and the advantages of multiple-pathway treatment by SSMD were revealed. GO analysis showed that 88 targets were widely distributed, both intracellular and extracellular, and could be involved in a variety of biological processes, such as REDOX process, transcriptional regulation, negative regulation of apoptosis process and drug response process. In addition, KEGG analysis revealed that SSMD can affect various signaling pathways that can be divided into the following four types, including human diseases, environmental information processing, metabolic pathways (amino acid and fat metabolism, steroid hormone metabolism and drug metabolism) and signal transduction (Ras signaling pathway and cAMP signaling pathway). Moreover, by putting the result of GO and KEGG together, we found that the results of these two parts were complementary and interrelated, which was helpful for us to acquire a more complete understanding of SSMD. To sum up, targets of SSMD are widely distributed with diverse pathways of action, and SSMD can treat pSS by a number of different pathological mechanisms.

Since pSS is a disease characterized by autoimmune destruction, an in-depth study of the targets related to immune function of SSMD is necessary to help us deeply understand the immune regulation mechanism of drugs, uncover the mystery of TCM, and find objective evidence to make up for the deficiency of current immunotherapy programs and provide novel and effective methods. Therefore, we applied the ClueGO plug-in in Cytoscape to study the biological process of related genes. The results showed that SSMD regulated the body’s immune function by influencing the following immune processes, including macrophage differentiation, TLR4 signaling pathway, immune response of Th1 cells, etc. These immunomodulatory mechanisms are closely related to the occurrence and development of pSS, so we hypothesized that SSMD may affect the above pathways to regulate the immune and inflammatory states of pSS itself. In order to intuitively understand the regulation of SSMD on SS genes. PPI network was constructed between the target of SSMD and DEGs of pSS in gene chip GSE97614 which was obtained from GEO database. We found that FN1 located in the core position,connected with multiple disease targets of pSS, such as TGF-β1 and MMP-9, etc. and became the key hub of SSMD acting on multiple pathological mechanisms of pSS.

In order to further analyze the feasibility of SSMD in treating pSS, we conducted PCA and found that there was little difference between the active components of SSMD and the standard drugs of pSS, which indirectly proved the potential value of SSMD in the treatment of pSS. Then we carried out molecular docking and found that many active components showed high affinity with different targets of pSS. The drug molecules of SSMD can combine with target molecules one after another, resulting in a superposition effect. When the target is fully occupied, the efficacy starts to show, and the superposition effect of different active ingredients, in terms of concentration and action time, ensures the lasting efficacy. Through the above analysis, we found that a single chemical component can combine with multiple targets, and different chemical components can bind to the same target molecule, reflecting the function characteristics of SSMD through multiple components and multiple targets.

## Conclusion

In this study, by integrating bioinformatics and network pharmacology, we analyze the main bioactive components and pharmacological mechanism of SSMD for the treatment of pSS. The application of network pharmacology lays a foundation for further research on the mechanism of SSMD, provides theoretical basis for systematic experimental research and clinical application of SSMD in treating pSS, and inspires new research ideas and methods for the study of TCM compound therapy of pSS and its related drug targets. In addition, principal component analysis and molecular docking were used to further demonstrate the feasibility of SSMD in the treatment of pSS. However, the active components and targets of some TCM have not been fully identified, which needs to be further confirmed by experiments. In addition, the therapeutic targets related to pSS are still being updated, so we should continue to focus on the development so as to provide new, effective and safe therapeutic strategies for pSS patients.

## Supplementary Information

**Additional file 1: Table S1.** The detailed information of herbs and compounds in SSMD.

**Additional file 2: Table S2.** Relationship between effective compounds and corresponding targets of SSMD.

## Data Availability

The datasets used and/or analysed during the current study available from the corresponding author on reasonable request.

## Abbreviations

AR: Androgen receptor
DEGs: Differentially expressed genes
FABPs: Fatty acid binding proteins
FN1: Fibronectin 1
GAD1: Glutamic acid decarboxylase 1
MD: Molecular docking
PCA: Principal component analysis
PMP2: Peripheral myelin protein 2
PPI: Protein-protein interaction
pSS: Primary Sjogren’s Syndrome
SSMD: Sha-Shen-Mai-Dong decoction
TLR4: Toll-like receptor 4
