# Supplementary material for: Deciphering potential pharmacological mechanism of Sha-Shen-Mai-Dong decoction on primary Sjogren’s syndrome
Source: BMC Complement Med Ther. 2021 Mar 1;21:79. doi: 10.1186/s12906-021-03257-7 (PMC7923330; doi:10.1186/s12906-021-03257-7)
Supplement: Supplementary file 1 — Additional file 1: Table S1. The detailed information of herbs and compounds in SSMD. [file 12906_2021_3257_MOESM1_ESM.doc]

**Supplement Table S1. The detailed information of herbs and compounds in SSMD**

| Herb | Component |
| --- | --- |
| Glehnia littoralis | (2s,6 ζ)-3,7-dimethyloct-3(10)-ene-1,2,6,7-tetrol1-o-β-d-glucopyranoside |
| Glehnia littoralis | (3r ')-hydroxymarmesin 4'-o-β-d-gluco-pyranoside |
| Glehnia littoralis | (3r)-2-hydroxymethylbutane-1,2,3,4-tetrol |
| Glehnia littoralis | (4r)-p-menth-1-ene-7,8-diol 8-o-β-d-glucopyra-noside |
| Glehnia littoralis | (8E)heptadecyl-1,8-diene-4,6-diene-3,10-diol |
| Glehnia littoralis | (?)-secoisolariciresinol |
| Glehnia littoralis | (?)-secoisolariciresinol4-o-β-d-glucopyranoside |
| Glehnia littoralis | (d)-threitol |
| Glehnia littoralis | 1-deoxy-d-lyxitol |
| Glehnia littoralis | 2-deoxy-d-ribitol1 |
| Glehnia littoralis | 3,7-dimethyloct-3(10)-ene-1,2,6,7-tetrol |
| Glehnia littoralis | 4''-hydroxyimperatorin 4''-o-β-d-glucopyra-noside |
| Glehnia littoralis | 4-[β-d-apiofuranosyl-(1→6)-β-d-glucopyra-nosyloxy]-3-methoxypropiophenone |
| Glehnia littoralis | adeninenucleoside |
| Glehnia littoralis | adenosine |
| Glehnia littoralis | baihuaqianhuoside |
| Glehnia littoralis | benzyl alcoholo-β-d-glucopyranoside |
| Glehnia littoralis | bergamotin |
| Glehnia littoralis | bergaptol-o-β-d-glucopyranoside |
| Glehnia littoralis | butane-2,3-diol 2-o-β-d-glucopyranoside |
| Glehnia littoralis | Butanol α-D- fructofuranoside |
| Glehnia littoralis | caffeic acid |
| Glehnia littoralis | caffeicacid |
| Glehnia littoralis | chlorogenic acid |
| Glehnia littoralis | chlorogenicacid |
| Glehnia littoralis | cis-falcarindiol |
| Glehnia littoralis | cis-p-menth-2-ene-1α,7,8-triol |
| Glehnia littoralis | citrusin a |
| Glehnia littoralis | Cool in the bergamot |
| Glehnia littoralis | corchoionoside a |
| Glehnia littoralis | crenulatin |
| Glehnia littoralis | Do isoimperatorin lactone |
| Glehnia littoralis | erythritol |
| Glehnia littoralis | ethylβ-d-glucopyranoside |
| Glehnia littoralis | Fa Kalin diol |
| Glehnia littoralis | ferulic acid |
| Glehnia littoralis | ferulicacid |
| Glehnia littoralis | glehlinoside a |
| Glehnia littoralis | glehlinoside b |
| Glehnia littoralis | glehlinoside c |
| Glehnia littoralis | icariside f2 |
| Glehnia littoralis | imperatorin |
| Glehnia littoralis | isobutyl β-d-glucopyranoside |
| Glehnia littoralis | isoimperatorin |
| Glehnia littoralis | Isopropyl apioglucoside |
| Glehnia littoralis | isopropylβ-d-glucopyranoside |
| Glehnia littoralis | isoquercitrin |
| Glehnia littoralis | junipediol a 29-o-β-d-glucopyranoside |
| Glehnia littoralis | linolenic acid |
| Glehnia littoralis | marmesinin |
| Glehnia littoralis | osthenol-7-o-beta-gentiobioside |
| Glehnia littoralis | osthenol-7-o-β-gentiobioside |
| Glehnia littoralis | oxymarmesinin 5'-o-β-d-glucopyranoside |
| Glehnia littoralis | panaxynol |
| Glehnia littoralis | pehllopterin |
| Glehnia littoralis | phenethylβ-d-glucopyranoside |
| Glehnia littoralis | picraquassioside a |
| Glehnia littoralis | psoralen |
| Glehnia littoralis | psoralenol |
| Glehnia littoralis | quercetin |
| Glehnia littoralis | rutin |
| Glehnia littoralis | salicylic acid |
| Glehnia littoralis | scopoletin |
| Glehnia littoralis | skimmin |
| Glehnia littoralis | stigmasterol |
| Glehnia littoralis | Stigmasterol 3-O-β-D- glucopyranoside |
| Glehnia littoralis | syringin |
| Glehnia littoralis | Trans, trans-2,4-decadienal |
| Glehnia littoralis | Trans-2-octene-1-ol |
| Glehnia littoralis | trans-p-menth-2-ene-1α,7,8-triol |
| Glehnia littoralis | trans-p-menthane-1α,2β,8-triol |
| Glehnia littoralis | uridine |
| Glehnia littoralis | vanillicacid |
| Glehnia littoralis | Vanilloid |
| Glehnia littoralis | Vanilloid 4-O-β-D- pyranoside |
| Glehnia littoralis | xanthotoxin |
| Glehnia littoralis | xanthotoxol |
| Glehnia littoralis | xanthotoxol 8-o-β-d-glucopyranoside |
| Glehnia littoralis | vanillic acid |
| Glehnia littoralis | EIC |
| Glehnia littoralis | Trochol |
| Glehnia littoralis | Secoisolariciresinol |
| Glehnia littoralis | Alloisoimperatorin |
| Glehnia littoralis | Falcarindiol |
| Glehnia littoralis | Ammidin |
| Glehnia littoralis | lecithin |
| Glehnia littoralis | Marmesin |
| Glehnia littoralis | Majudin |
| Glehnia littoralis | Ammijin |
| Glehnia littoralis | n-butyl-β-D-fructoufranoside |
| Glehnia littoralis | Nootkatin |
| Glehnia littoralis | Bergaptin |
| Glehnia littoralis | Sinapyl alcohol |
| Glehnia littoralis | Uvadex |
| Glehnia littoralis | Heriguard |
| Glehnia littoralis | Cnidilin |
| Glehnia littoralis | Syrigin |
| Glehnia littoralis | lupeol |
| Glehnia littoralis | Sitogluside |
| Glehnia littoralis | beta-sitosterol |
| Glehnia littoralis | FER |
| Glehnia littoralis | Scopoletol |
| Glehnia littoralis | coumarin |
| Glehnia littoralis | Hirsutrin |
| Lablab niger | (?)-phaseollidin |
| Lablab niger | alpha-carotene |
| Lablab niger | Asparaginase |
| Lablab niger | beta-carotene |
| Lablab niger | D(+)-Sucrose |
| Lablab niger | d-alpha-pipecoline |
| Lablab niger | l-pipecilic acid |
| Lablab niger | phosphatidyl ethanolamine |
| Lablab niger | phytoagglutinin |
| Lablab niger | raffinose |
| Lablab niger | Rutinum |
| Lablab niger | stachyose |
| Lablab niger | stigmasterol |
| Lablab niger | stigmasterol-beta-d-glucoside |
| Lablab niger | Tyrosinase |
| Lablab niger | Xanthophyll |
| Lablab niger | zeatin |
| Lablab niger | zeaxanthine |
| Lablab niger | Arachic acid |
| Lablab niger | D-Homoproline |
| Lablab niger | oleic acid |
| Lablab niger | EIC |
| Lablab niger | CPI |
| Lablab niger | gynesine |
| Lablab niger | Docosanoate |
| Lablab niger | nicotinic acid |
| Lablab niger | palmitic acid |
| Lablab niger | stearic acid |
| Glycyrrhiza uralensis | (e)-1-[2,4-dihydroxy-3-(3-methyl-2-butenyl)phenyl]-3-(2,2-dimethyl-8-hydroxy-2h-benzo-pyran-6-yl)-2-propen-1-one |
| Glycyrrhiza uralensis | (e)-1-[2,4-dihydroxy-3-(3-methyl-2-butenyl)phenyl]-3-(4-hydroxy-3-[3-methyl-2-butenyl)phenyl]-2-propen-1-one |
| Glycyrrhiza uralensis | 18alpha-glycyrrhetinic acid |
| Glycyrrhiza uralensis | 18beta-glycyrrhetinic acid |
| Glycyrrhiza uralensis | 2,4,4'-trihydroxychalcone |
| Glycyrrhiza uralensis | 2,5-dihydroxymethyl-3,4-dihydroxypyrrolidine |
| Glycyrrhiza uralensis | 2-methyl-1,3,6-trihydroxyanthraquinone |
| Glycyrrhiza uralensis | 3'-(γ,γ-dimethylallyl)-kievitone |
| Glycyrrhiza uralensis | 3'-methoxyglabridin |
| Glycyrrhiza uralensis | 3,3'-dimethylquercetin |
| Glycyrrhiza uralensis | 3,4-dicaffeoyl-5-(3-hydroxy-3-methyl)glutaroyl quinic acid |
| Glycyrrhiza uralensis | 3-hydroxyglabrol |
| Glycyrrhiza uralensis | 3-hydroxyglabrol(ii) |
| Glycyrrhiza uralensis | 3-methyl-6,7,8-trihydropyrrolo[1,2-a]pyrimidin-2-one |
| Glycyrrhiza uralensis | 3-o-acetyl-glycyrrhetinicacid |
| Glycyrrhiza uralensis | 3-o-[β-d-glucuronopyranosyl-(1→2)-o-β-d-glucuronopyranosyl]-24-hydroxyglabrolide |
| Glycyrrhiza uralensis | 4'-o-methylglabridin |
| Glycyrrhiza uralensis | 5,6,7,8-tetrahydro-2, 4-dimethylquinoline |
| Glycyrrhiza uralensis | 5,6,7,8-tetrahydro-4-methylquinoline |
| Glycyrrhiza uralensis | 6,8-bis(c-β-glucosyl)-apigenin |
| Glycyrrhiza uralensis | 8-methoxy-5-o-glucoside flavone |
| Glycyrrhiza uralensis | 8-methyl-10-hydroxylycoctonine |
| Glycyrrhiza uralensis | alpha-trihydroxy coprostanic acid |
| Glycyrrhiza uralensis | astragalin |
| Glycyrrhiza uralensis | berniarin |
| Glycyrrhiza uralensis | corylifolinin |
| Glycyrrhiza uralensis | dibutyl uralsaponin a ester |
| Glycyrrhiza uralensis | dimethyl sebacate |
| Glycyrrhiza uralensis | ethyl-n-buthy-uralsaponin a esters |
| Glycyrrhiza uralensis | ferulic acid |
| Glycyrrhiza uralensis | formononetin |
| Glycyrrhiza uralensis | formononetin-7-glucoside |
| Glycyrrhiza uralensis | gamma-sitosterol |
| Glycyrrhiza uralensis | gancaonin a |
| Glycyrrhiza uralensis | gancaonin b |
| Glycyrrhiza uralensis | gancaonin c |
| Glycyrrhiza uralensis | gancaonin d |
| Glycyrrhiza uralensis | gancaonin e |
| Glycyrrhiza uralensis | gancaonin f |
| Glycyrrhiza uralensis | gancaonin i |
| Glycyrrhiza uralensis | gancaonin p-3'-methylether |
| Glycyrrhiza uralensis | gancaonin x |
| Glycyrrhiza uralensis | ganoderic acid a |
| Glycyrrhiza uralensis | glabrolide |
| Glycyrrhiza uralensis | glisoflavanone |
| Glycyrrhiza uralensis | gloeosteretriol |
| Glycyrrhiza uralensis | glyarallin b |
| Glycyrrhiza uralensis | glycycoumarin |
| Glycyrrhiza uralensis | glycyphyllin |
| Glycyrrhiza uralensis | glycyrin |
| Glycyrrhiza uralensis | glycyrol |
| Glycyrrhiza uralensis | glycyroside |
| Glycyrrhiza uralensis | glycyrrhetinic acid |
| Glycyrrhiza uralensis | glycyrrhetinicacid |
| Glycyrrhiza uralensis | glycyrrhetol |
| Glycyrrhiza uralensis | glycyrrhisoflavanone |
| Glycyrrhiza uralensis | glycyrrhisoflavone |
| Glycyrrhiza uralensis | glycyrrhiza-flavonol a |
| Glycyrrhiza uralensis | glycyrrhizic acid |
| Glycyrrhiza uralensis | glycyrrhizicacid |
| Glycyrrhiza uralensis | glycyrrhizin |
| Glycyrrhiza uralensis | glyeurysaponin |
| Glycyrrhiza uralensis | glyurallin a |
| Glycyrrhiza uralensis | glyuranolide |
| Glycyrrhiza uralensis | glyyunnanprosapogenin d |
| Glycyrrhiza uralensis | glyzaglabrin |
| Glycyrrhiza uralensis | gmelofuran |
| Glycyrrhiza uralensis | hispaglabridin a |
| Glycyrrhiza uralensis | hispaglabridin b |
| Glycyrrhiza uralensis | hispidulin |
| Glycyrrhiza uralensis | isoglycyrol |
| Glycyrrhiza uralensis | isogosferol |
| Glycyrrhiza uralensis | isolicoflavonol |
| Glycyrrhiza uralensis | isoliensinine |
| Glycyrrhiza uralensis | isoliquiriligenin |
| Glycyrrhiza uralensis | isoliquiritigenin |
| Glycyrrhiza uralensis | isoliquiritin |
| Glycyrrhiza uralensis | isolobelanine |
| Glycyrrhiza uralensis | isoononin |
| Glycyrrhiza uralensis | isoorientin |
| Glycyrrhiza uralensis | isoquercitrin |
| Glycyrrhiza uralensis | isoramanone |
| Glycyrrhiza uralensis | isoschaftoside |
| Glycyrrhiza uralensis | isotrifoliol |
| Glycyrrhiza uralensis | isotrilobine |
| Glycyrrhiza uralensis | kanzonol k |
| Glycyrrhiza uralensis | kanzonol l |
| Glycyrrhiza uralensis | lensinine |
| Glycyrrhiza uralensis | licobenzofuran |
| Glycyrrhiza uralensis | licobichalcone |
| Glycyrrhiza uralensis | licochalcone a |
| Glycyrrhiza uralensis | licocoumarone |
| Glycyrrhiza uralensis | licoflavone |
| Glycyrrhiza uralensis | licofuranocoumarin |
| Glycyrrhiza uralensis | licoisoflavaone |
| Glycyrrhiza uralensis | licoisoflavone |
| Glycyrrhiza uralensis | licoleafol |
| Glycyrrhiza uralensis | licopyranocoumarin |
| Glycyrrhiza uralensis | licoricesaponin a3 |
| Glycyrrhiza uralensis | licoricesaponin b2 |
| Glycyrrhiza uralensis | licoricesaponin c2 |
| Glycyrrhiza uralensis | licoricesaponin d3 |
| Glycyrrhiza uralensis | licoricesaponin e2 |
| Glycyrrhiza uralensis | licoricesaponin f3 |
| Glycyrrhiza uralensis | licoricesaponin g2 |
| Glycyrrhiza uralensis | licoricesaponin j2 |
| Glycyrrhiza uralensis | licoricesaponin k2 |
| Glycyrrhiza uralensis | licoricesaponine a3 |
| Glycyrrhiza uralensis | licoricesaponine c2 |
| Glycyrrhiza uralensis | licoricesaponine d3 |
| Glycyrrhiza uralensis | licoricesaponine f3 |
| Glycyrrhiza uralensis | licoricesaponine g2 |
| Glycyrrhiza uralensis | licoricesaponine h2 |
| Glycyrrhiza uralensis | licoricesaponine j2 |
| Glycyrrhiza uralensis | licoricesaponine k2 |
| Glycyrrhiza uralensis | licoricesaponinh2 |
| Glycyrrhiza uralensis | licoricidin |
| Glycyrrhiza uralensis | licoricone |
| Glycyrrhiza uralensis | licorisoflavan a |
| Glycyrrhiza uralensis | liquiriligenin |
| Glycyrrhiza uralensis | liquiritigenin |
| Glycyrrhiza uralensis | liquiritigenin-7,4'-diglucoside |
| Glycyrrhiza uralensis | liquiritigenin-7-o-beta-d-(3-o-acetyl)-apiofuranosyl-4'-o-beta-d-glucopyranoside |
| Glycyrrhiza uralensis | liquiritigenin4'-o-β-d-apio-d-furanosyl(1→2)-β-d-glucopyranoside |
| Glycyrrhiza uralensis | liquiritin |
| Glycyrrhiza uralensis | liquoric acid |
| Glycyrrhiza uralensis | lupiwighteone |
| Glycyrrhiza uralensis | methyl 18α-hydroxyglycyrrhetate |
| Glycyrrhiza uralensis | methyl 2-hydroxy-3,4-dimethoxy benzoate |
| Glycyrrhiza uralensis | methyl 3-o-beta-d-glucopyranosyl polygalacate |
| Glycyrrhiza uralensis | methyl linoleate |
| Glycyrrhiza uralensis | methyl-24-hydroxy-11-deoxoglycyrrhetate |
| Glycyrrhiza uralensis | methyl-24-hydroxyglycyrrhetate |
| Glycyrrhiza uralensis | methyl-n-butyl-uralsaponin a esters |
| Glycyrrhiza uralensis | methylglycyrrhetate |
| Glycyrrhiza uralensis | methylglyoxal |
| Glycyrrhiza uralensis | monoammonium glycyrrhizinate |
| Glycyrrhiza uralensis | n-tricosane |
| Glycyrrhiza uralensis | narcissin |
| Glycyrrhiza uralensis | narwedine |
| Glycyrrhiza uralensis | neohancoside a |
| Glycyrrhiza uralensis | neoisoliquiritin |
| Glycyrrhiza uralensis | neoisopulegol |
| Glycyrrhiza uralensis | neoliquiritin |
| Glycyrrhiza uralensis | neomatatabiol |
| Glycyrrhiza uralensis | neouralenol |
| Glycyrrhiza uralensis | neowilforine |
| Glycyrrhiza uralensis | nicotiflorin |
| Glycyrrhiza uralensis | ononin |
| Glycyrrhiza uralensis | ononitol |
| Glycyrrhiza uralensis | phaseollinisoflavan |
| Glycyrrhiza uralensis | phebalosin |
| Glycyrrhiza uralensis | rutin |
| Glycyrrhiza uralensis | ruvoside |
| Glycyrrhiza uralensis | schaftoside |
| Glycyrrhiza uralensis | sigmoidin b |
| Glycyrrhiza uralensis | sinapic acid |
| Glycyrrhiza uralensis | tetrahydroharmine |
| Glycyrrhiza uralensis | tetrahydropalmatine |
| Glycyrrhiza uralensis | umbelliferone |
| Glycyrrhiza uralensis | uralene |
| Glycyrrhiza uralensis | uralenin |
| Glycyrrhiza uralensis | uralenneoside |
| Glycyrrhiza uralensis | uralenol |
| Glycyrrhiza uralensis | uralenol-3-methylether |
| Glycyrrhiza uralensis | uralsaponin a |
| Glycyrrhiza uralensis | uralsaponin b |
| Glycyrrhiza uralensis | uralstilbene |
| Glycyrrhiza uralensis | urea |
| Glycyrrhiza uralensis | vicianin |
| Glycyrrhiza uralensis | β-sitosterol |
| Glycyrrhiza uralensis | protocatechuic acid |
| Glycyrrhiza uralensis | o-xylene |
| Glycyrrhiza uralensis | m-xylene |
| Glycyrrhiza uralensis | p-xylene |
| Glycyrrhiza uralensis | (L)-alpha-Terpineol |
| Glycyrrhiza uralensis | Arachic acid |
| Glycyrrhiza uralensis | Inermine |
| Glycyrrhiza uralensis | Vicenin-2 |
| Glycyrrhiza uralensis | α-cubebol |
| Glycyrrhiza uralensis | Morusin |
| Glycyrrhiza uralensis | ICO |
| Glycyrrhiza uralensis | DFV |
| Glycyrrhiza uralensis | Izoforon |
| Glycyrrhiza uralensis | Mairin |
| Glycyrrhiza uralensis | OCT |
| Glycyrrhiza uralensis | ISOHEPTANE |
| Glycyrrhiza uralensis | Heptan |
| Glycyrrhiza uralensis | Jaranol |
| Glycyrrhiza uralensis | 21987_FLUKA |
| Glycyrrhiza uralensis | Medicarpin |
| Glycyrrhiza uralensis | oleanolic acid |
| Glycyrrhiza uralensis | EB |
| Glycyrrhiza uralensis | Pinocembrin |
| Glycyrrhiza uralensis | butylated hydroxytoluene |
| Glycyrrhiza uralensis | BuOH |
| Glycyrrhiza uralensis | isorhamnetin |
| Glycyrrhiza uralensis | sitosterol |
| Glycyrrhiza uralensis | 7,4'-Dihydroxyflavone |
| Glycyrrhiza uralensis | Narcissoside |
| Glycyrrhiza uralensis | 7-Methoxy-2-methyl isoflavone |
| Glycyrrhiza uralensis | 2-Caren-10-al |
| Glycyrrhiza uralensis | Scopoletol |
| Glycyrrhiza uralensis | Calycosin |
| Glycyrrhiza uralensis | kaempferol |
| Glycyrrhiza uralensis | naringenin |
| Glycyrrhiza uralensis | Hirsutrin |
| Glycyrrhiza uralensis | 8-Prenylwighteone |
| Glycyrrhiza uralensis | Methylheptane |
| Glycyrrhiza uralensis | Castanin |
| Glycyrrhiza uralensis | beta-Terpinene |
| Glycyrrhiza uralensis | anethole |
| Glycyrrhiza uralensis | 2',7-Dihydroxy-4'-methoxyisoflavan-7-O-β-d-glucopyranoside |
| Glycyrrhiza uralensis | (E)-1-butoxyhex-2-ene |
| Glycyrrhiza uralensis | (2S)-2-[4-hydroxy-3-(3-methylbut-2-enyl)phenyl]-8,8-dimethyl-2,3-dihydropyrano[2,3-f]chromen-4-one |
| Glycyrrhiza uralensis | euchrenone |
| Glycyrrhiza uralensis | glucuronic acid |
| Glycyrrhiza uralensis | glyasperin B |
| Glycyrrhiza uralensis | glyasperin E |
| Glycyrrhiza uralensis | glyasperin F |
| Glycyrrhiza uralensis | Glyasperin C |
| Glycyrrhiza uralensis | glyasperins D |
| Glycyrrhiza uralensis | glyasperins Z |
| Glycyrrhiza uralensis | (E)-1-(2,4-dihydroxyphenyl)-3-(2,2-dimethylchromen-6-yl)prop-2-en-1-one |
| Glycyrrhiza uralensis | (2R)-1-[2,4-dihydroxy-5-(3-methylbut-2-enyl)phenyl]-2-hydroxy-3-[4-hydroxy-3-(3-methylbut-2-enyl)phenyl]propan-1-one |
| Glycyrrhiza uralensis | kanzonols K |
| Glycyrrhiza uralensis | kanzonols L |
| Glycyrrhiza uralensis | kanzonols T |
| Glycyrrhiza uralensis | kanzonols W |
| Glycyrrhiza uralensis | kanzonols X |
| Glycyrrhiza uralensis | (E)-1-(2,4-dihydroxyphenyl)-3-[4-hydroxy-3-(3-methylbut-2-enyl)phenyl]prop-2-en-1-one |
| Glycyrrhiza uralensis | licoagropin |
| Glycyrrhiza uralensis | (2S)-6-(2,4-dihydroxyphenyl)-2-(2-hydroxypropan-2-yl)-4-methoxy-2,3-dihydrofuro[3,2-g]chromen-7-one |
| Glycyrrhiza uralensis | glyinflanin A |
| Glycyrrhiza uralensis | naringin |
| Glycyrrhiza uralensis | Semilicoisoflavone B |
| Glycyrrhiza uralensis | Glepidotin A |
| Glycyrrhiza uralensis | Glepidotin B |
| Glycyrrhiza uralensis | Octadiene |
| Glycyrrhiza uralensis | (E)-1-[2,4-dihydroxy-3-(3-methylbut-2-enyl)phenyl]-3-[4-hydroxy-3-(3-methylbut-2-enyl)phenyl]prop-2-en-1-one |
| Glycyrrhiza uralensis | WLN: 4OVR |
| Glycyrrhiza uralensis | Phaseolinisoflavan |
| Glycyrrhiza uralensis | 3-(2-hydroxy-4-methoxyphenyl)-2H-chromen-7-ol |
| Glycyrrhiza uralensis | Glypallichalcone |
| Glycyrrhiza uralensis | echinatin |
| Glycyrrhiza uralensis | Karenzu DK2 |
| Glycyrrhiza uralensis | 8-(6-hydroxy-2-benzofuranyl)-2,2-dimethyl-5-chromenol |
| Glycyrrhiza uralensis | (1S,2S)-1,2-dimethylcyclopentane |
| Glycyrrhiza uralensis | Liconeolignan |
| Glycyrrhiza uralensis | Licochalcone B |
| Glycyrrhiza uralensis | licochalcone C |
| Glycyrrhiza uralensis | licochalconeD |
| Glycyrrhiza uralensis | glabrol |
| Glycyrrhiza uralensis | apioglycyrrhizin |
| Glycyrrhiza uralensis | apioglycyrrhizin_qt |
| Glycyrrhiza uralensis | 2,2-DIMETHYLPENTANE |
| Glycyrrhiza uralensis | licochalcone G |
| Glycyrrhiza uralensis | 3-(2,4-dihydroxyphenyl)-8-(1,1-dimethylprop-2-enyl)-7-hydroxy-5-methoxy-coumarin |
| Glycyrrhiza uralensis | 7-hydroxy-2-[4-hydroxy-3-(3-methylbut-2-enyl)phenyl]-6-(3-methylbut-2-enyl)chromone |
| Glycyrrhiza uralensis | Licoflavonol |
| Glycyrrhiza uralensis | Yinyanghuo D |
| Glycyrrhiza uralensis | 2,3-dimethylhexane |
| Glycyrrhiza uralensis | Prunetin |
| Glycyrrhiza uralensis | licorice glycoside E |
| Glycyrrhiza uralensis | (2R)-2-[3,4-dihydroxy-5-(3-methylbut-2-enyl)phenyl]-5,7-dihydroxy-8-(3-methylbut-2-enyl)chroman-4-one |
| Glycyrrhiza uralensis | 3-(3,4-dihydroxyphenyl)-5,7-dihydroxy-8-(3-methylbut-2-enyl)chromone |
| Glycyrrhiza uralensis | 5,7-dihydroxy-3-(4-methoxyphenyl)-8-(3-methylbut-2-enyl)chromone |
| Glycyrrhiza uralensis | 5,7-dihydroxy-3-(2-hydroxy-4-methoxy-phenyl)-6-(3-methylbut-2-enyl)chromone |
| Glycyrrhiza uralensis | 2-(3,4-dihydroxyphenyl)-5,7-dihydroxy-6-(3-methylbut-2-enyl)chromone |
| Glycyrrhiza uralensis | Gancaonin P |
| Glycyrrhiza uralensis | Gancaonin Q |
| Glycyrrhiza uralensis | Gancaonin R |
| Glycyrrhiza uralensis | Gancaonin S |
| Glycyrrhiza uralensis | (3S)-2,3-dimethylpentane |
| Glycyrrhiza uralensis | gancaonin T |
| Glycyrrhiza uralensis | Gancaonin U |
| Glycyrrhiza uralensis | Gancaonin V |
| Glycyrrhiza uralensis | 3-[4,6-dihydroxy-2-methoxy-3-(3-methylbut-2-enyl)phenyl]-7-hydroxy-chromone |
| Glycyrrhiza uralensis | Glycyram |
| Glycyrrhiza uralensis | 5,6,7,8-Tetrahydro-2,4-dimethylquinoline |
| Glycyrrhiza uralensis | (E)-1-[2,4-dihydroxy-3-(3-methylbut-2-enyl)phenyl]-3-(2,4-dihydroxyphenyl)prop-2-en-1-one |
| Glycyrrhiza uralensis | Licoisoflavone B |
| Glycyrrhiza uralensis | licoisoflavanone |
| Glycyrrhiza uralensis | licorice-saponin C2 |
| Glycyrrhiza uralensis | licorice-saponin C2_qt |
| Glycyrrhiza uralensis | licorice-saponin F3 |
| Glycyrrhiza uralensis | licorice-saponin F3_qt |
| Glycyrrhiza uralensis | (4S)-2,4-dimethylhexane |
| Glycyrrhiza uralensis | shinpterocarpin |
| Glycyrrhiza uralensis | licorice-saponin G2 |
| Glycyrrhiza uralensis | licorice-saponin G2_qt |
| Glycyrrhiza uralensis | licorice-saponin H2 |
| Glycyrrhiza uralensis | licorice-saponin H2_qt |
| Glycyrrhiza uralensis | licorice-saponin J2 |
| Glycyrrhiza uralensis | licorice-saponin J2_qt |
| Glycyrrhiza uralensis | (E)-3-[3,4-dihydroxy-5-(3-methylbut-2-enyl)phenyl]-1-(2,4-dihydroxyphenyl)prop-2-en-1-one |
| Glycyrrhiza uralensis | licorice-saponin B2 |
| Glycyrrhiza uralensis | licorice-saponin K2 |
| Glycyrrhiza uralensis | licorice-saponin K2_qt |
| Glycyrrhiza uralensis | 3,22-Dihydroxy-11-oxo-delta(12)-oleanene-27-alpha-methoxycarbonyl-29-oic acid |
| Glycyrrhiza uralensis | Glabridin |
| Glycyrrhiza uralensis | Glabranin |
| Glycyrrhiza uralensis | Glabrene |
| Glycyrrhiza uralensis | Glabrone |
| Glycyrrhiza uralensis | 1,3-dihydroxy-9-methoxy-6-benzofurano[3,2-c]chromenone |
| Glycyrrhiza uralensis | 1,3-dihydroxy-8,9-dimethoxy-6-benzofurano[3,2-c]chromenone |
| Glycyrrhiza uralensis | Eurycarpin A |
| Glycyrrhiza uralensis | 2-methyl-5-propyl -nonane |
| Glycyrrhiza uralensis | HEX |
| Glycyrrhiza uralensis | Sextone B |
| Glycyrrhiza uralensis | Methylcyclopentane |
| Glycyrrhiza uralensis | Docosyl caffeate |
| Glycyrrhiza uralensis | 2-methyl-6-ethyl decane |
| Glycyrrhiza uralensis | (-)-Medicocarpin |
| Glycyrrhiza uralensis | vitexin |
| Glycyrrhiza uralensis | 4H-1-Benzopyran-4-one, 2-(4-(beta-D-glucopyranosyloxy)phenyl)-2,3-dihydro-5,7-dihydroxy-, (2S)- |
| Glycyrrhiza uralensis | violanthin |
| Glycyrrhiza uralensis | Pentadecanol |
| Glycyrrhiza uralensis | Isohexane |
| Glycyrrhiza uralensis | Sigmoidin-B |
| Glycyrrhiza uralensis | Nortangeretin |
| Glycyrrhiza uralensis | (2R)-7-hydroxy-2-(4-hydroxyphenyl)chroman-4-one |
| Glycyrrhiza uralensis | (E)-dodec-2-ene |
| Glycyrrhiza uralensis | Cyclobutanol, 1-ethyl- |
| Glycyrrhiza uralensis | (2S)-7-hydroxy-2-(4-hydroxyphenyl)-8-(3-methylbut-2-enyl)chroman-4-one |
| Glycyrrhiza uralensis | 2-Tetradecanone |
| Glycyrrhiza uralensis | Isoviolanthin |
| Glycyrrhiza uralensis | isoglycycoumarin |
| Glycyrrhiza uralensis | licuraside |
| Glycyrrhiza uralensis | Liquiritin apioside |
| Glycyrrhiza uralensis | isograbrol |
| Glycyrrhiza uralensis | isoglabrolide |
| Glycyrrhiza uralensis | HMO |
| Glycyrrhiza uralensis | 1-Methoxyphaseollidin |
| Glycyrrhiza uralensis | 22β-acetylglabric acid |
| Glycyrrhiza uralensis | Quercetin der. |
| Glycyrrhiza uralensis | 24-Hydroxy-11-deoxyglycyrrhetic acid |
| Glycyrrhiza uralensis | 24-Hydroxyglycyrrhetic acid |
| Glycyrrhiza uralensis | (Z)-1-(2,4-dihydroxyphenyl)-3-phenylprop-2-en-1-one |
| Glycyrrhiza uralensis | 3'(γ,γ-dimethylallyl)-kievitone |
| Glycyrrhiza uralensis | 3'-Hydroxy-4'-O-Methylglabridin |
| Glycyrrhiza uralensis | 3,3-Dimethylpentane |
| Glycyrrhiza uralensis | 3,4,3',4'-Tetrahydroxy-2-methoxychalcone |
| Glycyrrhiza uralensis | 2-Ethyl-p-xylene |
| Glycyrrhiza uralensis | 3-methylheptane |
| Glycyrrhiza uralensis | 3-methylhexane |
| Glycyrrhiza uralensis | 3-Methylpentane |
| Glycyrrhiza uralensis | 3-Ethylpentane |
| Glycyrrhiza uralensis | 3β-formylglabrolide |
| Glycyrrhiza uralensis | Daidzein dimethyl ether |
| Glycyrrhiza uralensis | 1-Methoxyficifolinol |
| Glycyrrhiza uralensis | 2-[(3R)-8,8-dimethyl-3,4-dihydro-2H-pyrano[6,5-f]chromen-3-yl]-5-methoxyphenol |
| Glycyrrhiza uralensis | 4,2',4',alpha-Tetrahydroxydihydrochalcone |
| Glycyrrhiza uralensis | Inflacoumarin A |
| Glycyrrhiza uralensis | 1-(5-hydroxy-2,2-dimethylchromen-6-yl)-3-(4-hydroxyphenyl)prop-2-en-1-one |
| Glycyrrhiza uralensis | 2,6,10-trimethyl-dodecane |
| Glycyrrhiza uralensis | Licoriisoflavan A |
| Glycyrrhiza uralensis | icos-5-enoic acid |
| Glycyrrhiza uralensis | 6″-O-acetylliquiritin |
| Glycyrrhiza uralensis | 11-deoxyglycyrrhetic acid |
| Glycyrrhiza uralensis | Kanzonol F |
| Glycyrrhiza uralensis | 6-prenylated eriodictyol |
| Glycyrrhiza uralensis | 7,2',4'-trihydroxy－5-methoxy-3－arylcoumarin |
| Glycyrrhiza uralensis | 7-Acetoxy-2-methylisoflavone |
| Glycyrrhiza uralensis | 7-hydroxy-2-methyl-3-phenyl-chromone |
| Glycyrrhiza uralensis | 8-prenylated eriodictyol |
| Glycyrrhiza uralensis | 12-methyltetradecanoate |
| Glycyrrhiza uralensis | Kanzonol H |
| Glycyrrhiza uralensis | gadelaidic acid |
| Glycyrrhiza uralensis | Araboglycyrrhizin |
| Glycyrrhiza uralensis | Araboglycyrrhizin_qt |
| Glycyrrhiza uralensis | Artonin E |
| Glycyrrhiza uralensis | Vestitol |
| Glycyrrhiza uralensis | Gancaonin G |
| Glycyrrhiza uralensis | Gancaonin H |
| Glycyrrhiza uralensis | beta-Glycyrrhetinic acid |
| Glycyrrhiza uralensis | Licoagrocarpin |
| Glycyrrhiza uralensis | Glyasperin A |
| Glycyrrhiza uralensis | Glyasperins K |
| Glycyrrhiza uralensis | Glyasperins M |
| Glycyrrhiza uralensis | Glycyrrhiza flavonol A |
| Glycyrrhiza uralensis | Kanzonol E |
| Glycyrrhiza uralensis | Kanzonol Z |
| Glycyrrhiza uralensis | Licoagroisoflavone |
| Glycyrrhiza uralensis | 18α-hydroxyglycyrrhetic acid |
| Glycyrrhiza uralensis | Licorice glycoside A |
| Glycyrrhiza uralensis | Odoratin |
| Glycyrrhiza uralensis | Phaseol |
| Glycyrrhiza uralensis | Xambioona |
| Glycyrrhiza uralensis | (2R)-7-hydroxy-2-[4-hydroxy-3-(3-methylbut-2-enyl)phenyl]chroman-4-one |
| Glycyrrhiza uralensis | dehydroglyasperins C |
| Glycyrrhiza uralensis | Mipax |
| Glycyrrhiza uralensis | ursolic acid |
| Glycyrrhiza uralensis | DIBP |
| Glycyrrhiza uralensis | PENTYLFURAN |
| Glycyrrhiza uralensis | ()-Menthol |
| Glycyrrhiza uralensis | DBP |
| Glycyrrhiza uralensis | 2-heptanone |
| Glycyrrhiza uralensis | WLN: VH6 |
| Glycyrrhiza uralensis | quercetin |
| Ophiopogon japonicus | 2'-hydroxymethylophiopogonone a |
| Ophiopogon japonicus | 2,5,7-trihydroxy-6,8-dimethyl-3-(3',4'-methyl-enedioxybenzyl)-chroman-4-one |
| Ophiopogon japonicus | 2,5,7-trihydroxy-6,8-dimethyl-3-(4'-methoxy-benzyl)chroman-4-one |
| Ophiopogon japonicus | 25(s)-ruscogenin 1-o-alpha-l-rhamnopy-ranosyl-(1-2)-beta-d-xylopyranoside |
| Ophiopogon japonicus | 25(s)-ruscogenin 1-o-[α-l-rhamnopyranosyl-(1→2)][β-d-xylopyra-nosyl-(1→3)]-β-d-fucopyranoside |
| Ophiopogon japonicus | 5,7,2'-trihydroxy-6-methyl-3-(3',4'-methylene-dioxybenzyl)chromone |
| Ophiopogon japonicus | 5,7-dihydroxy-6,8-dime thyl-3-(4'-hydroxy-3'-methoxybenzyl)chroman-4-one |
| Ophiopogon japonicus | 5-hydroxy-7,8-dimethoxy-6-methyl-3-(3',4'-dihydroxybenzyl)chroman-4-one |
| Ophiopogon japonicus | 6-aldehydo-isoophiopogone a |
| Ophiopogon japonicus | 6-aldehydo-isoophiopogone b |
| Ophiopogon japonicus | 6-aldehydo-isoophipogonone a |
| Ophiopogon japonicus | 6-aldehydo-isoophipogonone b |
| Ophiopogon japonicus | adeninenucleoside |
| Ophiopogon japonicus | borneol-2-o-α-l-arabinofuranosyl(1→6)-β-d-glucopyranoside |
| Ophiopogon japonicus | borneol-2-o-β-d-apiofuranosyl(1→6)-β-d-glucopyranoside |
| Ophiopogon japonicus | borneol-2-o-β-d-glucopyranoside |
| Ophiopogon japonicus | diosgenin |
| Ophiopogon japonicus | guanosine |
| Ophiopogon japonicus | isoophiopogonone a |
| Ophiopogon japonicus | jasmololone |
| Ophiopogon japonicus | methy-lophiopogonone a |
| Ophiopogon japonicus | methy-lophiopogonone b |
| Ophiopogon japonicus | methyl beta-orcinol caroxylate |
| Ophiopogon japonicus | methyl ophiopogonanone a |
| Ophiopogon japonicus | methyl ophiopogonanone b |
| Ophiopogon japonicus | n-(trans-p-coumaroyl)tyramine |
| Ophiopogon japonicus | n-trans-feruloyltyramine |
| Ophiopogon japonicus | n-[β-hydroxy-β-(4-hydroxyphenyl)]ethyl-4-hydroxy cinnamide |
| Ophiopogon japonicus | oleanolicacid |
| Ophiopogon japonicus | ophiogenin-3-o-α-l-rhamnopyranosyl(1→2)-β-d-glucopyranoside |
| Ophiopogon japonicus | ophiopogon a |
| Ophiopogon japonicus | ophiopogon b |
| Ophiopogon japonicus | ophiopogonanone a |
| Ophiopogon japonicus | ophiopogonanone b |
| Ophiopogon japonicus | ophiopogonanone c |
| Ophiopogon japonicus | ophiopogonanone d |
| Ophiopogon japonicus | ophiopogonanone e |
| Ophiopogon japonicus | ophiopogonanone f |
| Ophiopogon japonicus | ophiopogonin a |
| Ophiopogon japonicus | ophiopogonin b |
| Ophiopogon japonicus | ophiopogonin c |
| Ophiopogon japonicus | ophiopogonin c' |
| Ophiopogon japonicus | ophiopogonin d |
| Ophiopogon japonicus | ophiopogonin d' |
| Ophiopogon japonicus | ophiopogonone a |
| Ophiopogon japonicus | ophiopogonone b |
| Ophiopogon japonicus | ophiopogonone c |
| Ophiopogon japonicus | ophiopogonoside a |
| Ophiopogon japonicus | orchinol |
| Ophiopogon japonicus | ruscogenin |
| Ophiopogon japonicus | ruscogenin 1-o-sulfate |
| Ophiopogon japonicus | stigmasterol |
| Ophiopogon japonicus | stigmasterol-beta-d-glucoside |
| Ophiopogon japonicus | uridine |
| Ophiopogon japonicus | β-patchoulene |
| Morus alba | (-)-guaia-1(10),11-dien-15-al |
| Morus alba | (6s,9r)-roseoside |
| Morus alba | 1'-methoxy-2'-hydroxydihydromollugin |
| Morus alba | 2',4'-dihydroxy-7-methoxy-8-prenylflavan |
| Morus alba | 2',7-dihydroxy-4'-methoxy-8-prenylflavan |
| Morus alba | 2',7-dihydroxy-4'-methoxy-8-prenylflavan2',7-di-o-β-d-glucopyranoside |
| Morus alba | 26-hydroxy-dammara-20,24-dien-3-one |
| Morus alba | 3'-o-methyl sappanol |
| Morus alba | 3,4-dihydroxydihydgaroaroiuran |
| Morus alba | 3-hydroxycoumarin |
| Morus alba | 4-hydroxycoumarin |
| Morus alba | 4-prenylresveratrol |
| Morus alba | 5,7-dihydroxychromone |
| Morus alba | 5-hydroxycoumarin |
| Morus alba | 6-hydroxycoumarin |
| Morus alba | 7alpha,21s,25-trihydroxy-3beta-acetoxy-21s,23r-epoxy-9(11)-en-dammarane |
| Morus alba | adenine |
| Morus alba | albafuran a |
| Morus alba | albanol |
| Morus alba | albanol a |
| Morus alba | alpha, beta-hexenal |
| Morus alba | amygdalin |
| Morus alba | astragalin |
| Morus alba | benzyl alcoholo-β-d-glucopyranoside |
| Morus alba | beta-sitosterol-3-o-beta-d-xylopyranoside |
| Morus alba | campesterol |
| Morus alba | campesteryl ferulate |
| Morus alba | chlorogenic acid |
| Morus alba | chlorogenicacid |
| Morus alba | cudranin |
| Morus alba | ecdysterone |
| Morus alba | ecdysterone-3-o-beta-d-glucopyranoside |
| Morus alba | eugenol |
| Morus alba | eugenol methyl ether |
| Morus alba | folicacid |
| Morus alba | folinic acid |
| Morus alba | folinicacid |
| Morus alba | foliosidine |
| Morus alba | fumaric acid |
| Morus alba | guaiacol |
| Morus alba | inokosterone |
| Morus alba | inophyllolide |
| Morus alba | inositol c |
| Morus alba | insularine |
| Morus alba | isobutyric acid |
| Morus alba | isobutyricacid |
| Morus alba | isobutyrylmaeelotochromene |
| Morus alba | isoquercitrin |
| Morus alba | isoramanone |
| Morus alba | isovalericacid |
| Morus alba | isovaleroxy-hydroxy dihydrovaltrate |
| Morus alba | iupeol |
| Morus alba | kuwanon c |
| Morus alba | kuwanon g |
| Morus alba | kuwanon h |
| Morus alba | kuwanone h |
| Morus alba | kwangsine |
| Morus alba | lupeol |
| Morus alba | lupeol acetate |
| Morus alba | m-cresol |
| Morus alba | maclurin |
| Morus alba | macranthoidin a |
| Morus alba | methyl salicylate |
| Morus alba | moracetin |
| Morus alba | moracin b |
| Morus alba | moracin c |
| Morus alba | moracin d |
| Morus alba | moracin e |
| Morus alba | moracin f |
| Morus alba | moracin g |
| Morus alba | moracin h |
| Morus alba | morellic acid |
| Morus alba | morin |
| Morus alba | morindin |
| Morus alba | morusin |
| Morus alba | moscatin |
| Morus alba | mulberrofuran a |
| Morus alba | mulberrofuran b |
| Morus alba | myoinositol |
| Morus alba | oxysanguinarine |
| Morus alba | p-cresol |
| Morus alba | paeonol |
| Morus alba | paeonolide |
| Morus alba | pentanicacid |
| Morus alba | quercetin |
| Morus alba | riboflavine |
| Morus alba | rutin |
| Morus alba | scopeletin |
| Morus alba | scopoletin |
| Morus alba | scopolin |
| Morus alba | skimmin |
| Morus alba | trigonelline |
| Morus alba | umbelliferone |
| Morus alba | vitamin b1 |
| Morus alba | β-sitosterol-β-d-glucoside |
| Morus alba | poriferast-5-en-3beta-ol |
| Morus alba | Methyleugenol |
| Morus alba | PCR |
| Morus alba | beta-carotene |
| Morus alba | (3R,4S)-3-(4-hydroxy-3-methoxy-benzyl)chroman-3,4,7-triol |
| Morus alba | Ecdysterone-3-O-beta-D-glucopyranoside_qt |
| Morus alba | 8-[(2S)-2,3-dihydroxy-3-methyl-butoxy]-4-methoxy-1-methyl-carbostyril |
| Morus alba | Inophyllum E |
| Morus alba | Isobutyrylmallotochromene |
| Morus alba | Macranthoidin A_qt |
| Morus alba | Morindon |
| Morus alba | gynesine |
| Morus alba | α－ionone |
| Morus alba | Inositol |
| Morus alba | 7-Hydroxycoumarin |
| Morus alba | (2R)-2-[[4-[[(6S)-2-amino-5-formyl-4-keto-1,6,7,8-tetrahydropteridin-6-yl]methylamino]benzoyl]amino]glutaric acid |
| Morus alba | Fuseloel |
| Morus alba | Hirsutrin |
| Morus alba | choline |
| Morus alba | Scopoletol |
| Morus alba | FA |
| Morus alba | WLN: VHR |
| Morus alba | LINALOOL (D) |
| Morus alba | Arachic acid |
| Morus alba | Hemo-sol |
| Morus alba | alpha-Curcumene |
| Morus alba | beta-Selinene |
| Morus alba | DIBP |
| Morus alba | Prolinum |
| Morus alba | palmitic acid |
| Morus alba | protocatechuic acid |
| Morus alba | vanillic acid |
| Morus alba | Nonanal |
| Morus alba | 1,8-cineole |
| Morus alba | EIC |
| Morus alba | (R)-linalool |
| Morus alba | p-Ocimene |
| Morus alba | ()-Aromadendrene |
| Morus alba | L-Limonen |
| Morus alba | Rhamnocitrin |
| Morus alba | farnesol |
| Morus alba | oleanolic acid |
| Morus alba | Sitogluside |
| Morus alba | beta-sitosterol |
| Morus alba | Amyrin |
| Morus alba | O-Acetyl-beta-amyrin |
| Morus alba | gamma-aminobutyric acid |
| Morus alba | Docosanoate |
| Morus alba | kaempferol |
| Morus alba | linolenic acid |
| Morus alba | Stigmasterol |
| Morus alba | anethole |
| Morus alba | Physcion |
| Morus alba | Farnesene |
| Morus alba | TMH |
| Morus alba | Friedelin |
| Morus alba | ursolic acid |
| Morus alba | Nonacosane |
| Morus alba | Hederagenol |
| Morus alba | Dehydro-p-cymene |
| Morus alba | TRD |
| Morus alba | vanillin |
| Morus alba | Montanic acid |
| Morus alba | PENTACOSANOIC ACID |
| Morus alba | Ceric acid |
| Morus alba | lignoceric acid |
| Morus alba | hexanal |
| Morus alba | 1-hexanol |
| Morus alba | Farnesol acetate |
| Morus alba | oleic acid |
| Morus alba | DBP |
| Morus alba | m-Cymol |
| Morus alba | Hyacinthin |
| Morus alba | Safranal |
| Morus alba | Geranylacetone |
| Morus alba | EEE |
| Morus alba | OXA |
| Morus alba | sucrose |
| Morus alba | stearic acid |
| Morus alba | Dekan |
| Morus alba | MYS |
| Morus alba | hexadecane |
| Morus alba | Heptadekan |
| Morus alba | LFA |
| Morus alba | Henicosane |
| Morus alba | Cedrol |
| Morus alba | methyl palmitate |
| Morus alba | Dodekan |
| Morus alba | tetradecane |
| Morus alba | ()-beta-Pinene |
| Morus alba | (5S)-1-isopropyl-4-methylbicyclo[3.1.0]hex-3-ene |
| Morus alba | cis-beta-farnesene |
| Morus alba | alpha-Farnesene |
| Morus alba | Ethylpalmitate |
| Morus alba | CRS |
| Morus alba | p-xylene |
| Morus alba | cis-beta-Ocimene |
| Morus alba | Tip-Nip |
| Morus alba | PEL |
| Morus alba | WLN: Q1R |
| Morus alba | Benzyl glucopyranoside |
| Morus alba | Methylheptadienone |
| Morus alba | myristic acid |
| Morus alba | Oktadekan |
| Morus alba | Methyllinolenate |
| Morus alba | TWT |
| Morus alba | HEXACOSANE |
| Morus alba | Octacosane |
| Morus alba | arachidonic acid |
| Morus alba | phytol |
| Morus alba | FITONE |
| Morus alba | Daturic acid |
| Morus alba | Supraene |
| Morus alba | Isocaryophyllene |
| Morus alba | Nonadecene |
| Morus alba | Hypnon |
| Morus alba | [(3S)-3,7-dimethylocta-1,6-dien-3-yl] propanoate |
| Morus alba | UPL |
| Morus alba | 1-Tetradecene |
| Morus alba | vitamin c |
| Morus alba | Quercimeritrin (6CI,7CI,8CI) |
| Morus alba | zoomaric acid |
| Morus alba | Tetracosane |
| Morus alba | Fleet-X |
| Morus alba | Majudin |
| Morus alba | Heriguard |
| Morus alba | TRICOSANOIC ACID |
| Morus alba | hexanoic acid |
| Morus alba | DEP |
| Morus alba | 1,3,8-p-Menthatriene |
| Morus alba | OCT |
| Morus alba | HEPTACOSANE |
| Morus alba | DLA |
| Morus alba | Terragon |
| Morus alba | beta-Ionone |
| Morus alba | gamma-tocopherol |
| Morus alba | PENTACOSANE |
| Morus alba | UND |
| Morus alba | PTL |
| Morus alba | cis-Anethol |
| Morus alba | Sulcatone |
| Morus alba | beta-Rhodinol |
| Morus alba | Skimmetin |
| Morus alba | Hexenal |
| Morus alba | Iva |
| Morus alba | nicotiflorin |
| Morus alba | (4S)-4-hydroxy-3,5,5-trimethyl-4-[(E,3R)-3-[(2R,3R,4S,5S,6R)-3,4,5-trihydroxy-6-(hydroxymethyl)tetrahydropyran-2-yl]oxybut-1-enyl]cyclohex-2-en-1-one |
| Morus alba | VIV |
| Morus alba | 5,8-dimethyltocol |
| Morus alba | WLN: QV4 |
| Morus alba | Ethyl caffeate |
| Morus alba | BuOH |
| Morus alba | (4S)-1-methyl-4-(6-methylhepta-1,5-dien-2-yl)cyclohexene |
| Morus alba | Guasol |
| Morus alba | IPH |
| Morus alba | Amylol |
| Morus alba | CIS-2-PENTENOL |
| Morus alba | Isobutyral |
| Morus alba | naphthalene |
| Morus alba | Damascenone |
| Morus alba | cis-p-2,8-Menthadien-1-ol |
| Morus alba | Iristectorigenin A |
| Morus alba | tectorigenin |
| Morus alba | 2,6-Dimethyl-1,3,5,7-octatetraene, E,E- |
| Morus alba | icosa-11,14,17-trienoic acid methyl ester |
| Morus alba | Isotrifolin |
| Morus alba | Perillen |
| Morus alba | MEK |
| Morus alba | PPI |
| Morus alba | Azeton |
| Morus alba | o-cresol |
| Morus alba | acetic acid |
| Morus alba | Methyl naphthalene |
| Morus alba | 2-octanone |
| Morus alba | beta-Terpinene |
| Morus alba | NOJ |
| Morus alba | Butal |
| Morus alba | FAG |
| Morus alba | DAL |
| Morus alba | (7aR)-4,4,7a-trimethyl-6,7-dihydro-5H-benzofuran-2-one |
| Morus alba | 2-HEXENE |
| Morus alba | (E)-1-[2,4-dihydroxy-3-(3-methylbut-2-enyl)phenyl]-3-(2,4-dihydroxyphenyl)prop-2-en-1-one |
| Morus alba | Corylifolinin |
| Morus alba | .alpha.-Ionene |
| Morus alba | Octadecanal |
| Morus alba | 1,2-Benzenedicarboxylicacid, mono(2-ethyl) hexylester |
| Morus alba | Eicosene |
| Morus alba | Linolenyl alcohol |
| Morus alba | i-Butanol |
| Morus alba | Psi-cumene |
| Morus alba | 2,6,11-trimethyldodecane |
| Morus alba | Zyklohexen |
| Morus alba | Nonadecylic acid |
| Morus alba | Norartocarpetin |
| Morus alba | (1R,3S,6S)-3,7,7-trimethylbicyclo[4.1.0]hept-4-ene |
| Morus alba | Linolenic acid ethyl ester |
| Morus alba | 1,2-DIHYDRO-1,5,8-TRIMETHYLNAPHTHALENE |
| Morus alba | Tetramethoxyluteolin |
| Morus alba | BUA |
| Morus alba | Henicosanoic acid |
| Morus alba | ZINC00901303 |
| Morus alba | Hex-3-enyl acetate |
| Morus alba | I-Amyl acetate |
| Morus alba | methyl 8-methyl-decanoate |
| Morus alba | Isoamyl benzoate |
| Morus alba | D-Asparaginsaeure |
| Morus alba | 8-methyltocol |
| Morus alba | 2-Hexenol |
| Morus alba | Leaf acetate |
| Morus alba | Linolenic acid methyl ester |
| Morus alba | bergamotene (Z,.alpha.,cis) |
| Morus alba | gamma-Hexenol |
| Morus alba | HBR |
| Morus alba | Nerylacetone |
| Morus alba | 5,7-Dihydroxycoumarin |
| Morus alba | morusignin L |
| Morus alba | Decahydro-2,3-dimethyl-naphthalene |
| Morus alba | 3,5-Octadien-2-one, (E,E)- |
| Morus alba | ZINC02034403 |
| Morus alba | 6-methoxy-2-oxo-2H-chromen-7-yl beta-D-glucopyranoside |
| Morus alba | Skimmin (8CI) |
| Morus alba | Inden |
| Trichosanthes kirilowii | (e,z,e)-7-hydroxymethyl-3,11,15-trimethyl-2,6,10,14-hexadecatetraen-1-ol |
| Trichosanthes kirilowii | 1,5-dimethylnaphthalene |
| Trichosanthes kirilowii | 1-(2-furanyl)ethanone |
| Trichosanthes kirilowii | 1-hexanol |
| Trichosanthes kirilowii | 1-nonanol |
| Trichosanthes kirilowii | 1-octen-3-ol |
| Trichosanthes kirilowii | 2,3-butanediol |
| Trichosanthes kirilowii | 2,4-dimethylphenol |
| Trichosanthes kirilowii | 2-furancarboxaldehyde |
| Trichosanthes kirilowii | 2-methylnaphthalene |
| Trichosanthes kirilowii | 2-pentadecanone |
| Trichosanthes kirilowii | 20-hexadecanoylingenol |
| Trichosanthes kirilowii | 3-methyl-1-butanol |
| Trichosanthes kirilowii | 3-methylphenanthrene |
| Trichosanthes kirilowii | 4-hydroxy-4-methyl-2-pentanone |
| Trichosanthes kirilowii | 4-methyldibenzofuran |
| Trichosanthes kirilowii | 7-stigmastenol-3-o-β-d-glucoside |
| Trichosanthes kirilowii | 9-(z)-octadecen-1-ol |
| Trichosanthes kirilowii | acenaphthylene |
| Trichosanthes kirilowii | acetoin |
| Trichosanthes kirilowii | anthracene |
| Trichosanthes kirilowii | benzaldehyde |
| Trichosanthes kirilowii | benzeneethanol |
| Trichosanthes kirilowii | benzenemethanol |
| Trichosanthes kirilowii | bryonolic acid |
| Trichosanthes kirilowii | bryonolicacid |
| Trichosanthes kirilowii | cedrenol |
| Trichosanthes kirilowii | cis-9,cis-12-linoleicacid |
| Trichosanthes kirilowii | citrulline |
| Trichosanthes kirilowii | citrusin b |
| Trichosanthes kirilowii | delta7-stigmastenone-3 |
| Trichosanthes kirilowii | dibenzofuran |
| Trichosanthes kirilowii | dibutyl phthalate |
| Trichosanthes kirilowii | docosane |
| Trichosanthes kirilowii | ethyl hexadecanoate |
| Trichosanthes kirilowii | ethyl linoleate |
| Trichosanthes kirilowii | ethyl linolenate |
| Trichosanthes kirilowii | ethyl n-pentadecanoate |
| Trichosanthes kirilowii | ethyl pentadecanoate |
| Trichosanthes kirilowii | ethyl stearate |
| Trichosanthes kirilowii | gamma-aminobutyric acid |
| Trichosanthes kirilowii | geranylacetone |
| Trichosanthes kirilowii | heptadecane |
| Trichosanthes kirilowii | hexadecane |
| Trichosanthes kirilowii | hexadecanoicacid |
| Trichosanthes kirilowii | hexahydrofarnesylacetone |
| Trichosanthes kirilowii | linolenicacid |
| Trichosanthes kirilowii | methyl hexadecanoate |
| Trichosanthes kirilowii | methyl linoleate |
| Trichosanthes kirilowii | methyl linolenate |
| Trichosanthes kirilowii | methyl n-pentadecanoate |
| Trichosanthes kirilowii | methyl pentadecanoate |
| Trichosanthes kirilowii | methyl stearate |
| Trichosanthes kirilowii | methyl tetradecanoate |
| Trichosanthes kirilowii | n-octadecane |
| Trichosanthes kirilowii | naphthalene |
| Trichosanthes kirilowii | nonadecane |
| Trichosanthes kirilowii | nonanal |
| Trichosanthes kirilowii | phenanthrene |
| Trichosanthes kirilowii | phytol |
| Trichosanthes kirilowii | pyrene |
| Trichosanthes kirilowii | spinoside a |
| Trichosanthes kirilowii | trans-2-nonenal |
| Trichosanthes kirilowii | trichosanthin |
| Trichosanthes kirilowii | α-hydroxymethylserine |
| Trichosanthes kirilowii | α-spinasterol-β-d-glucoside |
| Trichosanthes kirilowii | γ-aminobutyricacid |
| Trichosanthes kirilowii | cucurbitacin b |
| Trichosanthes kirilowii | Elatericin A |
| Trichosanthes kirilowii | CIR |
| Trichosanthes kirilowii | Spinasterol |
| Trichosanthes kirilowii | (2R,3R,4S,5S,6R)-2-[[(3S,5S,9R,10S,13R,14R,17R)-17-[(E,2R,5S)-5-ethyl-6-methylhept-3-en-2-yl]-10,13-dimethyl-2,3,4,5,6,9,11,12,14,15,16,17-dodecahydro-1H-cyclopenta[a]phenanthren-3-yl]oxy]-6-(hydroxymethyl)oxane-3,4,5-triol |
| Trichosanthes kirilowii | GUP |
| Trichosanthes kirilowii | DIHYDROCUCURBITACIN B |
| Trichosanthes kirilowii | Schottenol |
| Trichosanthes kirilowii | delta7-Stigmastenol-3-O-beta-D-glucoside |
| Trichosanthes kirilowii | poriferast-7-en-3beta-ol |
| Trichosanthes kirilowii | (R)-ornithine |
| Trichosanthes kirilowii | Schottenol glucoside |
| Trichosanthes kirilowii | 2-Hydroxymethylserine |
| Polygonatum odoratum | 22-hydroxy-25(r,s)-furost-5-en-12-on-3β,22,26-triol 26-o-β-d-glucopyranoside |
| Polygonatum odoratum | azetidine-2-carboxylic acid |
| Polygonatum odoratum | azetidine-2-carboxylicacid |
| Polygonatum odoratum | convallamarin |
| Polygonatum odoratum | convallarin |
| Polygonatum odoratum | convallasaponin a |
| Polygonatum odoratum | polyfuroside |
| Polygonatum odoratum | polyfuroside po6 |
| Polygonatum odoratum | polyfuroside po7 |
| Polygonatum odoratum | polyfuroside po8 |
| Polygonatum odoratum | quercitol |
| Polygonatum odoratum | wattoside c |
| Polygonatum odoratum | 7'-n-butanol-N-trans-feruloyloctopamine |
| Polygonatum odoratum | ()-alpha-Funebrene |
| Polygonatum odoratum | Azetidinecarboxylic acid |
| Polygonatum odoratum | Convallasaponin A_qt |
| Polygonatum odoratum | 3-o-beta-d-glucopyranosyl-(1-2)-[beta-d-xylopyranosyl-(1-3)]-beta-d-glucopyranosyl-(1-4)-galactopyranosyl-25(S)-spirost-5(6)-en-3beta-ol |
| Polygonatum odoratum | 3-o-beta-d-glucopyranosyl-(1-2)-[beta-d-xylopyranosyl-(1-3)]-beta-d-glucopyranosyl-(1-4)-galactopyranosyl-25(S)-spirost-5(6)-en-3beta-ol_qt |
| Polygonatum odoratum | 3-o-beta-d-glucopyranosyl-(1-2)-[beta-d-xylopyranosyl-(1-3)]-beta-d-glucopyranosyl-(1-4)-galactopyranosyl-25(S)-spirost-5(6)-en-3beta,14alpho-diol |
| Polygonatum odoratum | 3-o-beta-d-glucopyranosyl-(1-2)-[beta-d-xylopyranosyl-(1-3)]-beta-d-glucopyranosyl-(1-4)-galactopyranosyl-25(S)-spirost-5(6)-en-3beta,14alpho-diol_qt |
| Polygonatum odoratum | 25(R)spirost-5-en-3beta-ol-3-o-beta-d-glucopyranosyl-(1-2)-[beta-d-xylylpyranylosyl-(1-3)]-beta-d-glucopyranosyl-(1-4)-beta-d-galactopyranose |
| Polygonatum odoratum | 25(R)spirost-5-en-3beta-ol-3-o-beta-d-glucopyranosyl-(1-2)-[beta-d-xylylpyranylosyl-(1-3)]-beta-d-glucopyranosyl-(1-4)-beta-d-galactopyranose_qt |
| Polygonatum odoratum | 25(S and R)-spirost-5-en-3beta,14alpho-diol |
| Polygonatum odoratum | 25(R,S)spirost-5-en-3beta-ol-3-o-beta-d-glucopyranosyl-(1-2)-[beta-d-xylylpyranylosyl-(1-3)]-beta-d-glucopyranosyl-(1-4)-beta-d-galactopyranose |
| Polygonatum odoratum | 25(R,S)spirost-5-ene-3beta,14alpha-diol-3-o-beta-d-glucopyranosyl-(1-2)-[beta-d-glucopyranosyl-(1-3)]-beta-d-glucopyranosyl-(1-4)-beta-d-galactopyranose |
| Polygonatum odoratum | 25(R,S)spirost-5-ene-3beta,14alpha-diol-3-o-beta-d-glucopyranosyl-(1-2)-[beta-d-xylylpyranylosyl-(1-3)]-beta-d-glucopyranosyl-(1-4)-beta-d-galactopyranose |
| Polygonatum odoratum | STOCK1N-66370 |
| Polygonatum odoratum | 4',5,7-trihydroxy-6-methyl-8-methoxy-homoisoflavanone |
| Polygonatum odoratum | 4',5,7-trihydroxy-6,8-dimethyl-homoisoflavanone |
| Polygonatum odoratum | (3beta,14alpha)-3-o-beta-d-glucopyranosyl-(1-2)-[beta-d-xylopyranosyl-(1-3)]-beta-d-glucopyranosyl(1-4)-beta-d-galacopyranosyl-14-hydroxy-(25S)-spirost-5-ene |
| Polygonatum odoratum | polygosides A_qt |
| Polygonatum odoratum | (3beta,14alpha)-3-o-beta-d-glucopyranosyl-(1-2)-[beta-d-xylopyranosyl-(1-3)]-beta-d-glucopyranosyl-(1-4)-beta-d-galacopyranosyl-yamogenin |
| Polygonatum odoratum | (22S)-sipirost-5-ene-1beta,3beta,16beta,22-tetrol-1-o-alpha-l-rhamnopyranosyl-16-o-beta-d-glucopyranoside |
| Polygonatum odoratum | (22S)-sipirost-5-ene-1beta,3beta,16beta,22-tetrol-1-o-alpha-l-rhamnopyranosyl-16-o-beta-d-glucopyranoside_qt |
| Polygonatum odoratum | polyfuroside_qt |
| Polygonatum odoratum | polygosides A |
| Polygonatum odoratum | polygosides B |
| Polygonatum odoratum | polygosides B_qt |
| Polygonatum odoratum | polygosides E |
| Polygonatum odoratum | polygosides E_qt |
| Polygonatum odoratum | polygosides D |
| Polygonatum odoratum | 2-Methylnonan-3-one |
| Polygonatum odoratum | 4',5,7-trihydroxy-6-methyl-homoisoflavanone |
| Polygonatum odoratum | 4'-methoxy-5,7-dihydroxy-6,8-dimethyl-homoisflavanone |
| Polygonatum odoratum | 4-methyl-2-hexanone |
| Polygonatum odoratum | Nonanal |
| Polygonatum odoratum | 7-TETRADECENE |
| Polygonatum odoratum | oleic acid |
| Polygonatum odoratum | PENTADECYLIC ACID |
| Polygonatum odoratum | zoomaric acid |
| Polygonatum odoratum | Hexenal |
| Polygonatum odoratum | [(1S)-endo]-(-)-Borneol |
| Polygonatum odoratum | lauric acid |
| Polygonatum odoratum | n-coumaroyltyramine |
| Polygonatum odoratum | Yamogenin |
| Polygonatum odoratum | CHEBI:39932 |
| Polygonatum odoratum | glucuronic acid |
| Polygonatum odoratum | (Z)-3-(4-hydroxy-3-methoxy-phenyl)-N-[2-(4-hydroxyphenyl)ethyl]acrylamide |
| Polygonatum odoratum | Octadiene |
| Polygonatum odoratum | ophipogonin D_qt |
| Polygonatum odoratum | 4-[(1R,3aS,4R,6aS)-4-(4-hydroxy-3,5-dimethoxyphenyl)-1,3,3a,4,6,6a-hexahydrofuro[4,3-c]furan-1-yl]-2,6-dimethoxyphenol |
| Polygonatum odoratum | hexanal |
| Polygonatum odoratum | PENTYLFURAN |
| Polygonatum odoratum | (S)-camphor |
| Polygonatum odoratum | palmitic acid |
| Polygonatum odoratum | Cedrol |
| Polygonatum odoratum | 2-heptanone |
| Polygonatum odoratum | HMF |
| Polygonatum odoratum | Linoleic |
| Polygonatum odoratum | Hexyl formate |
| Polygonatum odoratum | liriodendrin_qt |
| Polygonatum odoratum | Vinyl amyl ketone |
