# Supplementary material for: Deciphering potential pharmacological mechanism of Sha-Shen-Mai-Dong decoction on primary Sjogren’s syndrome
Source: BMC Complement Med Ther. 2021 Mar 1;21:79. doi: 10.1186/s12906-021-03257-7 (PMC7923330; doi:10.1186/s12906-021-03257-7)
Supplement: Supplementary file 2 — Additional file 2: Table S2. Relationship between effective compounds and corresponding targets of SSMD. [file 12906_2021_3257_MOESM2_ESM.doc]

**Supplement Table S2 Relationship between effective compounds and corresponding targets of SSMD.**

88 targets associated with 292 unique components

| **Compound** | Target | **Compound** | Target |
| --- | --- | --- | --- |
| Lupiwighteone | EP300 | isorhamnetin | BCKDHA |
| 8-Prenylwighteone | EP300 | rutin | BCKDHA |
| euchrenone | EP300 | Calycosin | BCKDHA |
| (E)-1-(2,4-dihydroxyphenyl)-3-(2,2-dimethylchromen-6-yl)prop-2-en-1-one | EP300 | kaempferol | BCKDHA |
| licoagropin | EP300 | naringenin | BCKDHA |
| (2S)-6-(2,4-dihydroxyphenyl)-2-(2-hydroxypropan-2-yl)-4-methoxy-2,3-dihydrofuro[3,2-g]chromen-7-one | EP300 | beta-Terpinene | BCKDHA |
| Glepidotin A | EP300 | euchrenone | BCKDHA |
| Glepidotin B | EP300 | 3-(2-hydroxy-4-methoxyphenyl)-2H-chromen-7-ol | BCKDHA |
| apioglycyrrhizin | EP300 | Glyzaglabrin | BCKDHA |
| Licoflavonol | EP300 | uralenneoside | BCKDHA |
| Gancaonin P | EP300 | Nortangeretin | BCKDHA |
| Gancaonin R | EP300 | neoisoliquiritin | BCKDHA |
| Gancaonin V | EP300 | 7,2',4'-Trihydroxy-5-methoxy-3-phenylcoumarin | BCKDHA |
| Licoricidin | EP300 | Vestitol | BCKDHA |
| Glycycoumarin | EP300 | Odoratin | BCKDHA |
| Glycyrin | EP300 | quercetin | BCKDHA |
| Licocoumarone | EP300 | Isotrifolin | BCKDHA |
| Licoisoflavone B | EP300 | Tetramethoxyluteolin | BCKDHA |
| (E)-3-[3,4-dihydroxy-5-(3-methylbut-2-enyl)phenyl]-1-(2,4-dihydroxyphenyl)prop-2-en-1-one | EP300 | Skimmin (8CI) | BCKDHA |
| licopyranocoumarin | EP300 | CIR | BCKDHA |
| Glyzaglabrin | EP300 | HMF | BCKDHA |
| Glabrone | EP300 | Moracin G | TXNRD1 |
| Pentadecanol | EP300 | n-coumaroyltyramine | TXNRD1 |
| isoglycycoumarin | EP300 | m-xylene | HBB |
| (Z)-1-(2,4-dihydroxyphenyl)-3-phenylprop-2-en-1-one | EP300 | Morusin | HBB |
| icos-5-enoic acid | EP300 | Izoforon | HBB |
| 7,2',4'-Trihydroxy-5-methoxy-3-phenylcoumarin | EP300 | ISOHEPTANE | HBB |
| Kanzonol H | EP300 | Heptan | HBB |
| gadelaidic acid | EP300 | Pinocembrin | HBB |
| Gancaonin I | EP300 | 7,4'-Dihydroxyflavone | HBB |
| Glycyrrhiza flavonol A | EP300 | formononetin | HBB |
| Corylifolinin | EP300 | Methylheptane | HBB |
| ecdysterone | EP300 | Castanin | HBB |
| Moracin C | EP300 | anethole | HBB |
| Mulberrofuran B | EP300 | 18beta-glycyrrhetinic acid | HBB |
| gynesine | EP300 | glyasperin B | HBB |
| UPL | EP300 | Octadiene | HBB |
| Tetracosane | EP300 | WLN: 4OVR | HBB |
| PENTACOSANE | EP300 | (1S,2S)-1,2-dimethylcyclopentane | HBB |
| Norartocarpetin | EP300 | 2,2-DIMETHYLPENTANE | HBB |
| Linolenic acid ethyl ester | EP300 | liquoric acid | HBB |
| Henicosanoic acid | EP300 | Gancaonin B | HBB |
| morusignin L | EP300 | 2,3-dimethylhexane | HBB |
| PENTADECYLIC ACID | EP300 | (3S)-2,3-dimethylpentane | HBB |
| [(1S)-endo]-(-)-Borneol | EP300 | (4S)-2,4-dimethylhexane | HBB |
| protocatechuic acid | MAOA | (E)-3-[3,4-dihydroxy-5-(3-methylbut-2-enyl)phenyl]-1-(2,4-dihydroxyphenyl)prop-2-en-1-one | HBB |
| nicotiflorin | MAOA | glycyrrhetol | HBB |
| Licorice glycoside A | MAOA | uralsaponin B | HBB |
| Methyleugenol | MAOA | Isohexane | HBB |
| eugenol | MAOA | 3,3-Dimethylpentane | HBB |
| Moracin F | MAOA | 2-Ethyl-p-xylene | HBB |
| Mulberrofuran B | MAOA | 3-methylheptane | HBB |
| Mipax | ADH5 | 3-methylhexane | HBB |
| Inositol | ADH5 | 3-Methylpentane | HBB |
| Medicarpin | TF | Daidzein dimethyl ether | HBB |
| euchrenone | TF | 1-Methoxyficifolinol | HBB |
| glucuronic acid | TF | 5,6,7,8-Tetrahydro-4-methylquinoline | HBB |
| licoisoflavanone | TF | Artonin E | HBB |
| uralenneoside | TF | beta-Glycyrrhetinic acid | HBB |
| gamma-aminobutyric acid | TF | Glycyrrhiza flavonol A | HBB |
| vitamin c | TF | Methyleugenol | HBB |
| Norartocarpetin | TF | eugenol | HBB |
| D-Asparaginsaeure | TF | Mulberrofuran B | HBB |
| GUP | TF | Morindin | HBB |
| (R)-ornithine | TF | Nonanal | HBB |
| Gancaonin B | C5 | kuwanon c | HBB |
| Licoricidin | C5 | Fleet-X | HBB |
| Eurycarpin A | C5 | 1,3,8-p-Menthatriene | HBB |
| Cyclobutanol, 1-ethyl- | C5 | DLA | HBB |
| Inositol | C5 | Terragon | HBB |
| 6-Hydroxycoumarin | C5 | beta-Ionone | HBB |
| 5,7-Dihydroxycoumarin | C5 | Methyl naphthalene | HBB |
| HMF | C5 | (7aR)-4,4,7a-trimethyl-6,7-dihydro-5H-benzofuran-2-one | HBB |
| nicotiflorin | VEGFA | 2-HEXENE | HBB |
| Calycosin | VEGFA | .alpha.-Ionene | HBB |
| kaempferol | VEGFA | Psi-cumene | HBB |
| WLN: 4OVR | VEGFA | 1,2-DIHYDRO-1,5,8-TRIMETHYLNAPHTHALENE | HBB |
| licorice glycoside E | VEGFA | 8-methyltocol | HBB |
| licuraside | VEGFA | poriferast-7-en-3beta-ol | HBB |
| (Z)-1-(2,4-dihydroxyphenyl)-3-phenylprop-2-en-1-one | VEGFA | Schottenol glucoside | HBB |
| Moracin B | VEGFA | 2-Hydroxymethylserine | HBB |
| Moracin F | VEGFA | ()-alpha-Funebrene | HBB |
| Morindin | VEGFA | [(1S)-endo]-(-)-Borneol | HBB |
| Terragon | VEGFA | isorhamnetin | IMPA1 |
| Skimmin (8CI) | VEGFA | kaempferol | IMPA1 |
| neoliquiritin | MMP1 | quercetin | IMPA1 |
| Skimmin (8CI) | MMP1 | D-Asparaginsaeure | IMPA1 |
| protocatechuic acid | BLVRB | ecdysterone | GNB1 |
| Medicarpin | BLVRB | m-xylene | HMGCS1 |
| glucuronic acid | BLVRB | Izoforon | HMGCS1 |
| licoisoflavanone | BLVRB | ISOHEPTANE | HMGCS1 |
| Inositol | BLVRB | Heptan | HMGCS1 |
| vitamin c | BLVRB | Methylheptane | HMGCS1 |
| D-Asparaginsaeure | BLVRB | beta-Terpinene | HMGCS1 |
| GUP | BLVRB | WLN: 4OVR | HMGCS1 |
| (R)-ornithine | BLVRB | (1S,2S)-1,2-dimethylcyclopentane | HMGCS1 |
| Mairin | RHO | apioglycyrrhizin | HMGCS1 |
| 2-Caren-10-al | RHO | 2,2-DIMETHYLPENTANE | HMGCS1 |
| 8-Prenylwighteone | RHO | 2,3-dimethylhexane | HMGCS1 |
| (E)-1-(2,4-dihydroxyphenyl)-3-(2,2-dimethylchromen-6-yl)prop-2-en-1-one | RHO | Prunetin | HMGCS1 |
| Octadiene | RHO | (3S)-2,3-dimethylpentane | HMGCS1 |
| Phaseolinisoflavan | RHO | (4S)-2,4-dimethylhexane | HMGCS1 |
| glabrol | RHO | glycyrrhetol | HMGCS1 |
| liquoric acid | RHO | 3,22-Dihydroxy-11-oxo-delta(12)-oleanene-27-alpha-methoxycarbonyl-29-oic acid | HMGCS1 |
| Gancaonin C | RHO | Isohexane | HMGCS1 |
| Gancaonin U | RHO | 3,3-Dimethylpentane | HMGCS1 |
| Gancaonin V | RHO | 3-methylheptane | HMGCS1 |
| Glabridin | RHO | 3-methylhexane | HMGCS1 |
| Docosyl caffeate | RHO | 3-Methylpentane | HMGCS1 |
| Nortangeretin | RHO | 5,6,7,8-Tetrahydro-4-methylquinoline | HMGCS1 |
| HMO | RHO | Licoriisoflavan A | HMGCS1 |
| 24-Hydroxyglycyrrhetic acid | RHO | poriferast-5-en-3beta-ol | HMGCS1 |
| 3'-Hydroxy-4'-O-Methylglabridin | RHO | Methyleugenol | HMGCS1 |
| licochalcone a | RHO | eugenol | HMGCS1 |
| 3'-Methoxyglabridin | RHO | 1-hexanol | HMGCS1 |
| 1-(5-hydroxy-2,2-dimethylchromen-6-yl)-3-(4-hydroxyphenyl)prop-2-en-1-one | RHO | Fleet-X | HMGCS1 |
| PENTYLFURAN | RHO | hexanoic acid | HMGCS1 |
| Albafuran A | RHO | Terragon | HMGCS1 |
| Isobutyrylmallotochromene | RHO | beta-Ionone | HMGCS1 |
| Moracin D | RHO | tectorigenin | HMGCS1 |
| Moracin E | RHO | 2,6-Dimethyl-1,3,5,7-octatetraene, E,E- | HMGCS1 |
| Moracin G | RHO | Methyl naphthalene | HMGCS1 |
| Moracin H | RHO | (7aR)-4,4,7a-trimethyl-6,7-dihydro-5H-benzofuran-2-one | HMGCS1 |
| Nonanal | RHO | 2-HEXENE | HMGCS1 |
| UPL | RHO | .alpha.-Ionene | HMGCS1 |
| Tetracosane | RHO | Psi-cumene | HMGCS1 |
| DEP | RHO | 8-methyltocol | HMGCS1 |
| beta-Ionone | RHO | gamma-Hexenol | HMGCS1 |
| PENTACOSANE | RHO | poriferast-7-en-3beta-ol | HMGCS1 |
| 2,6-Dimethyl-1,3,5,7-octatetraene, E,E- | RHO | 2-Hydroxymethylserine | HMGCS1 |
| .alpha.-Ionene | RHO | ()-alpha-Funebrene | HMGCS1 |
| Henicosanoic acid | RHO | [(1S)-endo]-(-)-Borneol | HMGCS1 |
| Hex-3-enyl acetate | RHO | 7,4'-Dihydroxyflavone | ALB |
| methyl 8-methyl-decanoate | RHO | Calycosin | ALB |
| bergamotene (Z,.alpha.,cis) | RHO | kaempferol | ALB |
| poriferast-7-en-3beta-ol | RHO | Castanin | ALB |
| Schottenol glucoside | RHO | beta-Terpinene | ALB |
| 2-Hydroxymethylserine | RHO | 3-Hydroxyglabrol | ALB |
| ()-alpha-Funebrene | RHO | Glepidotin B | ALB |
| [(1S)-endo]-(-)-Borneol | RHO | Karenzu DK2 | ALB |
| n-coumaroyltyramine | RHO | Licoflavonol | ALB |
| DFV | CD1B | Licoricone | ALB |
| Mairin | CD1B | Gancaonin A | ALB |
| oleanolic acid | CD1B | Gancaonin B | ALB |
| nicotiflorin | CD1B | Gancaonin P | ALB |
| butylated hydroxytoluene | CD1B | liquiritin | ALB |
| Lupiwighteone | CD1B | Glyzaglabrin | ALB |
| Calycosin | CD1B | Eurycarpin A | ALB |
| (E)-1-butoxyhex-2-ene | CD1B | vitexin | ALB |
| 3-Hydroxyglabrol | CD1B | Nortangeretin | ALB |
| Isotrifoliol | CD1B | HMO | ALB |
| (2S)-6-(2,4-dihydroxyphenyl)-2-(2-hydroxypropan-2-yl)-4-methoxy-2,3-dihydrofuro[3,2-g]chromen-7-one | CD1B | Gancaonin G | ALB |
| Glypallichalcone | CD1B | Gancaonin H | ALB |
| echinatin | CD1B | Corylifolinin | ALB |
| Gancaonin S | CD1B | Odoratin | ALB |
| shinpterocarpin | CD1B | quercetin | ALB |
| licopyranocoumarin | CD1B | 4-Prenylresveratrol | ALB |
| Glabranin | CD1B | beta-Ionone | ALB |
| Glabrene | CD1B | tectorigenin | ALB |
| 2-methyl-6-ethyl decane | CD1B | (7aR)-4,4,7a-trimethyl-6,7-dihydro-5H-benzofuran-2-one | ALB |
| (E)-dodec-2-ene | CD1B | Tetramethoxyluteolin | ALB |
| Cyclobutanol, 1-ethyl- | CD1B | methyl 8-methyl-decanoate | ALB |
| isoglycycoumarin | CD1B | Elatericin A | ALB |
| 1-Methoxyphaseollidin | CD1B | HMF | ALB |
| 1-Methoxyficifolinol | CD1B | m-xylene | TRIO |
| 7,2',4'-Trihydroxy-5-methoxy-3-phenylcoumarin | CD1B | Izoforon | TRIO |
| Licoagrocarpin | CD1B | Heptan | TRIO |
| Corylifolinin | CD1B | (2S)-6-(2,4-dihydroxyphenyl)-2-(2-hydroxypropan-2-yl)-4-methoxy-2,3-dihydrofuro[3,2-g]chromen-7-one | TRIO |
| Odoratin | CD1B | 3-(2-hydroxy-4-methoxyphenyl)-2H-chromen-7-ol | TRIO |
| Phaseol | CD1B | Glypallichalcone | TRIO |
| DIBP | CD1B | echinatin | TRIO |
| PENTYLFURAN | CD1B | Licochalcone B | TRIO |
| Methyleugenol | CD1B | 2,3-dimethylhexane | TRIO |
| Moracin B | CD1B | (4S)-2,4-dimethylhexane | TRIO |
| Moracin F | CD1B | Glyzaglabrin | TRIO |
| Mulberrofuran B | CD1B | 3,4,3',4'-Tetrahydroxy-2-methoxychalcone | TRIO |
| [(3S)-3,7-dimethylocta-1,6-dien-3-yl] propanoate | CD1B | 3-methylhexane | TRIO |
| 1-Tetradecene | CD1B | 7,2',4'-Trihydroxy-5-methoxy-3-phenylcoumarin | TRIO |
| Quercimeritrin (6CI,7CI,8CI) | CD1B | DIBP | TRIO |
| (4S)-1-methyl-4-(6-methylhepta-1,5-dien-2-yl)cyclohexene | CD1B | Fleet-X | TRIO |
| 2,6,11-trimethyldodecane | CD1B | beta-Ionone | TRIO |
| Linolenic acid ethyl ester | CD1B | Inositol | NOS1 |
| Hex-3-enyl acetate | CD1B | Azeton | NOS1 |
| 8-methyltocol | CD1B | Morusin | MAPK9 |
| Nerylacetone | CD1B | Lupiwighteone | MAPK9 |
| Elatericin A | CD1B | glyasperin B | MAPK9 |
| 7-TETRADECENE | CD1B | (2S)-6-(2,4-dihydroxyphenyl)-2-(2-hydroxypropan-2-yl)-4-methoxy-2,3-dihydrofuro[3,2-g]chromen-7-one | MAPK9 |
| PENTADECYLIC ACID | CD1B | Phaseolinisoflavan | MAPK9 |
| Yamogenin | CD1B | 3-(2-hydroxy-4-methoxyphenyl)-2H-chromen-7-ol | MAPK9 |
| 18beta-glycyrrhetinic acid | ACADM | liquoric acid | MAPK9 |
| Gancaonin P | ACADM | Licoricone | MAPK9 |
| Gancaonin Q | ACADM | licorice glycoside E | MAPK9 |
| Gancaonin U | ACADM | Glycycoumarin | MAPK9 |
| glycyrrhetol | ACADM | Glycyrin | MAPK9 |
| liquiritin | ACADM | 5,6,7,8-Tetrahydro-2,4-dimethylquinoline | MAPK9 |
| Eurycarpin A | ACADM | Licoisoflavone B | MAPK9 |
| (-)-Medicocarpin | ACADM | shinpterocarpin | MAPK9 |
| vitexin | ACADM | (E)-3-[3,4-dihydroxy-5-(3-methylbut-2-enyl)phenyl]-1-(2,4-dihydroxyphenyl)prop-2-en-1-one | MAPK9 |
| 4H-1-Benzopyran-4-one, 2-(4-(beta-D-glucopyranosyloxy)phenyl)-2,3-dihydro-5,7-dihydroxy-, (2S)- | ACADM | licopyranocoumarin | MAPK9 |
| Uralenol-3-methylether | ACADM | Hispaglabridin B | MAPK9 |
| Liquiritin apioside | ACADM | Glyzaglabrin | MAPK9 |
| 6鈥?O-acetylliquiritin | ACADM | Glabridin | MAPK9 |
| beta-Glycyrrhetinic acid | ACADM | Glabrone | MAPK9 |
| Astragalin | ACADM | Eurycarpin A | MAPK9 |
| Albafuran A | ACADM | (-)-Medicocarpin | MAPK9 |
| Isotrifolin | ACADM | neoliquiritin | MAPK9 |
| ISOHEPTANE | IL10 | Cyclobutanol, 1-ethyl- | MAPK9 |
| Medicarpin | IL10 | isoglycycoumarin | MAPK9 |
| Glepidotin B | IL10 | (Z)-1-(2,4-dihydroxyphenyl)-3-phenylprop-2-en-1-one | MAPK9 |
| (1S,2S)-1,2-dimethylcyclopentane | IL10 | 3'-Hydroxy-4'-O-Methylglabridin | MAPK9 |
| 2,2-DIMETHYLPENTANE | IL10 | 3'-Methoxyglabridin | MAPK9 |
| (3S)-2,3-dimethylpentane | IL10 | 1-Methoxyficifolinol | MAPK9 |
| Isohexane | IL10 | 5,6,7,8-Tetrahydro-4-methylquinoline | MAPK9 |
| neoisoliquiritin | IL10 | 7,2',4'-Trihydroxy-5-methoxy-3-phenylcoumarin | MAPK9 |
| Isoschaftoside | IL10 | Vestitol | MAPK9 |
| 3,3-Dimethylpentane | IL10 | Gancaonin I | MAPK9 |
| 3-methylheptane | IL10 | Licoagroisoflavone | MAPK9 |
| 3-Methylpentane | IL10 | PENTYLFURAN | MAPK9 |
| 5,6,7,8-Tetrahydro-4-methylquinoline | IL10 | ecdysterone | MAPK9 |
| Chlorogenic acid | IL10 | eugenol | MAPK9 |
| Methyl naphthalene | IL10 | Insularine | MAPK9 |
| 2-HEXENE | IL10 | Majudin | MAPK9 |
| (R)-ornithine | IL10 | hexanoic acid | MAPK9 |
| HMF | IL10 | Damascenone | MAPK9 |
| Narcissoside | PPT1 | Norartocarpetin | MAPK9 |
| formononetin | PPT1 | Hex-3-enyl acetate | MAPK9 |
| Karenzu DK2 | PPT1 | I-Amyl acetate | MAPK9 |
| Gancaonin R | PPT1 | Isoamyl benzoate | MAPK9 |
| Gancaonin S | PPT1 | 8-methyltocol | MAPK9 |
| Gancaonin V | PPT1 | Leaf acetate | MAPK9 |
| (E)-3-[3,4-dihydroxy-5-(3-methylbut-2-enyl)phenyl]-1-(2,4-dihydroxyphenyl)prop-2-en-1-one | PPT1 | morusignin L | MAPK9 |
| Isoviolanthin | PPT1 | Skimmin (8CI) | MAPK9 |
| 1-Methoxyficifolinol | PPT1 | (2R,3R,4S,5S,6R)-2-[[(3S,5S,9R,10S,13R,14R,17R)-17-[(E,2R,5S)-5-ethyl-6-methylhept-3-en-2-yl]-10,13-dimethyl-2,3,4,5,6,9,11,12,14,15,16,17-dodecahydro-1H-cyclopenta[a]phenanthren-3-yl]oxy]-6-(hydroxymethyl)oxane-3,4,5-triol | MAPK9 |
| 4,2',4',alpha-Tetrahydroxydihydrochalcone | PPT1 | (S)-camphor | MAPK9 |
| 4-Prenylresveratrol | PPT1 | Fleet-X | TLR4 |
| Isotrifolin | PPT1 | CIR | CYP2C8 |
| hexanoic acid | PPT2 | Pinocembrin | HSD11B1 |
| DFV | MAOB | butylated hydroxytoluene | HSD11B1 |
| butylated hydroxytoluene | MAOB | 7,4'-Dihydroxyflavone | HSD11B1 |
| Glyasperin C | MAOB | Castanin | HSD11B1 |
| glabrol | MAOB | (E)-1-butoxyhex-2-ene | HSD11B1 |
| liquoric acid | MAOB | liquoric acid | HSD11B1 |
| Licoricidin | MAOB | Gancaonin R | HSD11B1 |
| Glabranin | MAOB | Gancaonin V | HSD11B1 |
| 2-methyl-6-ethyl decane | MAOB | 2-methyl-6-ethyl decane | HSD11B1 |
| Pentadecanol | MAOB | Pentadecanol | HSD11B1 |
| (E)-dodec-2-ene | MAOB | Uralenol-3-methylether | HSD11B1 |
| 2-Tetradecanone | MAOB | (E)-dodec-2-ene | HSD11B1 |
| HMO | MAOB | 2-Tetradecanone | HSD11B1 |
| 1-Methoxyphaseollidin | MAOB | isoglabrolide | HSD11B1 |
| Licoagrocarpin | MAOB | HMO | HSD11B1 |
| Moracin E | MAOB | 2,6,10-trimethyl-dodecane | HSD11B1 |
| linolenic acid | MAOB | 12-methyltetradecanoate | HSD11B1 |
| Heptadekan | MAOB | gadelaidic acid | HSD11B1 |
| 1-Tetradecene | MAOB | Artonin E | HSD11B1 |
| beta-Rhodinol | MAOB | Licoagrocarpin | HSD11B1 |
| (4S)-1-methyl-4-(6-methylhepta-1,5-dien-2-yl)cyclohexene | MAOB | Phaseol | HSD11B1 |
| Henicosanoic acid | MAOB | Isobutyrylmallotochromene | HSD11B1 |
| Linolenic acid methyl ester | MAOB | Moracin D | HSD11B1 |
| bergamotene (Z,.alpha.,cis) | MAOB | Moracin E | HSD11B1 |
| (2R,3R,4S,5S,6R)-2-[[(3S,5S,9R,10S,13R,14R,17R)-17-[(E,2R,5S)-5-ethyl-6-methylhept-3-en-2-yl]-10,13-dimethyl-2,3,4,5,6,9,11,12,14,15,16,17-dodecahydro-1H-cyclopenta[a]phenanthren-3-yl]oxy]-6-(hydroxymethyl)oxane-3,4,5-triol | MAOB | 4-Prenylresveratrol | HSD11B1 |
| DIHYDROCUCURBITACIN B | MAOB | linolenic acid | HSD11B1 |
| 7-TETRADECENE | MAOB | Heptadekan | HSD11B1 |
| protocatechuic acid | PYGL | Ethylpalmitate | HSD11B1 |
| m-xylene | PYGL | Oktadekan | HSD11B1 |
| Izoforon | PYGL | FITONE | HSD11B1 |
| Heptan | PYGL | 1-Tetradecene | HSD11B1 |
| Medicarpin | PYGL | (4S)-1-methyl-4-(6-methylhepta-1,5-dien-2-yl)cyclohexene | HSD11B1 |
| nicotiflorin | PYGL | Henicosanoic acid | HSD11B1 |
| Narcissoside | PYGL | Linolenic acid methyl ester | HSD11B1 |
| Hirsutrin | PYGL | bergamotene (Z,.alpha.,cis) | HSD11B1 |
| 8-Prenylwighteone | PYGL | 2-Methylnonan-3-one | HSD11B1 |
| Methylheptane | PYGL | 7-TETRADECENE | HSD11B1 |
| glyasperin B | PYGL | [(1S)-endo]-(-)-Borneol | HSD11B1 |
| (E)-1-(2,4-dihydroxyphenyl)-3-(2,2-dimethylchromen-6-yl)prop-2-en-1-one | PYGL | Cyclobutanol, 1-ethyl- | AKR1C3 |
| licoagropin | PYGL | 5,6,7,8-Tetrahydro-4-methylquinoline | AKR1C3 |
| (2S)-6-(2,4-dihydroxyphenyl)-2-(2-hydroxypropan-2-yl)-4-methoxy-2,3-dihydrofuro[3,2-g]chromen-7-one | PYGL | Glepidotin A | F3 |
| Glypallichalcone | PYGL | HMO | F3 |
| echinatin | PYGL | Heptan | AR |
| licochalcone G | PYGL | oleanolic acid | AR |
| liquoric acid | PYGL | nicotiflorin | AR |
| 2,3-dimethylhexane | PYGL | Pinocembrin | AR |
| Gancaonin U | PYGL | Lupiwighteone | AR |
| (E)-1-[2,4-dihydroxy-3-(3-methylbut-2-enyl)phenyl]-3-(2,4-dihydroxyphenyl)prop-2-en-1-one | PYGL | 7,4'-Dihydroxyflavone | AR |
| licoisoflavanone | PYGL | formononetin | AR |
| (4S)-2,4-dimethylhexane | PYGL | Calycosin | AR |
| shinpterocarpin | PYGL | naringenin | AR |
| Hispaglabridin B | PYGL | 8-Prenylwighteone | AR |
| Glyzaglabrin | PYGL | Methylheptane | AR |
| Glabridin | PYGL | Castanin | AR |
| Glabrone | PYGL | (E)-1-butoxyhex-2-ene | AR |
| vitexin | PYGL | 3-Hydroxyglabrol | AR |
| Pentadecanol | PYGL | glyasperin B | AR |
| uralenneoside | PYGL | Glyasperin C | AR |
| Isoschaftoside | PYGL | Isotrifoliol | AR |
| 1-Methoxyphaseollidin | PYGL | (E)-1-(2,4-dihydroxyphenyl)-3-(2,2-dimethylchromen-6-yl)prop-2-en-1-one | AR |
| (Z)-1-(2,4-dihydroxyphenyl)-3-phenylprop-2-en-1-one | PYGL | (2R)-1-[2,4-dihydroxy-5-(3-methylbut-2-enyl)phenyl]-2-hydroxy-3-[4-hydroxy-3-(3-methylbut-2-enyl)phenyl]propan-1-one | AR |
| 3-methylhexane | PYGL | licoagropin | AR |
| 4,2',4',alpha-Tetrahydroxydihydrochalcone | PYGL | Octadiene | AR |
| Astragalin | PYGL | (E)-1-[2,4-dihydroxy-3-(3-methylbut-2-enyl)phenyl]-3-[4-hydroxy-3-(3-methylbut-2-enyl)phenyl]prop-2-en-1-one | AR |
| DIBP | PYGL | licochalcone C | AR |
| Insularine | PYGL | glabrol | AR |
| Moracin G | PYGL | apioglycyrrhizin | AR |
| Moracin H | PYGL | licochalcone G | AR |
| 5-Hydroxycoumarin | PYGL | Licoricone | AR |
| FITONE | PYGL | Gancaonin A | AR |
| Nonadecene | PYGL | Gancaonin B | AR |
| vitamin c | PYGL | Gancaonin C | AR |
| Fleet-X | PYGL | 2,3-dimethylhexane | AR |
| beta-Ionone | PYGL | Prunetin | AR |
| Isotrifolin | PYGL | Gancaonin D | AR |
| Linolenyl alcohol | PYGL | Gancaonin Q | AR |
| Norartocarpetin | PYGL | Gancaonin R | AR |
| D-Asparaginsaeure | PYGL | (E)-1-[2,4-dihydroxy-3-(3-methylbut-2-enyl)phenyl]-3-(2,4-dihydroxyphenyl)prop-2-en-1-one | AR |
| 5,7-Dihydroxycoumarin | PYGL | Licoisoflavone B | AR |
| Elatericin A | PYGL | (E)-3-[3,4-dihydroxy-5-(3-methylbut-2-enyl)phenyl]-1-(2,4-dihydroxyphenyl)prop-2-en-1-one | AR |
| Spinasterol | PYGL | glycyrrhetol | AR |
| GUP | PYGL | liquiritin | AR |
| DIHYDROCUCURBITACIN B | PYGL | 3,22-Dihydroxy-11-oxo-delta(12)-oleanene-27-alpha-methoxycarbonyl-29-oic acid | AR |
| (R)-ornithine | PYGL | Hispaglabridin B | AR |
| [(1S)-endo]-(-)-Borneol | PYGL | Glyzaglabrin | AR |
| n-coumaroyltyramine | PYGL | Glabridin | AR |
| (S)-camphor | PYGL | Glabrone | AR |
| Medicarpin | IGFBP1 | Docosyl caffeate | AR |
| Morusin | PMP2 | vitexin | AR |
| DFV | PMP2 | 4H-1-Benzopyran-4-one, 2-(4-(beta-D-glucopyranosyloxy)phenyl)-2,3-dihydro-5,7-dihydroxy-, (2S)- | AR |
| oleanolic acid | PMP2 | Pentadecanol | AR |
| nicotiflorin | PMP2 | Uralenol-3-methylether | AR |
| Pinocembrin | PMP2 | Nortangeretin | AR |
| isorhamnetin | PMP2 | 2-Tetradecanone | AR |
| Lupiwighteone | PMP2 | 24-Hydroxyglycyrrhetic acid | AR |
| 7,4'-Dihydroxyflavone | PMP2 | 3'-Hydroxy-4'-O-Methylglabridin | AR |
| Narcissoside | PMP2 | 3-methylhexane | AR |
| formononetin | PMP2 | 3'-Methoxyglabridin | AR |
| rutin | PMP2 | Daidzein dimethyl ether | AR |
| Calycosin | PMP2 | 1-(5-hydroxy-2,2-dimethylchromen-6-yl)-3-(4-hydroxyphenyl)prop-2-en-1-one | AR |
| naringenin | PMP2 | Licoriisoflavan A | AR |
| Hirsutrin | PMP2 | icos-5-enoic acid | AR |
| 8-Prenylwighteone | PMP2 | 6鈥?O-acetylliquiritin | AR |
| Castanin | PMP2 | Kanzonol H | AR |
| beta-Terpinene | PMP2 | gadelaidic acid | AR |
| anethole | PMP2 | Gancaonin H | AR |
| (E)-1-butoxyhex-2-ene | PMP2 | Glycyrrhiza flavonol A | AR |
| 3-Hydroxyglabrol | PMP2 | Corylifolinin | AR |
| 18beta-glycyrrhetinic acid | PMP2 | Kanzonol E | AR |
| euchrenone | PMP2 | Odoratin | AR |
| glyasperin B | PMP2 | Phaseol | AR |
| glyasperin E | PMP2 | Xambioona | AR |
| Isotrifoliol | PMP2 | PENTYLFURAN | AR |
| (E)-1-(2,4-dihydroxyphenyl)-3-(2,2-dimethylchromen-6-yl)prop-2-en-1-one | PMP2 | poriferast-5-en-3beta-ol | AR |
| (2R)-1-[2,4-dihydroxy-5-(3-methylbut-2-enyl)phenyl]-2-hydroxy-3-[4-hydroxy-3-(3-methylbut-2-enyl)phenyl]propan-1-one | PMP2 | ecdysterone | AR |
| licoagropin | PMP2 | eugenol | AR |
| (2S)-6-(2,4-dihydroxyphenyl)-2-(2-hydroxypropan-2-yl)-4-methoxy-2,3-dihydrofuro[3,2-g]chromen-7-one | PMP2 | Albafuran A | AR |
| Glepidotin A | PMP2 | Insularine | AR |
| Glepidotin B | PMP2 | Isobutyrylmallotochromene | AR |
| (E)-1-[2,4-dihydroxy-3-(3-methylbut-2-enyl)phenyl]-3-[4-hydroxy-3-(3-methylbut-2-enyl)phenyl]prop-2-en-1-one | PMP2 | Moracin B | AR |
| WLN: 4OVR | PMP2 | Moracin C | AR |
| Karenzu DK2 | PMP2 | Moracin D | AR |
| licochalcone C | PMP2 | Moracin E | AR |
| glabrol | PMP2 | Moracin F | AR |
| apioglycyrrhizin | PMP2 | Mulberrofuran B | AR |
| licochalcone G | PMP2 | gynesine | AR |
| Licoflavonol | PMP2 | Morindin | AR |
| Licoricone | PMP2 | linolenic acid | AR |
| Gancaonin A | PMP2 | Heptadekan | AR |
| Gancaonin B | PMP2 | Ethylpalmitate | AR |
| Gancaonin C | PMP2 | Oktadekan | AR |
| Prunetin | PMP2 | TWT | AR |
| licorice glycoside E | PMP2 | FITONE | AR |
| Gancaonin D | PMP2 | Nonadecene | AR |
| Gancaonin P | PMP2 | UPL | AR |
| Gancaonin Q | PMP2 | Tetracosane | AR |
| Gancaonin R | PMP2 | DLA | AR |
| Gancaonin S | PMP2 | PENTACOSANE | AR |
| Gancaonin U | PMP2 | tectorigenin | AR |
| Gancaonin V | PMP2 | 2,6-Dimethyl-1,3,5,7-octatetraene, E,E- | AR |
| Licoricidin | PMP2 | .alpha.-Ionene | AR |
| Glycycoumarin | PMP2 | Octadecanal | AR |
| Glycyrin | PMP2 | Linolenyl alcohol | AR |
| (E)-1-[2,4-dihydroxy-3-(3-methylbut-2-enyl)phenyl]-3-(2,4-dihydroxyphenyl)prop-2-en-1-one | PMP2 | Linolenic acid ethyl ester | AR |
| Licocoumarone | PMP2 | 1,2-DIHYDRO-1,5,8-TRIMETHYLNAPHTHALENE | AR |
| Licoisoflavone B | PMP2 | Tetramethoxyluteolin | AR |
| shinpterocarpin | PMP2 | Henicosanoic acid | AR |
| (E)-3-[3,4-dihydroxy-5-(3-methylbut-2-enyl)phenyl]-1-(2,4-dihydroxyphenyl)prop-2-en-1-one | PMP2 | 8-methyltocol | AR |
| glycyrrhetol | PMP2 | Linolenic acid methyl ester | AR |
| liquiritin | PMP2 | bergamotene (Z,.alpha.,cis) | AR |
| licopyranocoumarin | PMP2 | 5,7-Dihydroxycoumarin | AR |
| Glyzaglabrin | PMP2 | morusignin L | AR |
| Glabranin | PMP2 | cucurbitacin b | AR |
| Eurycarpin A | PMP2 | Elatericin A | AR |
| Docosyl caffeate | PMP2 | Spinasterol | AR |
| (-)-Medicocarpin | PMP2 | (2R,3R,4S,5S,6R)-2-[[(3S,5S,9R,10S,13R,14R,17R)-17-[(E,2R,5S)-5-ethyl-6-methylhept-3-en-2-yl]-10,13-dimethyl-2,3,4,5,6,9,11,12,14,15,16,17-dodecahydro-1H-cyclopenta[a]phenanthren-3-yl]oxy]-6-(hydroxymethyl)oxane-3,4,5-triol | AR |
| vitexin | PMP2 | DIHYDROCUCURBITACIN B | AR |
| 4H-1-Benzopyran-4-one, 2-(4-(beta-D-glucopyranosyloxy)phenyl)-2,3-dihydro-5,7-dihydroxy-, (2S)- | PMP2 | poriferast-7-en-3beta-ol | AR |
| violanthin | PMP2 | Schottenol glucoside | AR |
| Uralenol | PMP2 | 2-Hydroxymethylserine | AR |
| Uralenol-3-methylether | PMP2 | PENTADECYLIC ACID | AR |
| uralenneoside | PMP2 | Yamogenin | AR |
| Nortangeretin | PMP2 | (S)-camphor | AR |
| neoliquiritin | PMP2 | Heptan | GAD1 |
| neoisoliquiritin | PMP2 | Pinocembrin | GAD1 |
| Isoviolanthin | PMP2 | formononetin | GAD1 |
| isoglycycoumarin | PMP2 | 2-Caren-10-al | GAD1 |
| licuraside | PMP2 | 8-Prenylwighteone | GAD1 |
| Liquiritin apioside | PMP2 | Methylheptane | GAD1 |
| isoglabrolide | PMP2 | 3-Hydroxyglabrol | GAD1 |
| HMO | PMP2 | euchrenone | GAD1 |
| Isoschaftoside | PMP2 | glyasperin E | GAD1 |
| 1-Methoxyphaseollidin | PMP2 | Glyasperin C | GAD1 |
| 24-Hydroxyglycyrrhetic acid | PMP2 | Isotrifoliol | GAD1 |
| (Z)-1-(2,4-dihydroxyphenyl)-3-phenylprop-2-en-1-one | PMP2 | (E)-1-(2,4-dihydroxyphenyl)-3-(2,2-dimethylchromen-6-yl)prop-2-en-1-one | GAD1 |
| 3,4,3',4'-Tetrahydroxy-2-methoxychalcone | PMP2 | licoagropin | GAD1 |
| licochalcone a | PMP2 | Octadiene | GAD1 |
| Daidzein dimethyl ether | PMP2 | (E)-1-[2,4-dihydroxy-3-(3-methylbut-2-enyl)phenyl]-3-[4-hydroxy-3-(3-methylbut-2-enyl)phenyl]prop-2-en-1-one | GAD1 |
| 1-Methoxyficifolinol | PMP2 | Phaseolinisoflavan | GAD1 |
| 4,2',4',alpha-Tetrahydroxydihydrochalcone | PMP2 | Glypallichalcone | GAD1 |
| 1-(5-hydroxy-2,2-dimethylchromen-6-yl)-3-(4-hydroxyphenyl)prop-2-en-1-one | PMP2 | echinatin | GAD1 |
| 6鈥?O-acetylliquiritin | PMP2 | Karenzu DK2 | GAD1 |
| 7,2',4'-Trihydroxy-5-methoxy-3-phenylcoumarin | PMP2 | Licochalcone B | GAD1 |
| 7-Acetoxy-2-methylisoflavone | PMP2 | licochalcone C | GAD1 |
| Kanzonol H | PMP2 | licochalcone G | GAD1 |
| Artonin E | PMP2 | Gancaonin B | GAD1 |
| Gancaonin G | PMP2 | Gancaonin C | GAD1 |
| Gancaonin H | PMP2 | 2,3-dimethylhexane | GAD1 |
| beta-Glycyrrhetinic acid | PMP2 | Gancaonin D | GAD1 |
| Licoagrocarpin | PMP2 | Gancaonin R | GAD1 |
| Gancaonin I | PMP2 | Gancaonin S | GAD1 |
| Glyasperin A | PMP2 | Licoricidin | GAD1 |
| Glycyrrhiza flavonol A | PMP2 | (E)-1-[2,4-dihydroxy-3-(3-methylbut-2-enyl)phenyl]-3-(2,4-dihydroxyphenyl)prop-2-en-1-one | GAD1 |
| Corylifolinin | PMP2 | Licoisoflavone B | GAD1 |
| Kanzonol E | PMP2 | (E)-3-[3,4-dihydroxy-5-(3-methylbut-2-enyl)phenyl]-1-(2,4-dihydroxyphenyl)prop-2-en-1-one | GAD1 |
| Licoagroisoflavone | PMP2 | glycyrrhetol | GAD1 |
| Licorice glycoside A | PMP2 | Hispaglabridin B | GAD1 |
| Odoratin | PMP2 | Glabridin | GAD1 |
| Phaseol | PMP2 | Eurycarpin A | GAD1 |
| Xambioona | PMP2 | Docosyl caffeate | GAD1 |
| Mipax | PMP2 | Pentadecanol | GAD1 |
| Astragalin | PMP2 | Nortangeretin | GAD1 |
| DIBP | PMP2 | 24-Hydroxyglycyrrhetic acid | GAD1 |
| quercetin | PMP2 | 3'-Hydroxy-4'-O-Methylglabridin | GAD1 |
| Methyleugenol | PMP2 | 3,4,3',4'-Tetrahydroxy-2-methoxychalcone | GAD1 |
| ecdysterone | PMP2 | 3-methylhexane | GAD1 |
| eugenol | PMP2 | 3'-Methoxyglabridin | GAD1 |
| Albafuran A | PMP2 | 4,2',4',alpha-Tetrahydroxydihydrochalcone | GAD1 |
| Insularine | PMP2 | 1-(5-hydroxy-2,2-dimethylchromen-6-yl)-3-(4-hydroxyphenyl)prop-2-en-1-one | GAD1 |
| Isobutyrylmallotochromene | PMP2 | icos-5-enoic acid | GAD1 |
| Moracin B | PMP2 | Kanzonol H | GAD1 |
| Moracin C | PMP2 | Gancaonin H | GAD1 |
| Moracin E | PMP2 | Corylifolinin | GAD1 |
| Moracin F | PMP2 | DIBP | GAD1 |
| Moracin H | PMP2 | 2-heptanone | GAD1 |
| Mulberrofuran B | PMP2 | WLN: VH6 | GAD1 |
| gynesine | PMP2 | PCR | GAD1 |
| Chlorogenic acid | PMP2 | Albafuran A | GAD1 |
| Morindin | PMP2 | Isobutyrylmallotochromene | GAD1 |
| Nonanal | PMP2 | Moracin B | GAD1 |
| kuwanon c | PMP2 | Moracin E | GAD1 |
| FITONE | PMP2 | Moracin G | GAD1 |
| [(3S)-3,7-dimethylocta-1,6-dien-3-yl] propanoate | PMP2 | Moracin H | GAD1 |
| Quercimeritrin (6CI,7CI,8CI) | PMP2 | 1-hexanol | GAD1 |
| DEP | PMP2 | kuwanon c | GAD1 |
| DLA | PMP2 | Ethylpalmitate | GAD1 |
| Terragon | PMP2 | TWT | GAD1 |
| beta-Ionone | PMP2 | Nonadecene | GAD1 |
| beta-Rhodinol | PMP2 | UPL | GAD1 |
| tectorigenin | PMP2 | Tetracosane | GAD1 |
| Isotrifolin | PMP2 | PENTACOSANE | GAD1 |
| (7aR)-4,4,7a-trimethyl-6,7-dihydro-5H-benzofuran-2-one | PMP2 | PTL | GAD1 |
| Norartocarpetin | PMP2 | Sulcatone | GAD1 |
| Tetramethoxyluteolin | PMP2 | beta-Rhodinol | GAD1 |
| methyl 8-methyl-decanoate | PMP2 | WLN: QV4 | GAD1 |
| 8-methyltocol | PMP2 | Ethyl caffeate | GAD1 |
| Nerylacetone | PMP2 | Amylol | GAD1 |
| morusignin L | PMP2 | 2,6-Dimethyl-1,3,5,7-octatetraene, E,E- | GAD1 |
| Skimmin (8CI) | PMP2 | (7aR)-4,4,7a-trimethyl-6,7-dihydro-5H-benzofuran-2-one | GAD1 |
| cucurbitacin b | PMP2 | Octadecanal | GAD1 |
| Elatericin A | PMP2 | Linolenyl alcohol | GAD1 |
| (2R,3R,4S,5S,6R)-2-[[(3S,5S,9R,10S,13R,14R,17R)-17-[(E,2R,5S)-5-ethyl-6-methylhept-3-en-2-yl]-10,13-dimethyl-2,3,4,5,6,9,11,12,14,15,16,17-dodecahydro-1H-cyclopenta[a]phenanthren-3-yl]oxy]-6-(hydroxymethyl)oxane-3,4,5-triol | PMP2 | Linolenic acid ethyl ester | GAD1 |
| DIHYDROCUCURBITACIN B | PMP2 | Henicosanoic acid | GAD1 |
| Schottenol glucoside | PMP2 | I-Amyl acetate | GAD1 |
| n-coumaroyltyramine | PMP2 | 2-Hexenol | GAD1 |
| Yamogenin | PMP2 | Leaf acetate | GAD1 |
| (S)-camphor | PMP2 | gamma-Hexenol | GAD1 |
| (2S)-6-(2,4-dihydroxyphenyl)-2-(2-hydroxypropan-2-yl)-4-methoxy-2,3-dihydrofuro[3,2-g]chromen-7-one | CES1 | 3,5-Octadien-2-one, (E,E)- | GAD1 |
| butylated hydroxytoluene | APP | Spinasterol | GAD1 |
| licoagropin | APP | poriferast-7-en-3beta-ol | GAD1 |
| 3-(2-hydroxy-4-methoxyphenyl)-2H-chromen-7-ol | APP | 2-Hydroxymethylserine | GAD1 |
| Gancaonin R | APP | ()-alpha-Funebrene | GAD1 |
| 2-methyl-6-ethyl decane | APP | [(1S)-endo]-(-)-Borneol | GAD1 |
| Ethylpalmitate | APP | n-coumaroyltyramine | GAD1 |
| beta-Rhodinol | APP | (S)-camphor | GAD1 |
| 7-TETRADECENE | APP | Vinyl amyl ketone | GAD1 |
| CHEBI:39932 | APP | Scopoletol | NCOA3 |
| Medicarpin | BIRC3 | Methylheptane | NCOA3 |
| Licoflavonol | BIRC3 | beta-Terpinene | NCOA3 |
| Gancaonin P | BIRC3 | Isoviolanthin | NCOA3 |
| Corylifolinin | BIRC3 | WLN: QV4 | NCOA3 |
| m-xylene | GAPDHS | gamma-Hexenol | NCOA3 |
| ISOHEPTANE | GAPDHS | m-xylene | CYP2C9 |
| (1S,2S)-1,2-dimethylcyclopentane | GAPDHS | Amylol | FSHR |
| 2,2-DIMETHYLPENTANE | GAPDHS | Medicarpin | GAD2 |
| (3S)-2,3-dimethylpentane | GAPDHS | nicotiflorin | GAD2 |
| Isohexane | GAPDHS | Narcissoside | GAD2 |
| Cyclobutanol, 1-ethyl- | GAPDHS | Scopoletol | GAD2 |
| 3,3-Dimethylpentane | GAPDHS | glucuronic acid | GAD2 |
| 3-methylheptane | GAPDHS | licorice glycoside E | GAD2 |
| 3-Methylpentane | GAPDHS | licoisoflavanone | GAD2 |
| 5,6,7,8-Tetrahydro-4-methylquinoline | GAPDHS | (-)-Medicocarpin | GAD2 |
| Methyl naphthalene | GAPDHS | violanthin | GAD2 |
| 2-HEXENE | GAPDHS | uralenneoside | GAD2 |
| Glyasperin C | HSD17B1 | Nortangeretin | GAD2 |
| Gancaonin D | HSD17B1 | neoliquiritin | GAD2 |
| Gancaonin Q | HSD17B1 | Isoschaftoside | GAD2 |
| Gancaonin I | HSD17B1 | Licorice glycoside A | GAD2 |
| Glycyrrhiza flavonol A | HSD17B1 | Mipax | GAD2 |
| FITONE | HSD17B1 | Inositol | GAD2 |
| cucurbitacin b | HSD17B1 | vitamin c | GAD2 |
| Elatericin A | HSD17B1 | Leaf acetate | GAD2 |
| Quercimeritrin (6CI,7CI,8CI) | DAO | 5,7-Dihydroxycoumarin | GAD2 |
| (R)-ornithine | MIOX | GUP | GAD2 |
| Karenzu DK2 | CYP7A1 | Docosyl caffeate | HNF4G |
| licochalcone C | CYP7A1 | Medicarpin | IMPA2 |
| vitexin | CYP7A1 | Mairin | RGS18 |
| neoisoliquiritin | CYP7A1 | butylated hydroxytoluene | RGS18 |
| Chlorogenic acid | CYP7A1 | Methylheptane | RGS18 |
| Quercimeritrin (6CI,7CI,8CI) | CYP7A1 | Octadiene | RGS18 |
| 5,7-Dihydroxycoumarin | CYP7A1 | Phaseolinisoflavan | RGS18 |
| Medicarpin | FN1 | licochalcone C | RGS18 |
| licoisoflavanone | FN1 | liquoric acid | RGS18 |
| GUP | FN1 | Gancaonin R | RGS18 |
| Medicarpin | DHFR | Gancaonin S | RGS18 |
| glucuronic acid | DHFR | Gancaonin U | RGS18 |
| licoisoflavanone | DHFR | 5,6,7,8-Tetrahydro-2,4-dimethylquinoline | RGS18 |
| Inositol | DHFR | shinpterocarpin | RGS18 |
| D-Asparaginsaeure | DHFR | 3,22-Dihydroxy-11-oxo-delta(12)-oleanene-27-alpha-methoxycarbonyl-29-oic acid | RGS18 |
| GUP | DHFR | Hispaglabridin B | RGS18 |
| protocatechuic acid | NR1I3 | Glabridin | RGS18 |
| Morusin | NR1I3 | 2-methyl-6-ethyl decane | RGS18 |
| DFV | NR1I3 | (E)-dodec-2-ene | RGS18 |
| Medicarpin | NR1I3 | 2-Tetradecanone | RGS18 |
| Narcissoside | NR1I3 | isoglabrolide | RGS18 |
| 8-Prenylwighteone | NR1I3 | 3'-Hydroxy-4'-O-Methylglabridin | RGS18 |
| 3-Hydroxyglabrol | NR1I3 | 2-Ethyl-p-xylene | RGS18 |
| 18beta-glycyrrhetinic acid | NR1I3 | 3'-Methoxyglabridin | RGS18 |
| licoagropin | NR1I3 | 2,6,10-trimethyl-dodecane | RGS18 |
| (E)-1-[2,4-dihydroxy-3-(3-methylbut-2-enyl)phenyl]-3-[4-hydroxy-3-(3-methylbut-2-enyl)phenyl]prop-2-en-1-one | NR1I3 | Licoriisoflavan A | RGS18 |
| glabrol | NR1I3 | 7-Acetoxy-2-methylisoflavone | RGS18 |
| apioglycyrrhizin | NR1I3 | 12-methyltetradecanoate | RGS18 |
| liquoric acid | NR1I3 | Licoagrocarpin | RGS18 |
| licorice glycoside E | NR1I3 | Kanzonol E | RGS18 |
| Gancaonin S | NR1I3 | Licoagroisoflavone | RGS18 |
| 5,6,7,8-Tetrahydro-2,4-dimethylquinoline | NR1I3 | Xambioona | RGS18 |
| (E)-1-[2,4-dihydroxy-3-(3-methylbut-2-enyl)phenyl]-3-(2,4-dihydroxyphenyl)prop-2-en-1-one | NR1I3 | DIBP | RGS18 |
| Licocoumarone | NR1I3 | PENTYLFURAN | RGS18 |
| glycyrrhetol | NR1I3 | poriferast-5-en-3beta-ol | RGS18 |
| liquiritin | NR1I3 | Insularine | RGS18 |
| 3,22-Dihydroxy-11-oxo-delta(12)-oleanene-27-alpha-methoxycarbonyl-29-oic acid | NR1I3 | Moracin D | RGS18 |
| Glabranin | NR1I3 | Moracin E | RGS18 |
| Docosyl caffeate | NR1I3 | Moracin G | RGS18 |
| violanthin | NR1I3 | Moracin H | RGS18 |
| neoliquiritin | NR1I3 | Mulberrofuran B | RGS18 |
| licuraside | NR1I3 | linolenic acid | RGS18 |
| Liquiritin apioside | NR1I3 | Heptadekan | RGS18 |
| isoglabrolide | NR1I3 | Ethylpalmitate | RGS18 |
| HMO | NR1I3 | Oktadekan | RGS18 |
| 24-Hydroxyglycyrrhetic acid | NR1I3 | FITONE | RGS18 |
| 2-Ethyl-p-xylene | NR1I3 | Isocaryophyllene | RGS18 |
| Licoriisoflavan A | NR1I3 | [(3S)-3,7-dimethylocta-1,6-dien-3-yl] propanoate | RGS18 |
| icos-5-enoic acid | NR1I3 | 1-Tetradecene | RGS18 |
| gadelaidic acid | NR1I3 | Tetracosane | RGS18 |
| Artonin E | NR1I3 | PENTACOSANE | RGS18 |
| beta-Glycyrrhetinic acid | NR1I3 | (4S)-1-methyl-4-(6-methylhepta-1,5-dien-2-yl)cyclohexene | RGS18 |
| Corylifolinin | NR1I3 | Damascenone | RGS18 |
| poriferast-5-en-3beta-ol | NR1I3 | 2,6-Dimethyl-1,3,5,7-octatetraene, E,E- | RGS18 |
| Isobutyrylmallotochromene | NR1I3 | .alpha.-Ionene | RGS18 |
| Moracin H | NR1I3 | Psi-cumene | RGS18 |
| Mulberrofuran B | NR1I3 | 2,6,11-trimethyldodecane | RGS18 |
| linolenic acid | NR1I3 | 1,2-DIHYDRO-1,5,8-TRIMETHYLNAPHTHALENE | RGS18 |
| kuwanon c | NR1I3 | Isoamyl benzoate | RGS18 |
| Ethylpalmitate | NR1I3 | bergamotene (Z,.alpha.,cis) | RGS18 |
| TWT | NR1I3 | Nerylacetone | RGS18 |
| Isocaryophyllene | NR1I3 | poriferast-7-en-3beta-ol | RGS18 |
| Nonadecene | NR1I3 | 2-Hydroxymethylserine | RGS18 |
| UPL | NR1I3 | ()-alpha-Funebrene | RGS18 |
| vitamin c | NR1I3 | 2-Methylnonan-3-one | RGS18 |
| Quercimeritrin (6CI,7CI,8CI) | NR1I3 | 7-TETRADECENE | RGS18 |
| Tetracosane | NR1I3 | PENTADECYLIC ACID | RGS18 |
| Damascenone | NR1I3 | Yamogenin | RGS18 |
| .alpha.-Ionene | NR1I3 | CHEBI:39932 | RGS18 |
| Psi-cumene | NR1I3 | (S)-camphor | RGS18 |
| Linolenic acid ethyl ester | NR1I3 | echinatin | HMGCR |
| 1,2-DIHYDRO-1,5,8-TRIMETHYLNAPHTHALENE | NR1I3 | Licochalcone B | HMGCR |
| Henicosanoic acid | NR1I3 | licochalcone C | HMGCR |
| D-Asparaginsaeure | NR1I3 | 3,4,3',4'-Tetrahydroxy-2-methoxychalcone | HMGCR |
| Linolenic acid methyl ester | NR1I3 | Mairin | NR3C2 |
| 1,2-DIHYDRO-1,5,8-TRIMETHYLNAPHTHALENE | NR1I3 | isorhamnetin | NR3C2 |
| Henicosanoic acid | NR1I3 | rutin | NR3C2 |
| D-Asparaginsaeure | NR1I3 | kaempferol | NR3C2 |
| Linolenic acid methyl ester | NR1I3 | naringenin | NR3C2 |
| morusignin L | NR1I3 | Glyasperin C | NR3C2 |
| cucurbitacin b | NR1I3 | (E)-1-(2,4-dihydroxyphenyl)-3-(2,2-dimethylchromen-6-yl)prop-2-en-1-one | NR3C2 |
| (2R,3R,4S,5S,6R)-2-[[(3S,5S,9R,10S,13R,14R,17R)-17-[(E,2R,5S)-5-ethyl-6-methylhept-3-en-2-yl]-10,13-dimethyl-2,3,4,5,6,9,11,12,14,15,16,17-dodecahydro-1H-cyclopenta[a]phenanthren-3-yl]oxy]-6-(hydroxymethyl)oxane-3,4,5-triol | NR1I3 | (2R)-1-[2,4-dihydroxy-5-(3-methylbut-2-enyl)phenyl]-2-hydroxy-3-[4-hydroxy-3-(3-methylbut-2-enyl)phenyl]propan-1-one | NR3C2 |
| poriferast-7-en-3beta-ol | NR1I3 | licoagropin | NR3C2 |
| (R)-ornithine | NR1I3 | (E)-1-[2,4-dihydroxy-3-(3-methylbut-2-enyl)phenyl]-3-[4-hydroxy-3-(3-methylbut-2-enyl)phenyl]prop-2-en-1-one | NR3C2 |
| Schottenol glucoside | NR1I3 | Gancaonin D | NR3C2 |
| 2-Hydroxymethylserine | NR1I3 | Gancaonin P | NR3C2 |
| Licochalcone B | PPY | Gancaonin Q | NR3C2 |
| Chlorogenic acid | PPY | (E)-1-[2,4-dihydroxy-3-(3-methylbut-2-enyl)phenyl]-3-(2,4-dihydroxyphenyl)prop-2-en-1-one | NR3C2 |
| oleanolic acid | HMOX1 | Licoisoflavone B | NR3C2 |
| glyasperin B | HMOX1 | (E)-3-[3,4-dihydroxy-5-(3-methylbut-2-enyl)phenyl]-1-(2,4-dihydroxyphenyl)prop-2-en-1-one | NR3C2 |
| (E)-1-(2,4-dihydroxyphenyl)-3-(2,2-dimethylchromen-6-yl)prop-2-en-1-one | HMOX1 | 3,22-Dihydroxy-11-oxo-delta(12)-oleanene-27-alpha-methoxycarbonyl-29-oic acid | NR3C2 |
| licoagropin | HMOX1 | Uralenol | NR3C2 |
| (E)-1-[2,4-dihydroxy-3-(3-methylbut-2-enyl)phenyl]-3-[4-hydroxy-3-(3-methylbut-2-enyl)phenyl]prop-2-en-1-one | HMOX1 | neoliquiritin | NR3C2 |
| licochalcone G | HMOX1 | 3,4,3',4'-Tetrahydroxy-2-methoxychalcone | NR3C2 |
| Gancaonin B | HMOX1 | Licoriisoflavan A | NR3C2 |
| Gancaonin Q | HMOX1 | 6″-O-Acetylliquiritin | NR3C2 |
| Licoricidin | HMOX1 | Gancaonin H | NR3C2 |
| (E)-1-[2,4-dihydroxy-3-(3-methylbut-2-enyl)phenyl]-3-(2,4-dihydroxyphenyl)prop-2-en-1-one | HMOX1 | Odoratin | NR3C2 |
| (E)-3-[3,4-dihydroxy-5-(3-methylbut-2-enyl)phenyl]-1-(2,4-dihydroxyphenyl)prop-2-en-1-one | HMOX1 | quercetin | NR3C2 |
| licopyranocoumarin | HMOX1 | poriferast-5-en-3beta-ol | NR3C2 |
| Eurycarpin A | HMOX1 | eugenol | NR3C2 |
| Docosyl caffeate | HMOX1 | Moracin B | NR3C2 |
| Uralenol | HMOX1 | Moracin F | NR3C2 |
| 1-Methoxyficifolinol | HMOX1 | Moracin G | NR3C2 |
| icos-5-enoic acid | HMOX1 | Morindin | NR3C2 |
| Kanzonol H | HMOX1 | FITONE | NR3C2 |
| Gancaonin H | HMOX1 | DEP | NR3C2 |
| Glycyrrhiza flavonol A | HMOX1 | Ethyl caffeate | NR3C2 |
| Mulberrofuran B | HMOX1 | Isotrifolin | NR3C2 |
| TWT | HMOX1 | Tetramethoxyluteolin | NR3C2 |
| Nonadecene | HMOX1 | (2R,3R,4S,5S,6R)-2-[[(3S,5S,9R,10S,13R,14R,17R)-17-[(E,2R,5S)-5-ethyl-6-methylhept-3-en-2-yl]-10,13-dimethyl-2,3,4,5,6,9,11,12,14,15,16,17-dodecahydro-1H-cyclopenta[a]phenanthren-3-yl]oxy]-6-(hydroxymethyl)oxane-3,4,5-triol | NR3C2 |
| UPL | HMOX1 | DIHYDROCUCURBITACIN B | NR3C2 |
| Tetracosane | HMOX1 | Schottenol glucoside | NR3C2 |
| DEP | HMOX1 | n-coumaroyltyramine | NR3C2 |
| DLA | HMOX1 | Izoforon | RAC1 |
| PENTACOSANE | HMOX1 | Mairin | RAC1 |
| Octadecanal | HMOX1 | oleanolic acid | RAC1 |
| Henicosanoic acid | HMOX1 | butylated hydroxytoluene | RAC1 |
| morusignin L | HMOX1 | 2-Caren-10-al | RAC1 |
| cucurbitacin b | HMOX1 | Methylheptane | RAC1 |
| poriferast-7-en-3beta-ol | HMOX1 | beta-Terpinene | RAC1 |
| 2-Hydroxymethylserine | HMOX1 | Glyasperin C | RAC1 |
| Medicarpin | PLA2G1B | Octadiene | RAC1 |
| Inositol | PLA2G1B | (E)-1-[2,4-dihydroxy-3-(3-methylbut-2-enyl)phenyl]-3-[4-hydroxy-3-(3-methylbut-2-enyl)phenyl]prop-2-en-1-one | RAC1 |
| anethole | SRC | Phaseolinisoflavan | RAC1 |
| WLN: VH6 | SRC | Licoflavonol | RAC1 |
| 1-hexanol | SRC | Gancaonin P | RAC1 |
| PTL | SRC | Gancaonin Q | RAC1 |
| Sulcatone | SRC | Gancaonin R | RAC1 |
| Amylol | SRC | Licoricidin | RAC1 |
| I-Amyl acetate | SRC | Glycycoumarin | RAC1 |
| 2-Hexenol | SRC | Glycyrin | RAC1 |
| gamma-Hexenol | SRC | (E)-1-[2,4-dihydroxy-3-(3-methylbut-2-enyl)phenyl]-3-(2,4-dihydroxyphenyl)prop-2-en-1-one | RAC1 |
| 3,5-Octadien-2-one, (E,E)- | SRC | shinpterocarpin | RAC1 |
| formononetin | RAC3 | Hispaglabridin B | RAC1 |
| Castanin | RAC3 | Glabridin | RAC1 |
| beta-Terpinene | RAC3 | 2-methyl-6-ethyl decane | RAC1 |
| (E)-1-butoxyhex-2-ene | RAC3 | (E)-dodec-2-ene | RAC1 |
| euchrenone | RAC3 | 2-Tetradecanone | RAC1 |
| glyasperin B | RAC3 | isoglabrolide | RAC1 |
| (E)-1-(2,4-dihydroxyphenyl)-3-(2,2-dimethylchromen-6-yl)prop-2-en-1-one | RAC3 | 3'-Hydroxy-4'-O-Methylglabridin | RAC1 |
| Glepidotin B | RAC3 | 3'-Methoxyglabridin | RAC1 |
| 3-(2-hydroxy-4-methoxyphenyl)-2H-chromen-7-ol | RAC3 | 12-methyltetradecanoate | RAC1 |
| Glypallichalcone | RAC3 | Gancaonin H | RAC1 |
| echinatin | RAC3 | Licoagrocarpin | RAC1 |
| Licochalcone B | RAC3 | Glycyrrhiza flavonol A | RAC1 |
| licochalcone G | RAC3 | Kanzonol E | RAC1 |
| Licoricone | RAC3 | Licoagroisoflavone | RAC1 |
| Gancaonin A | RAC3 | Licorice glycoside A | RAC1 |
| Gancaonin B | RAC3 | Xambioona | RAC1 |
| Gancaonin Q | RAC3 | Astragalin | RAC1 |
| Glycyrin | RAC3 | PENTYLFURAN | RAC1 |
| Glyzaglabrin | RAC3 | 2-heptanone | RAC1 |
| Glabrene | RAC3 | Methyleugenol | RAC1 |
| Glabrone | RAC3 | eugenol | RAC1 |
| Uralenol-3-methylether | RAC3 | Chlorogenic acid | RAC1 |
| 1-Methoxyphaseollidin | RAC3 | 5-Hydroxycoumarin | RAC1 |
| 3,4,3',4'-Tetrahydroxy-2-methoxychalcone | RAC3 | 1-hexanol | RAC1 |
| licochalcone a | RAC3 | Heptadekan | RAC1 |
| Daidzein dimethyl ether | RAC3 | Ethylpalmitate | RAC1 |
| 4,2',4',alpha-Tetrahydroxydihydrochalcone | RAC3 | Oktadekan | RAC1 |
| 7,2',4'-Trihydroxy-5-methoxy-3-phenylcoumarin | RAC3 | Isocaryophyllene | RAC1 |
| Vestitol | RAC3 | [(3S)-3,7-dimethylocta-1,6-dien-3-yl] propanoate | RAC1 |
| Gancaonin G | RAC3 | 1-Tetradecene | RAC1 |
| Gancaonin H | RAC3 | zoomaric acid | RAC1 |
| Gancaonin I | RAC3 | hexanoic acid | RAC1 |
| Kanzonol E | RAC3 | beta-Ionone | RAC1 |
| Phaseol | RAC3 | PENTACOSANE | RAC1 |
| Chlorogenic acid | RAC3 | Sulcatone | RAC1 |
| 4-Prenylresveratrol | RAC3 | WLN: QV4 | RAC1 |
| Ethyl caffeate | RAC3 | (4S)-1-methyl-4-(6-methylhepta-1,5-dien-2-yl)cyclohexene | RAC1 |
| Norartocarpetin | RAC3 | Amylol | RAC1 |
| Isoamyl benzoate | RAC3 | 2,6-Dimethyl-1,3,5,7-octatetraene, E,E- | RAC1 |
| cucurbitacin b | RAC3 | (7aR)-4,4,7a-trimethyl-6,7-dihydro-5H-benzofuran-2-one | RAC1 |
| Medicarpin | TP53 | Octadecanal | RAC1 |
| isorhamnetin | TP53 | 2,6,11-trimethyldodecane | RAC1 |
| kaempferol | TP53 | Hex-3-enyl acetate | RAC1 |
| Licoflavonol | TP53 | Isoamyl benzoate | RAC1 |
| Gancaonin B | TP53 | 8-methyltocol | RAC1 |
| licorice glycoside E | TP53 | 2-Hexenol | RAC1 |
| Gancaonin P | TP53 | Leaf acetate | RAC1 |
| violanthin | TP53 | Linolenic acid methyl ester | RAC1 |
| neoisoliquiritin | TP53 | bergamotene (Z,.alpha.,cis) | RAC1 |
| Glyasperin A | TP53 | gamma-Hexenol | RAC1 |
| Morindin | TP53 | morusignin L | RAC1 |
| morusignin L | TP53 | ()-alpha-Funebrene | RAC1 |
| cucurbitacin b | TP53 | 2-Methylnonan-3-one | RAC1 |
| Elatericin A | TP53 | 4-methyl-2-hexanone | RAC1 |
| DIHYDROCUCURBITACIN B | TP53 | 7-TETRADECENE | RAC1 |
| protocatechuic acid | LYZ | PENTADECYLIC ACID | RAC1 |
| Uralenol | LYZ | Yamogenin | RAC1 |
| Glyasperin A | LYZ | CHEBI:39932 | RAC1 |
| (-)-Medicocarpin | CNDP1 | (S)-camphor | RAC1 |
| Pinocembrin | NAT1 | Vinyl amyl ketone | RAC1 |
| 7,4'-Dihydroxyflavone | NAT1 | m-xylene | ANXA1 |
| formononetin | NAT1 | Morusin | ANXA1 |
| Calycosin | NAT1 | Izoforon | ANXA1 |
| naringenin | NAT1 | Heptan | ANXA1 |
| Castanin | NAT1 | 8-Prenylwighteone | ANXA1 |
| anethole | NAT1 | Methylheptane | ANXA1 |
| Isotrifoliol | NAT1 | 3-Hydroxyglabrol | ANXA1 |
| (2R)-1-[2,4-dihydroxy-5-(3-methylbut-2-enyl)phenyl]-2-hydroxy-3-[4-hydroxy-3-(3-methylbut-2-enyl)phenyl]propan-1-one | NAT1 | 18beta-glycyrrhetinic acid | ANXA1 |
| WLN: 4OVR | NAT1 | (2R)-1-[2,4-dihydroxy-5-(3-methylbut-2-enyl)phenyl]-2-hydroxy-3-[4-hydroxy-3-(3-methylbut-2-enyl)phenyl]propan-1-one | ANXA1 |
| Licoricone | NAT1 | (E)-1-[2,4-dihydroxy-3-(3-methylbut-2-enyl)phenyl]-3-[4-hydroxy-3-(3-methylbut-2-enyl)phenyl]prop-2-en-1-one | ANXA1 |
| Prunetin | NAT1 | glabrol | ANXA1 |
| Gancaonin D | NAT1 | apioglycyrrhizin | ANXA1 |
| Glycycoumarin | NAT1 | 2,3-dimethylhexane | ANXA1 |
| Glycyrin | NAT1 | Gancaonin R | ANXA1 |
| Glyzaglabrin | NAT1 | Gancaonin S | ANXA1 |
| vitexin | NAT1 | 5,6,7,8-Tetrahydro-2,4-dimethylquinoline | ANXA1 |
| Nortangeretin | NAT1 | Licocoumarone | ANXA1 |
| Isoschaftoside | NAT1 | (4S)-2,4-dimethylhexane | ANXA1 |
| 3'-Methoxyglabridin | NAT1 | glycyrrhetol | ANXA1 |
| Daidzein dimethyl ether | NAT1 | 3,22-Dihydroxy-11-oxo-delta(12)-oleanene-27-alpha-methoxycarbonyl-29-oic acid | ANXA1 |
| 7,2',4'-Trihydroxy-5-methoxy-3-phenylcoumarin | NAT1 | Docosyl caffeate | ANXA1 |
| 7-Acetoxy-2-methylisoflavone | NAT1 | isoglabrolide | ANXA1 |
| Gancaonin I | NAT1 | 24-Hydroxyglycyrrhetic acid | ANXA1 |
| Odoratin | NAT1 | 2-Ethyl-p-xylene | ANXA1 |
| Methyleugenol | NAT1 | 3-methylhexane | ANXA1 |
| eugenol | NAT1 | Licoriisoflavan A | ANXA1 |
| Moracin B | NAT1 | icos-5-enoic acid | ANXA1 |
| Moracin F | NAT1 | gadelaidic acid | ANXA1 |
| Nonanal | NAT1 | Artonin E | ANXA1 |
| Terragon | NAT1 | beta-Glycyrrhetinic acid | ANXA1 |
| beta-Rhodinol | NAT1 | poriferast-5-en-3beta-ol | ANXA1 |
| tectorigenin | NAT1 | Albafuran A | ANXA1 |
| Norartocarpetin | NAT1 | Isobutyrylmallotochromene | ANXA1 |
| Tetramethoxyluteolin | NAT1 | gynesine | ANXA1 |
| methyl 8-methyl-decanoate | NAT1 | linolenic acid | ANXA1 |
| Nerylacetone | NAT1 | kuwanon c | ANXA1 |
| Elatericin A | NAT1 | TWT | ANXA1 |
| DFV | ME1 | Tetracosane | ANXA1 |
| Mairin | ME1 | Fleet-X | ANXA1 |
| butylated hydroxytoluene | ME1 | DEP | ANXA1 |
| Scopoletol | ME1 | DLA | ANXA1 |
| beta-Terpinene | ME1 | beta-Ionone | ANXA1 |
| (E)-1-butoxyhex-2-ene | ME1 | 2,6-Dimethyl-1,3,5,7-octatetraene, E,E- | ANXA1 |
| glyasperin E | ME1 | .alpha.-Ionene | ANXA1 |
| Isotrifoliol | ME1 | Octadecanal | ANXA1 |
| licoagropin | ME1 | Linolenyl alcohol | ANXA1 |
| 3-(2-hydroxy-4-methoxyphenyl)-2H-chromen-7-ol | ME1 | Psi-cumene | ANXA1 |
| Gancaonin U | ME1 | 1,2-DIHYDRO-1,5,8-TRIMETHYLNAPHTHALENE | ANXA1 |
| Gancaonin V | ME1 | Henicosanoic acid | ANXA1 |
| 5,6,7,8-Tetrahydro-2,4-dimethylquinoline | ME1 | 8-methyltocol | ANXA1 |
| Licocoumarone | ME1 | Linolenic acid methyl ester | ANXA1 |
| glycyrrhetol | ME1 | Spinasterol | ANXA1 |
| licopyranocoumarin | ME1 | (2R,3R,4S,5S,6R)-2-[[(3S,5S,9R,10S,13R,14R,17R)-17-[(E,2R,5S)-5-ethyl-6-methylhept-3-en-2-yl]-10,13-dimethyl-2,3,4,5,6,9,11,12,14,15,16,17-dodecahydro-1H-cyclopenta[a]phenanthren-3-yl]oxy]-6-(hydroxymethyl)oxane-3,4,5-triol | ANXA1 |
| Glabranin | ME1 | Schottenol glucoside | ANXA1 |
| 2-methyl-6-ethyl decane | ME1 | (S)-camphor | ANXA1 |
| 4H-1-Benzopyran-4-one, 2-(4-(beta-D-glucopyranosyloxy)phenyl)-2,3-dihydro-5,7-dihydroxy-, (2S)- | ME1 | Narcissoside | GRK6 |
| Pentadecanol | ME1 | Licochalcone B | GRK6 |
| Uralenol-3-methylether | ME1 | liquiritin | GRK6 |
| Nortangeretin | ME1 | uralenneoside | GRK6 |
| Cyclobutanol, 1-ethyl- | ME1 | Isoviolanthin | GRK6 |
| isoglycycoumarin | ME1 | 6″-O-Acetylliquiritin | GRK6 |
| isoglabrolide | ME1 | ecdysterone | GRK6 |
| HMO | ME1 | Moracin B | GRK6 |
| Isoschaftoside | ME1 | Moracin F | GRK6 |
| 1-Methoxyphaseollidin | ME1 | Chlorogenic acid | GRK6 |
| 2-Ethyl-p-xylene | ME1 | Inositol | GRK6 |
| licochalcone a | ME1 | Isotrifolin | GRK6 |
| 1-(5-hydroxy-2,2-dimethylchromen-6-yl)-3-(4-hydroxyphenyl)prop-2-en-1-one | ME1 | n-coumaroyltyramine | GRK6 |
| 2,6,10-trimethyl-dodecane | ME1 | Mairin | PLA2G2A |
| 6″-O-Acetylliquiritin | ME1 | Methylheptane | PLA2G2A |
| 7-Acetoxy-2-methylisoflavone | ME1 | (E)-1-butoxyhex-2-ene | PLA2G2A |
| 12-methyltetradecanoate | ME1 | Glyasperin C | PLA2G2A |
| Vestitol | ME1 | (E)-1-(2,4-dihydroxyphenyl)-3-(2,2-dimethylchromen-6-yl)prop-2-en-1-one | PLA2G2A |
| Licoagrocarpin | ME1 | Octadiene | PLA2G2A |
| Gancaonin I | ME1 | Phaseolinisoflavan | PLA2G2A |
| Glyasperin A | ME1 | liquoric acid | PLA2G2A |
| Licorice glycoside A | ME1 | Gancaonin V | PLA2G2A |
| Xambioona | ME1 | shinpterocarpin | PLA2G2A |
| ecdysterone | ME1 | Glabridin | PLA2G2A |
| 5-Hydroxycoumarin | ME1 | Pentadecanol | PLA2G2A |
| Nonanal | ME1 | (E)-dodec-2-ene | PLA2G2A |
| linolenic acid | ME1 | 2-Tetradecanone | PLA2G2A |
| TWT | ME1 | isoglabrolide | PLA2G2A |
| FITONE | ME1 | 3'-Hydroxy-4'-O-Methylglabridin | PLA2G2A |
| Isocaryophyllene | ME1 | 3'-Methoxyglabridin | PLA2G2A |
| Nonadecene | ME1 | 2,6,10-trimethyl-dodecane | PLA2G2A |
| UPL | ME1 | 6″-O-Acetylliquiritin | PLA2G2A |
| PENTACOSANE | ME1 | 7-Acetoxy-2-methylisoflavone | PLA2G2A |
| Damascenone | ME1 | 12-methyltetradecanoate | PLA2G2A |
| Psi-cumene | ME1 | Kanzonol E | PLA2G2A |
| 2,6,11-trimethyldodecane | ME1 | Licoagroisoflavone | PLA2G2A |
| 1,2-DIHYDRO-1,5,8-TRIMETHYLNAPHTHALENE | ME1 | Xambioona | PLA2G2A |
| methyl 8-methyl-decanoate | ME1 | PENTYLFURAN | PLA2G2A |
| Linolenic acid methyl ester | ME1 | Moracin D | PLA2G2A |
| Nerylacetone | ME1 | Moracin G | PLA2G2A |
| 5,7-Dihydroxycoumarin | ME1 | Moracin H | PLA2G2A |
| CIR | ME1 | Morindin | PLA2G2A |
| 2-Methylnonan-3-one | ME1 | linolenic acid | PLA2G2A |
| PENTADECYLIC ACID | ME1 | Heptadekan | PLA2G2A |
| n-coumaroyltyramine | ME1 | Ethylpalmitate | PLA2G2A |
| Yamogenin | ME1 | Oktadekan | PLA2G2A |
| HMF | ME1 | TWT | PLA2G2A |
| Medicarpin | PPARG | [(3S)-3,7-dimethylocta-1,6-dien-3-yl] propanoate | PLA2G2A |
| 2-Caren-10-al | PPARG | 1-Tetradecene | PLA2G2A |
| Scopoletol | PPARG | PENTACOSANE | PLA2G2A |
| 2-heptanone | PPARG | (4S)-1-methyl-4-(6-methylhepta-1,5-dien-2-yl)cyclohexene | PLA2G2A |
| WLN: VH6 | PPARG | 2,6-Dimethyl-1,3,5,7-octatetraene, E,E- | PLA2G2A |
| 5-Hydroxycoumarin | PPARG | .alpha.-Ionene | PLA2G2A |
| 6-Hydroxycoumarin | PPARG | 2,6,11-trimethyldodecane | PLA2G2A |
| 1-hexanol | PPARG | Henicosanoic acid | PLA2G2A |
| Majudin | PPARG | methyl 8-methyl-decanoate | PLA2G2A |
| PTL | PPARG | Linolenic acid methyl ester | PLA2G2A |
| Sulcatone | PPARG | bergamotene (Z,.alpha.,cis) | PLA2G2A |
| Iva | PPARG | Elatericin A | PLA2G2A |
| WLN: QV4 | PPARG | Schottenol glucoside | PLA2G2A |
| Amylol | PPARG | ()-alpha-Funebrene | PLA2G2A |
| naphthalene | PPARG | 2-Methylnonan-3-one | PLA2G2A |
| (7aR)-4,4,7a-trimethyl-6,7-dihydro-5H-benzofuran-2-one | PPARG | 7-TETRADECENE | PLA2G2A |
| I-Amyl acetate | PPARG | Yamogenin | PLA2G2A |
| 2-Hexenol | PPARG | CHEBI:39932 | PLA2G2A |
| gamma-Hexenol | PPARG | Scopoletol | CTSD |
| 3,5-Octadien-2-one, (E,E)- | PPARG | 2-Caren-10-al | RBKS |
| nicotiflorin | MT-CYB | Scopoletol | RBKS |
| isorhamnetin | MT-CYB | Mipax | RBKS |
| formononetin | MT-CYB | 2-heptanone | RBKS |
| rutin | MT-CYB | WLN: VH6 | RBKS |
| Hirsutrin | MT-CYB | Majudin | RBKS |
| (E)-1-(2,4-dihydroxyphenyl)-3-(2,2-dimethylchromen-6-yl)prop-2-en-1-one | MT-CYB | I-Amyl acetate | RBKS |
| licoagropin | MT-CYB | 2-Hexenol | RBKS |
| (2S)-6-(2,4-dihydroxyphenyl)-2-(2-hydroxypropan-2-yl)-4-methoxy-2,3-dihydrofuro[3,2-g]chromen-7-one | MT-CYB | Leaf acetate | RBKS |
| Glepidotin A | MT-CYB | Vinyl amyl ketone | RBKS |
| Glepidotin B | MT-CYB | DFV | PPARA |
| 3-(2-hydroxy-4-methoxyphenyl)-2H-chromen-7-ol | MT-CYB | Mairin | PPARA |
| Karenzu DK2 | MT-CYB | butylated hydroxytoluene | PPARA |
| Licochalcone B | MT-CYB | 8-Prenylwighteone | PPARA |
| Gancaonin U | MT-CYB | (E)-1-butoxyhex-2-ene | PPARA |
| (E)-3-[3,4-dihydroxy-5-(3-methylbut-2-enyl)phenyl]-1-(2,4-dihydroxyphenyl)prop-2-en-1-one | MT-CYB | 18beta-glycyrrhetinic acid | PPARA |
| Isoviolanthin | MT-CYB | glyasperin E | PPARA |
| licuraside | MT-CYB | Prunetin | PPARA |
| 1-Methoxyphaseollidin | MT-CYB | Gancaonin D | PPARA |
| 3,4,3',4'-Tetrahydroxy-2-methoxychalcone | MT-CYB | Gancaonin U | PPARA |
| Daidzein dimethyl ether | MT-CYB | glycyrrhetol | PPARA |
| 1-Methoxyficifolinol | MT-CYB | liquiritin | PPARA |
| 4,2',4',alpha-Tetrahydroxydihydrochalcone | MT-CYB | Glabranin | PPARA |
| 1-(5-hydroxy-2,2-dimethylchromen-6-yl)-3-(4-hydroxyphenyl)prop-2-en-1-one | MT-CYB | Glabrene | PPARA |
| Vestitol | MT-CYB | 2-methyl-6-ethyl decane | PPARA |
| Licorice glycoside A | MT-CYB | (E)-dodec-2-ene | PPARA |
| Astragalin | MT-CYB | Liquiritin apioside | PPARA |
| 2-heptanone | MT-CYB | isoglabrolide | PPARA |
| quercetin | MT-CYB | 1-Methoxyphaseollidin | PPARA |
| Methyleugenol | MT-CYB | 2,6,10-trimethyl-dodecane | PPARA |
| eugenol | MT-CYB | gadelaidic acid | PPARA |
| 5-Hydroxycoumarin | MT-CYB | beta-Glycyrrhetinic acid | PPARA |
| Quercimeritrin (6CI,7CI,8CI) | MT-CYB | Licoagroisoflavone | PPARA |
| Ethyl caffeate | MT-CYB | Phaseol | PPARA |
| Isotrifolin | MT-CYB | Insularine | PPARA |
| Tetramethoxyluteolin | MT-CYB | linolenic acid | PPARA |
| Hex-3-enyl acetate | MT-CYB | TWT | PPARA |
| [(1S)-endo]-(-)-Borneol | MT-CYB | FITONE | PPARA |
| n-coumaroyltyramine | MT-CYB | Nonadecene | PPARA |
| HMF | MT-CYB | UPL | PPARA |
| Vinyl amyl ketone | MT-CYB | Tetracosane | PPARA |
| Medicarpin | PNLIP | PENTACOSANE | PPARA |
| anethole | GM2A | beta-Rhodinol | PPARA |
| 5,6,7,8-Tetrahydro-4-methylquinoline | GM2A | tectorigenin | PPARA |
| 2-Caren-10-al | SULT2A1 | 2,6,11-trimethyldodecane | PPARA |
| Gancaonin C | SULT2A1 | Linolenic acid ethyl ester | PPARA |
| Glycycoumarin | SULT2A1 | ()-alpha-Funebrene | PPARA |
| Glycyrin | SULT2A1 | nicotiflorin | DNMT1 |
| Glabridin | SULT2A1 | rutin | DNMT1 |
| 2-Tetradecanone | SULT2A1 | Hirsutrin | DNMT1 |
| 7-Acetoxy-2-methylisoflavone | SULT2A1 | beta-Terpinene | DNMT1 |
| [(3S)-3,7-dimethylocta-1,6-dien-3-yl] propanoate | SULT2A1 | Isotrifoliol | DNMT1 |
| hexanoic acid | SULT2A1 | 3-(2-hydroxy-4-methoxyphenyl)-2H-chromen-7-ol | DNMT1 |
| beta-Rhodinol | SULT2A1 | Gancaonin B | DNMT1 |
| Hex-3-enyl acetate | SULT2A1 | Gancaonin C | DNMT1 |
| methyl 8-methyl-decanoate | SULT2A1 | Gancaonin D | DNMT1 |
| Nerylacetone | SULT2A1 | vitexin | DNMT1 |
| [(1S)-endo]-(-)-Borneol | SULT2A1 | Isoviolanthin | DNMT1 |
| Morusin | CYP2E1 | Isoschaftoside | DNMT1 |
| Mairin | CYP2E1 | Artonin E | DNMT1 |
| 8-Prenylwighteone | CYP2E1 | Vestitol | DNMT1 |
| 18beta-glycyrrhetinic acid | CYP2E1 | Astragalin | DNMT1 |
| glyasperin B | CYP2E1 | Morindin | DNMT1 |
| Glyasperin C | CYP2E1 | kuwanon c | DNMT1 |
| (E)-1-(2,4-dihydroxyphenyl)-3-(2,2-dimethylchromen-6-yl)prop-2-en-1-one | CYP2E1 | Quercimeritrin (6CI,7CI,8CI) | DNMT1 |
| licoagropin | CYP2E1 | cucurbitacin b | DNMT1 |
| (E)-1-[2,4-dihydroxy-3-(3-methylbut-2-enyl)phenyl]-3-[4-hydroxy-3-(3-methylbut-2-enyl)phenyl]prop-2-en-1-one | CYP2E1 | Elatericin A | DNMT1 |
| licochalcone C | CYP2E1 | DIHYDROCUCURBITACIN B | DNMT1 |
| glabrol | CYP2E1 | n-coumaroyltyramine | DNMT1 |
| licochalcone G | CYP2E1 | nicotiflorin | DNMT1 |
| Gancaonin D | CYP2E1 | rutin | DNMT1 |
| Gancaonin S | CYP2E1 | Hirsutrin | DNMT1 |
| Gancaonin U | CYP2E1 | beta-Terpinene | DNMT1 |
| Licoricidin | CYP2E1 | Isotrifoliol | DNMT1 |
| (E)-1-[2,4-dihydroxy-3-(3-methylbut-2-enyl)phenyl]-3-(2,4-dihydroxyphenyl)prop-2-en-1-one | CYP2E1 | 3-(2-hydroxy-4-methoxyphenyl)-2H-chromen-7-ol | DNMT1 |
| Licocoumarone | CYP2E1 | Gancaonin B | DNMT1 |
| (E)-3-[3,4-dihydroxy-5-(3-methylbut-2-enyl)phenyl]-1-(2,4-dihydroxyphenyl)prop-2-en-1-one | CYP2E1 | Gancaonin C | DNMT1 |
| glycyrrhetol | CYP2E1 | Gancaonin D | DNMT1 |
| 3,22-Dihydroxy-11-oxo-delta(12)-oleanene-27-alpha-methoxycarbonyl-29-oic acid | CYP2E1 | vitexin | DNMT1 |
| Nortangeretin | CYP2E1 | Isoviolanthin | DNMT1 |
| isoglabrolide | CYP2E1 | Isoschaftoside | DNMT1 |
| HMO | CYP2E1 | Artonin E | DNMT1 |
| 3'-Hydroxy-4'-O-Methylglabridin | CYP2E1 | Vestitol | DNMT1 |
| licochalcone a | CYP2E1 | Astragalin | DNMT1 |
| 3'-Methoxyglabridin | CYP2E1 | Morindin | DNMT1 |
| 1-Methoxyficifolinol | CYP2E1 | kuwanon c | DNMT1 |
| 1-(5-hydroxy-2,2-dimethylchromen-6-yl)-3-(4-hydroxyphenyl)prop-2-en-1-one | CYP2E1 | Quercimeritrin (6CI,7CI,8CI) | DNMT1 |
| Licoriisoflavan A | CYP2E1 | cucurbitacin b | DNMT1 |
| Artonin E | CYP2E1 | Elatericin A | DNMT1 |
| Vestitol | CYP2E1 | DIHYDROCUCURBITACIN B | DNMT1 |
| Gancaonin H | CYP2E1 | n-coumaroyltyramine | DNMT1 |
| beta-Glycyrrhetinic acid | CYP2E1 | m-xylene | ABAT |
| Glycyrrhiza flavonol A | CYP2E1 | p-xylene | ABAT |
| DIBP | CYP2E1 | uralsaponin B | ABAT |
| poriferast-5-en-3beta-ol | CYP2E1 | Mipax | ABAT |
| Insularine | CYP2E1 | Inositol | ABAT |
| Isobutyrylmallotochromene | CYP2E1 | 1,3,8-p-Menthatriene | ABAT |
| UPL | CYP2E1 | 5,7-Dihydroxycoumarin | ABAT |
| Tetracosane | CYP2E1 | protocatechuic acid | LTF |
| DLA | CYP2E1 | Scopoletol | LTF |
| tectorigenin | CYP2E1 | euchrenone | LTF |
| Linolenyl alcohol | CYP2E1 | Glepidotin A | LTF |
| Elatericin A | CYP2E1 | 5-Hydroxycoumarin | LTF |
| poriferast-7-en-3beta-ol | CYP2E1 | 5,7-Dihydroxycoumarin | LTF |
| 2-Hydroxymethylserine | CYP2E1 | m-xylene | DDC |
| Medicarpin | ADH6 | Morusin | DDC |
| 4,2',4',alpha-Tetrahydroxydihydrochalcone | ADH6 | Izoforon | DDC |
| Inositol | ADH6 | Heptan | DDC |
| gamma-aminobutyric acid | ADH6 | 2-Caren-10-al | DDC |
| m-xylene | PTGS1 | 8-Prenylwighteone | DDC |
| Izoforon | PTGS1 | 3-Hydroxyglabrol | DDC |
| Heptan | PTGS1 | 18beta-glycyrrhetinic acid | DDC |
| Medicarpin | PTGS1 | (2R)-1-[2,4-dihydroxy-5-(3-methylbut-2-enyl)phenyl]-2-hydroxy-3-[4-hydroxy-3-(3-methylbut-2-enyl)phenyl]propan-1-one | DDC |
| oleanolic acid | PTGS1 | (E)-1-[2,4-dihydroxy-3-(3-methylbut-2-enyl)phenyl]-3-[4-hydroxy-3-(3-methylbut-2-enyl)phenyl]prop-2-en-1-one | DDC |
| formononetin | PTGS1 | glabrol | DDC |
| rutin | PTGS1 | 2,3-dimethylhexane | DDC |
| Castanin | PTGS1 | Gancaonin R | DDC |
| (2R)-1-[2,4-dihydroxy-5-(3-methylbut-2-enyl)phenyl]-2-hydroxy-3-[4-hydroxy-3-(3-methylbut-2-enyl)phenyl]propan-1-one | PTGS1 | Gancaonin S | DDC |
| licoagropin | PTGS1 | 5,6,7,8-Tetrahydro-2,4-dimethylquinoline | DDC |
| (E)-1-[2,4-dihydroxy-3-(3-methylbut-2-enyl)phenyl]-3-[4-hydroxy-3-(3-methylbut-2-enyl)phenyl]prop-2-en-1-one | PTGS1 | (4S)-2,4-dimethylhexane | DDC |
| licochalcone C | PTGS1 | glycyrrhetol | DDC |
| apioglycyrrhizin | PTGS1 | 3,22-Dihydroxy-11-oxo-delta(12)-oleanene-27-alpha-methoxycarbonyl-29-oic acid | DDC |
| licochalcone G | PTGS1 | Docosyl caffeate | DDC |
| Licoricone | PTGS1 | 24-Hydroxyglycyrrhetic acid | DDC |
| 2,3-dimethylhexane | PTGS1 | 2-Ethyl-p-xylene | DDC |
| (E)-1-[2,4-dihydroxy-3-(3-methylbut-2-enyl)phenyl]-3-(2,4-dihydroxyphenyl)prop-2-en-1-one | PTGS1 | Licoriisoflavan A | DDC |
| (4S)-2,4-dimethylhexane | PTGS1 | icos-5-enoic acid | DDC |
| (E)-3-[3,4-dihydroxy-5-(3-methylbut-2-enyl)phenyl]-1-(2,4-dihydroxyphenyl)prop-2-en-1-one | PTGS1 | Kanzonol H | DDC |
| Cyclobutanol, 1-ethyl- | PTGS1 | Artonin E | DDC |
| HMO | PTGS1 | beta-Glycyrrhetinic acid | DDC |
| 3-methylhexane | PTGS1 | 2-heptanone | DDC |
| Daidzein dimethyl ether | PTGS1 | poriferast-5-en-3beta-ol | DDC |
| Kanzonol H | PTGS1 | Isobutyrylmallotochromene | DDC |
| Gancaonin H | PTGS1 | Mulberrofuran B | DDC |
| Glyasperin A | PTGS1 | gynesine | DDC |
| Corylifolinin | PTGS1 | 5-Hydroxycoumarin | DDC |
| Isobutyrylmallotochromene | PTGS1 | kuwanon c | DDC |
| Moracin F | PTGS1 | Nonadecene | DDC |
| 6-Hydroxycoumarin | PTGS1 | UPL | DDC |
| Nonadecene | PTGS1 | zoomaric acid | DDC |
| zoomaric acid | PTGS1 | Tetracosane | DDC |
| Fleet-X | PTGS1 | Fleet-X | DDC |
| Majudin | PTGS1 | DEP | DDC |
| hexanoic acid | PTGS1 | Sulcatone | DDC |
| beta-Ionone | PTGS1 | (7aR)-4,4,7a-trimethyl-6,7-dihydro-5H-benzofuran-2-one | DDC |
| Sulcatone | PTGS1 | .alpha.-Ionene | DDC |
| WLN: QV4 | PTGS1 | Octadecanal | DDC |
| Amylol | PTGS1 | Linolenyl alcohol | DDC |
| Hex-3-enyl acetate | PTGS1 | Psi-cumene | DDC |
| 8-methyltocol | PTGS1 | Linolenic acid ethyl ester | DDC |
| gamma-Hexenol | PTGS1 | 1,2-DIHYDRO-1,5,8-TRIMETHYLNAPHTHALENE | DDC |
| [(1S)-endo]-(-)-Borneol | PTGS1 | Henicosanoic acid | DDC |
| Medicarpin | MMP9 | I-Amyl acetate | DDC |
| 2-Caren-10-al | MMP9 | Leaf acetate | DDC |
| glucuronic acid | MMP9 | Spinasterol | DDC |
| licoisoflavanone | MMP9 | (2R,3R,4S,5S,6R)-2-[[(3S,5S,9R,10S,13R,14R,17R)-17-[(E,2R,5S)-5-ethyl-6-methylhept-3-en-2-yl]-10,13-dimethyl-2,3,4,5,6,9,11,12,14,15,16,17-dodecahydro-1H-cyclopenta[a]phenanthren-3-yl]oxy]-6-(hydroxymethyl)oxane-3,4,5-triol | DDC |
| 2-heptanone | MMP9 | poriferast-7-en-3beta-ol | DDC |
| Chlorogenic acid | MMP9 | Schottenol glucoside | DDC |
| Inositol | MMP9 | 2-Hydroxymethylserine | DDC |
| 5-Hydroxycoumarin | MMP9 | 4-methyl-2-hexanone | DDC |
| vitamin c | MMP9 | Yamogenin | DDC |
| zoomaric acid | MMP9 | (S)-camphor | DDC |
| Sulcatone | MMP9 | Vinyl amyl ketone | DDC |
| (7aR)-4,4,7a-trimethyl-6,7-dihydro-5H-benzofuran-2-one | MMP9 | protocatechuic acid | HBA1 |
| I-Amyl acetate | MMP9 | 1-hexanol | HBA1 |
| GUP | MMP9 | Amylol | HBA1 |
| 4-methyl-2-hexanone | MMP9 | 2-Hexenol | HBA1 |
|  |  | gamma-Hexenol | HBA1 |
